# Amphiphilic dynamic covalent polymer vectors of siRNA†

**DOI:** 10.1039/d4sc07668k

**Published:** 2024-12-30

**Authors:** José García Coll, Pauline Trousselier, Sachin Dattram Pawar, Yannick Bessin, Laure Lichon, Jeanne Leblond Chain, Emmanuelle Sachon, Nadir Bettache, Sébastien Ulrich

**Affiliations:** a Institut des Biomolécules Max Mousseron (IBMM), Université de Montpellier, CNRS, ENSCM Montpellier France nadir.bettache@umontpellier.fr sebastien.ulrich@cnrs.fr; b Sorbonne Université, École Normale Supérieure, PSL University, CNRS, Laboratoire des Biomolécules (LBM) Paris France emmanuelle.sachon@sorbonne-universite.fr; c Université de Bordeaux, CNRS, Inserm, ARNA, UMR 5320, U1212 Bordeaux France

## Abstract

Dynamic covalent polymers (DCPs) recently emerged as smart siRNA delivery vectors, which dynamically self-assemble through siRNA templating and depolymerize in a controlled manner. Herein, we report the dynamic combinatorial screening of cationic and amphiphilic peptide-based monomers. We provide experimental evidence, by mass spectrometry analyses, of the siRNA-templated formation of DCPs, and show that amphiphilic DCPs display superior activity in terms of siRNA complexation and delivery in cells. Thus, the work describes a new type of siRNA vector based on dynamic covalent lipopolyplexes, which feature improved activity as well as better nano-structuration compared to previous generations of DCPs.

## Abstract 2

Hydrophobization of peptide-based dynamic covalent polymer vectors boosts their siRNA delivery performances.

## Graphical abstract

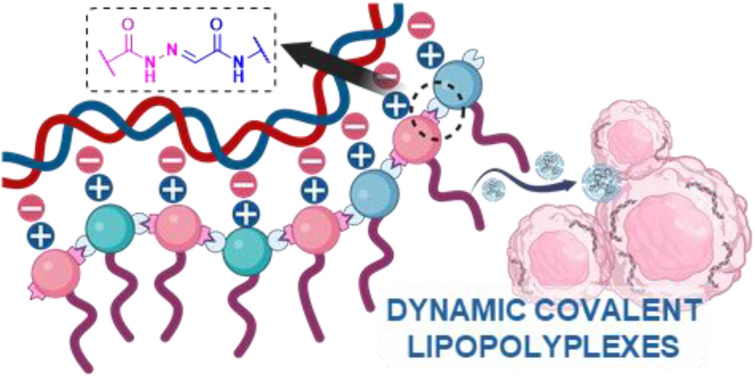

## Introduction

**Fig. 1:**
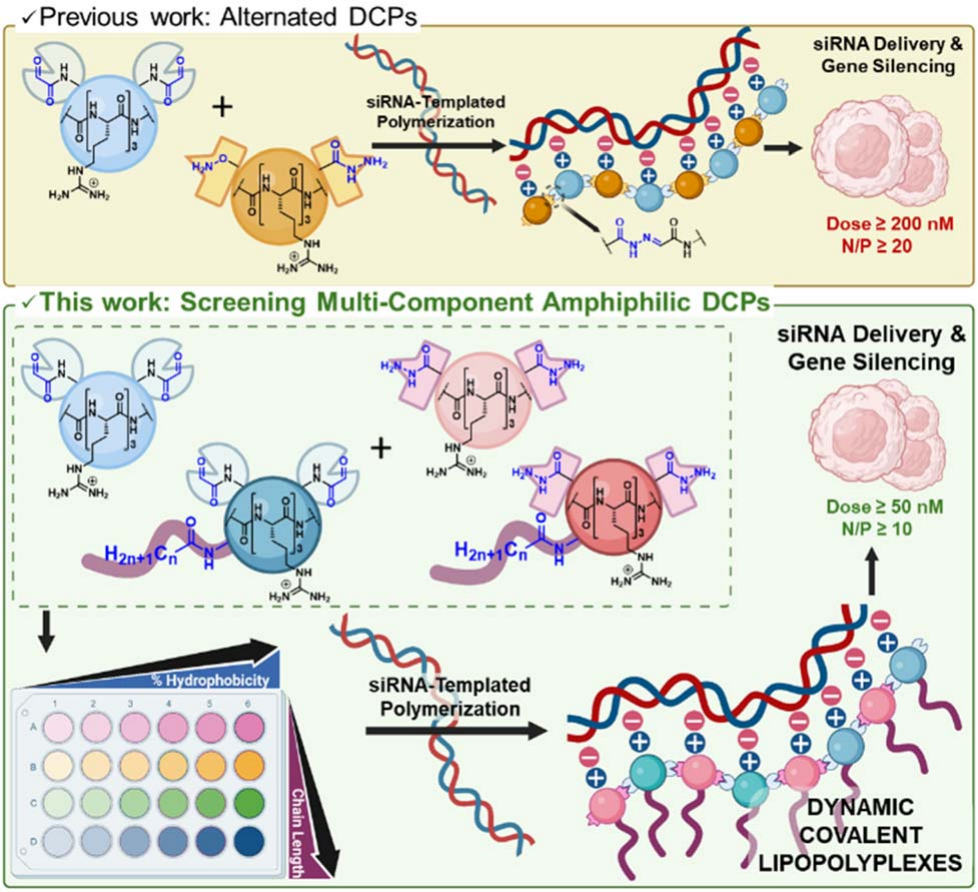
Delivery of siRNA by dynamic covalent polymers (DCPs) which formation occurs through siRNA templating: previous design of cationic DCPs made by combining bisaldehyde and aminooxy-hydrazide peptides (top), and present work introducing increasing amount of amphiphilic monomers featuring various fatty acid tails (*n* = 6, 10, 12, 18), thereby screening dynamic combinatorial libraries of acylhydrazone-based DCPs with increasing percentage of hydrophobicity as well as increasing fatty acid chain length, and identifying dynamic covalent lipopolyplexes (depicted is the system made of 100% amphiphilic monomers) with improved efficacy for siRNA delivery (bottom). Created using BioRender. García Coll J. (2025) https://BioRender.com/c68l352.

RNA interference was discovered in 1998 but it was only in 2018 that small interfering RNA (siRNA) found clinical applications.^1,2^ Since 2018, five siRNA drugs have been approved by the FDA and EMA.^3,4^ These success stories rely extensively on the development of effective non-viral delivery systems based on glycoconjugates or lipid nanoparticles (LNP). Further development in this area will contribute to broadening the scope of application of RNA therapeutics.^5,6^ In that perspective, cationic polymers^7,8^ have received a strong interest but suffer from a limited efficacy, in part due to poor siRNA release.^9^ To address this challenge, we have developed pH-sensitive cationic dynamic covalent polymers (DCPs) which spontaneously depolymerize at the acidic pH found in endosomes,^10^ and can indeed deliver siRNA in live cells.^11^ Furthermore, exploring self-assembly pathways^12^ for accessing dynamic vectors,^13,14^ we recently evidenced that these DCPs can be formed *in situ* through a siRNA-templated process.^15–17^ Such an approach alleviates the process of polyplexes formation and enables more rapid screening of components.^16,18,19^ However, the activity of non-functionalized cationic DCPs as siRNA vectors remains hitherto limited to detectable silencing activity in cell culture at elevated siRNA doses (≥200 nM) and high N/P ratio^20^ (≥20).^16,21^ An opportunity for improving the activity of cationic polymers and dendrimers as siRNA vectors is to exploit hydrophobization. Hydrophobization can indeed contribute to (i) directing the self-assembly toward monodisperse nanoparticles,^22–24^ and (ii) improving cell uptake by stabilizing the interactions with the cell membranes during endocytosis.^25,26^ Tuning the balance of electrostatic and hydrophobic interactions at play during cell penetration is a critical challenge in delivery applications to promote cell penetration without damaging the cell membrane.^27,28^ Herein, we report the synthesis of amphiphilic peptide-based monomers and show that they undergo a siRNA-templated dynamic covalent polymerization, which results in more defined nanoparticles that are able to deliver siRNA in cells with a greater efficacy than previous generations. This work extends thus the pioneering reports from the Matile and Montenegro groups on, respectively, small molecules^29^ and polymer-based^30^ dynamic covalent amphiphiles, to dynamic covalent lipopolyplexes made of amphiphilic dynamic covalent polymers (Fig. 1).

## Results and discussion

### Design and synthesis

Hydrophobization can be achieved using aromatic amino acids (*e.g.* tryptophan, Trp), saturated fatty acids or more complex lipids. While introducing Trp was found advantageous in cell-penetrating peptides as revealed by the Sagan/Alves groups^31–33^ and others,^34^ it unfortunately did not prove superior when applied to our DCPs (data not shown). Then, inspired by the successful use of saturated fatty acids for making cationic and ionizable lipids for siRNA transfection by LNPs,^35,36^ we turned our attention to appending our cationic peptide-based building blocks with fatty acids positioned at their N-terminus, screening the effect of chain length and comparing with non-amphiphilic DCPs. Monomers made of three arginines were selected since they were previously found to best promote siRNA complexation and delivery.^16^

**Scheme 1:**
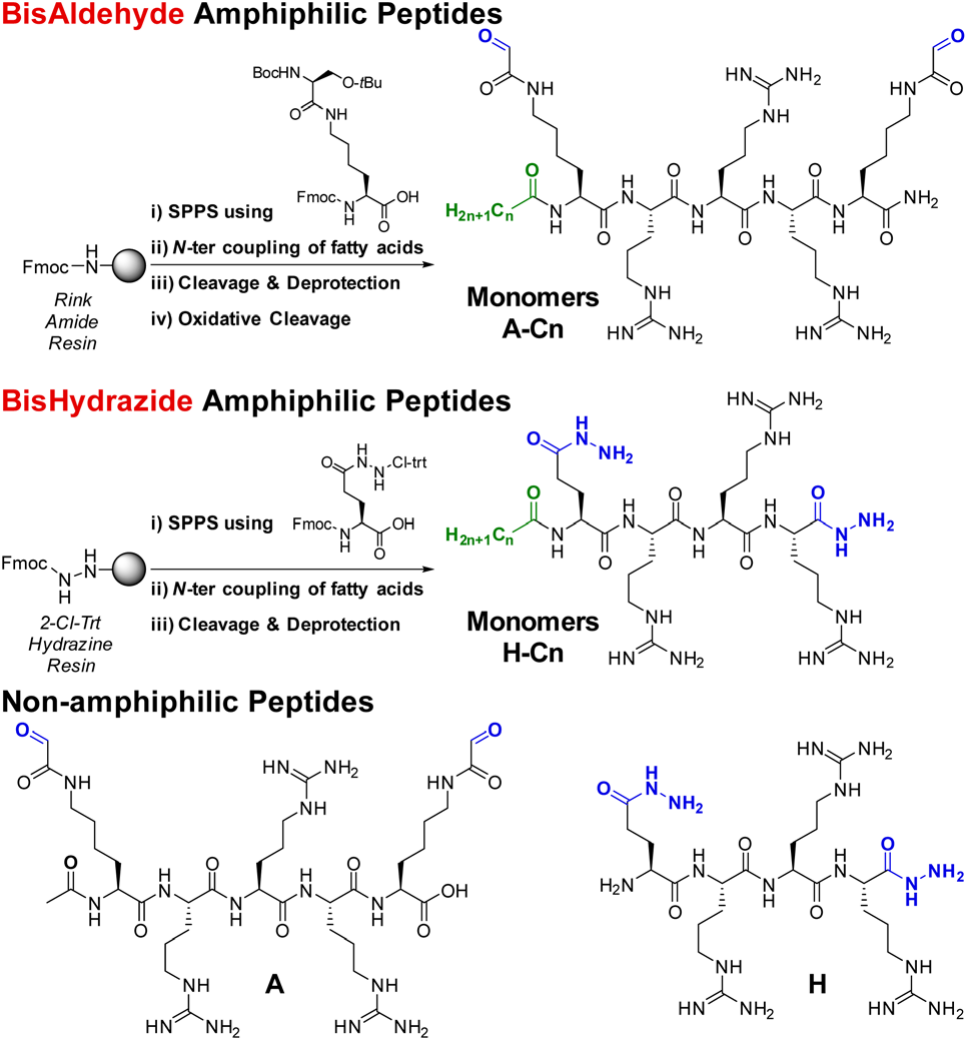
Synthetic route for the preparation of the amphiphilic cationic peptide monomers (bisaldehydes A-C*n*, top; bishydrazides H-C*n*, bottom), and non-amphiphilic building blocks A and H.

The non-amphiphilic cationic bisaldehyde peptide A was synthesized as previously described by solid phase peptide synthesis (SPPS). An additional coupling with fatty acids through amide-bond formation was performed directly on solid support at the end of the SPPS and yielded the desired amphiphiles A-C*n* (*n* = 6, 10, 12, 18) in 3–28% overall yields (Scheme 1).

The complementary bishydrazide peptides H and H-C*n* (*n* = 6, 10, 12, 18) were similarly obtained by SPPS, using a non-natural glutamic acid hydrazide amino acid and a modified 2-chlorotrityl hydrazine resin (see ESI†), in 2–30% overall yields (Scheme 1). All the amphiphilic peptides were nicely soluble in water up to 10 mM, except the C18 ones which were used for further studies from 10 mM stock solutions in DMSO.

**Scheme 2:**
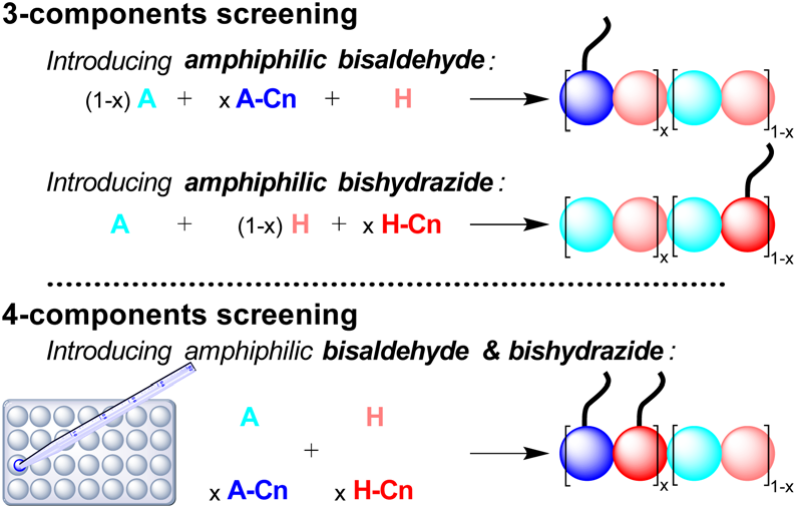
Description of the screening methodology where amphiphilic monomers are introduced either once at a time (3-components screening, top) or simultaneously (4-components screening, bottom). The colored spheres represent the different peptide-based monomers and the tail represents the aliphatic chain. The DCPs represented here are depicted, for simplicity, as block co-polymers but the amphiphilic monomers can also be statistically distributed within the DCPs. Instead of dynamic covalent co-polymers, it is also possible, in the case of the 4-components screening, to have selection and amplification of one DCP at the expense of other possibilities.

The multi-component screening involves either 3-components, introducing one amphiphilic monomer at a time, or 4-components where both amphiphilic monomers are introduced simultaneously in the same proportion (Scheme 2).

### SiRNA complexation

**Fig. 2:**
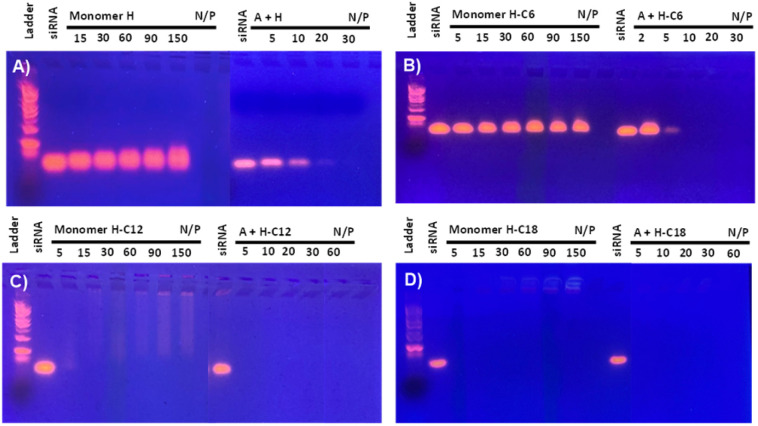
SiRNA complexation, assessed by electrophoretic gel retardation assay, of DCPs *versus* amphiphilic monomer H: (A) A–H, (B) A–H-C6, (C) A–H-C12, and (D) A–H-C18. Concentration of each cationic peptide monomer = 66–400 μM, depending on the N/P. Concentration of siRNA = 1.9 μM.

SiRNA complexation was assessed by an electrophoretic gel retardation assay to identify the optimal monomers composition, testing different N/P ratio. The activity of DCPs formed *in situ* through siRNA-templating was compared with the activity of the cationic bishydrazide components H and H-C*n* – the bisaldehyde component A alone was previously found unable to complex siRNA up to N/P 100.^16^ The cationic peptide monomers were incubated overnight in sodium acetate buffer (25 mM, pH 5.5) in the presence of a fixed amount of siRNA. The results reveal that the non-amphiphilic DCP A–H complexes siRNA at N/P 20, which is comparable to our previous generation, while the starting building block H remained inactive up to N/P 150 (Fig. 2(A)).^16^ Performing the 3-components screening by inserting the amphiphilic bishydrazides H-C*n* resulted in the similar behaviour for the DCP A–H-C6: complexation was found to be now complete at N/P 10 whereas H-C6 showed no sign of complexation up to N/P 150 (Fig. 2(B)). The enhancement of siRNA complexation of the amphiphilic DCP A–H-C6 compared to the hydrophilic DCP A–H indicates an assistance of hydrophobic interactions during DCP and polyplex which may come from the formation of hydrophobic pockets where the non-covalent interactions governing polyplex formation are strengthened. Finally, moving to the longer C12 and C18 amphiphilic DCPs revealed that hydrophobic interactions become dominant in driving the assembly, reaching full complexation at N/P = 5 for DCPs A–H-C12 and A–H-C18, but also for the amphiphilic components H-C12 and H-C18 (Fig. 2(C) and (D)).

**Fig. 3:**
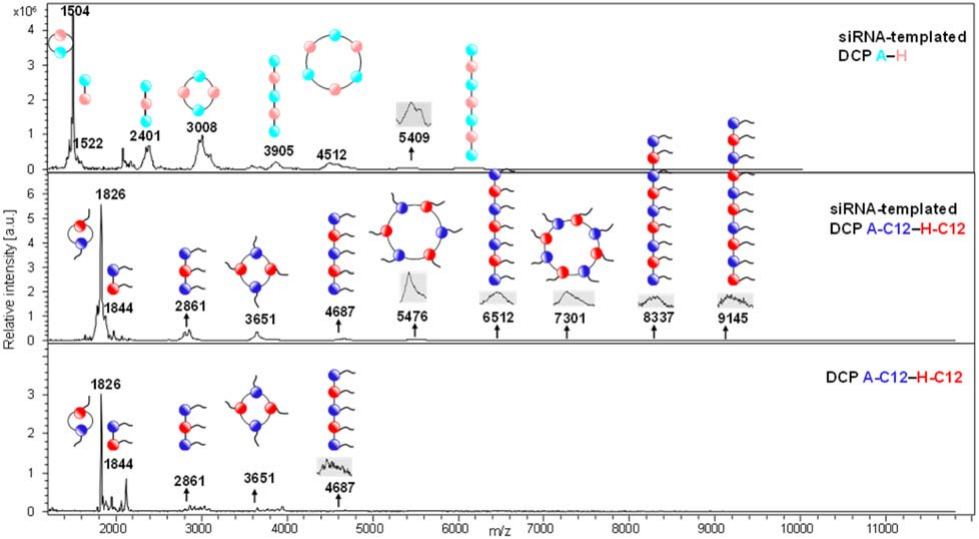
MALDI-TOF characterization of DCPs. Comparative analysis of siRNA-templated (A–H, top; A-C12–H-C12, middle) and non-templated DCPs (A-C12–H-C12, bottom). The coloured circles represent the cationic (A and H in light blue and light red, respectively) and amphiphilic (A-C12 and H-C12 in blue and red) peptide monomers.

The DCPs formed by A + H or A-C12 + H-C12 were characterized by native MALDI-TOF mass spectrometry directly on the polyplexes formed at N/P 5. The results show the formation of cyclic and linear oligomers up to a degree of polymerization DP = 7–10. Since the detectability of oligomers by mass spectrometry decreases with length, it is also likely that longer oligomers do form. However, this result is consistent with our previous prediction^17^ and with the findings of Aida and co-workers (Fig. 3, S33 and Table S1†).^21^ Interestingly, the data confirm (i) the assistance of aliphatic chains – longer DPs are obtained for the amphiphilic DCPs – and (ii) the templating effect of siRNA – shorter oligomers being formed in the absence of siRNA.

### SiRNA delivery

**Fig. 4:**
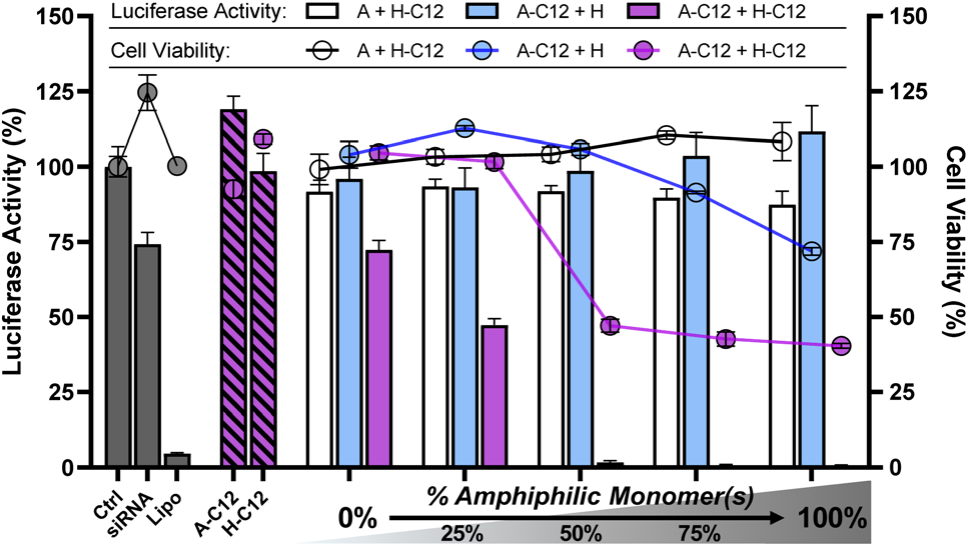
Luciferase activity knockdown and cell viability in MCF7-Luc cells of siRNA polyplexes formed from 3-components half-amphiphilic DCPs (A + (100 − *x*)% H + *x*% H-C12: white bars/circles), ((100 − *x*)% A + *x*% A-C12 + H: cyan bars/circles) and 4-components amphiphilic DCPs ((100 − *x*)% (A + H) + *x*% (A-C12 + H-C12): purple bars/circles), compared to siRNA alone (negative control), lipofectamine (positive control), A-C12 alone, and H-C12 alone. Luciferase activity was determined by luminescence measurments at 72 h after transfection. Data shows the mean ± S.E.M. of conditions performed in triplicates. The experiments with DCPs were carried out at N/P 20 and those with the monomers A-C12 and H-C12 alone were therefore performed at N/P 10. SiRNA dose: 100 nM.

Having demonstrated effective siRNA complexation, we then assessed the efficiency of these amphiphilic DCPs to deliver siRNA in cells by a luciferase activity knockdown assay in an MCF-7-Luc cancer cell line at a constant siRNA dose of 100 nM. The data reported here were normalized to the total number of living cells and thus reflect a true silencing activity (see ESI†). All experiments were initially performed at N/P 20 where siRNA complexation was found complete for all DCPs. Polyplexes were formulated at N/P 20 by incubating overnight a mixture of complementary monomers (100 μM each) with siRNA (0.72 μM) in sodium acetate buffer (25 mM, pH 5.5). In order to study the effect of hydrophobization on the silencing efficiency of siRNA, we performed a multi-component screening approach by introducing increasing proportions of amphiphilic monomers (*e.g.*A-C12 and H-C12, Fig. 4). The starting point (0% amphiphilic monomers) shows no silencing effect – DCP A–H being therefore an ineffective vector despite its ability to effectively complex siRNA (*vide supra*). However, albeit the amphiphilic monomers A-C12 and H-C12 showed no activity alone (Fig. 4, hatched bars), the corresponding 4-component screening of amphiphilic DCPs ((100 − *x*)% (A + H) + *x*% (A-C12 + H-C12)) reveals a dose-dependent inhibition of luciferase activity, reaching maximum efficacy for the DCPs A-C12–H-C12 (Fig. 4, purple bars). This is remarkable given that the 3-component screening of half-amphiphilic DCPs (*i.e.* (A + (100 − *x*)% H + *x*% H-C12) or ((100 − *x*)% A + *x*% A-C12 + H)) did not show activity at all (Fig. 4, white and cyan bars, respectively). In other words, this means that efficient silencing of luciferase requires the introduction of both amphiphilic monomers. The successful delivery of siRNA was further confirmed by fluorescence microscopy using an Atto-488-labelled siRNA (Fig. S34†).

The cell viability was assessed by a colorimetric analysis using (4,5-dimethylthiazol-2-yl)-2,5-diphenyltetrazolium bromide (MTT). While the monomers A-C12 and H-C12 alone showed no cytotoxicity, a significant decrease in cell viability to around 50% was observed when introducing more than 50% of amphiphilic monomers within the 4-component DCPs. Although the onset of cytotoxicity seems to be delayed compared to the silencing activity (see the 25% composition in Fig. 4), it is clear the two remain linked in this series of dynamic covalent lipopolyplexes. A LDH assay confirmed this trend (Fig. S35†), which suggests that the cytotoxicity of the dynamic covalent lipopolyplexes originate from a destabilisation of cellular membranes.

**Fig. 5:**
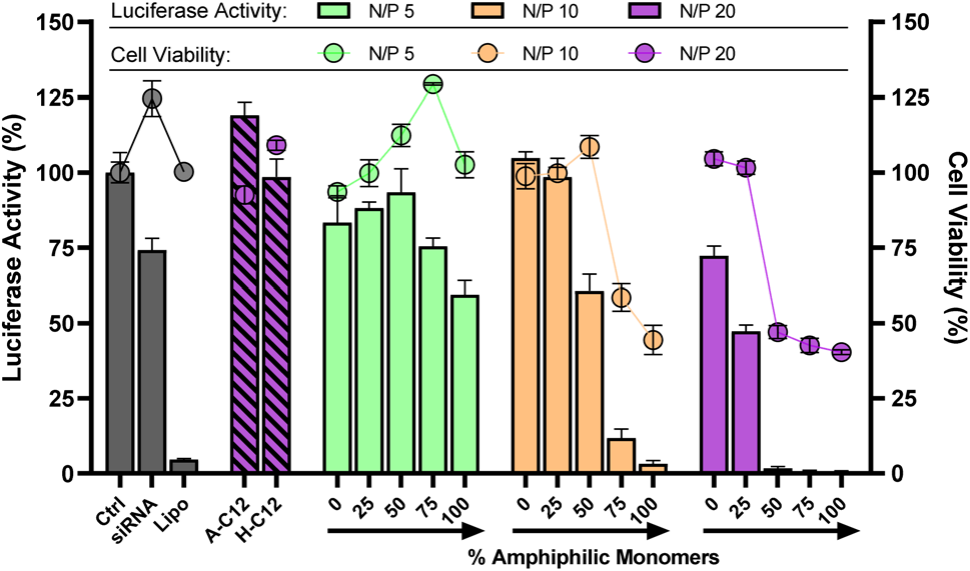
Luciferase activity knockdown and cell viability in MCF7-Luc cells of siRNA polyplexes formed from 4-components amphiphilic DCPs ((100 − *x*)% (A + H) + *x*% (A-C12 + H-C12)) at different N/P, compared to siRNA alone (negative control), lipofectamine (positive control), A-C12 alone, and H-C12 alone. Luminescence readout was performed 72 h after transfection. Data show the mean ± S.E.M. of conditions performed in triplicates. The experiments with the monomers A-C12 and H-C12 alone were performed at constant monomer : siRNA molar ratio, therefore at N/P 10. SiRNA dose: 100 nM.

Since the excess of positive charges can account for cytotoxicity,^37^ lower N/P were tested. In our case, both the cytotoxicity and the silencing activity were found to decrease at N/P 5 and 10 (Fig. 5). We also noticed that the insertion of more amphiphilic monomers is required when working at lower N/P. For instance, the onset of activity is observed at 25% amphiphilic monomers at N/P 20, but occurs at 50% of amphiphilic monomers at N/P 10 (Fig. 5). Similarly, testing lower siRNA doses (25 and 50 nM) showed the same trend but, for the first time, silencing activity was detected at a record low dose of 50 nM with DCP A-C12–H-C12 (Fig. S36†).

**Fig. 6:**
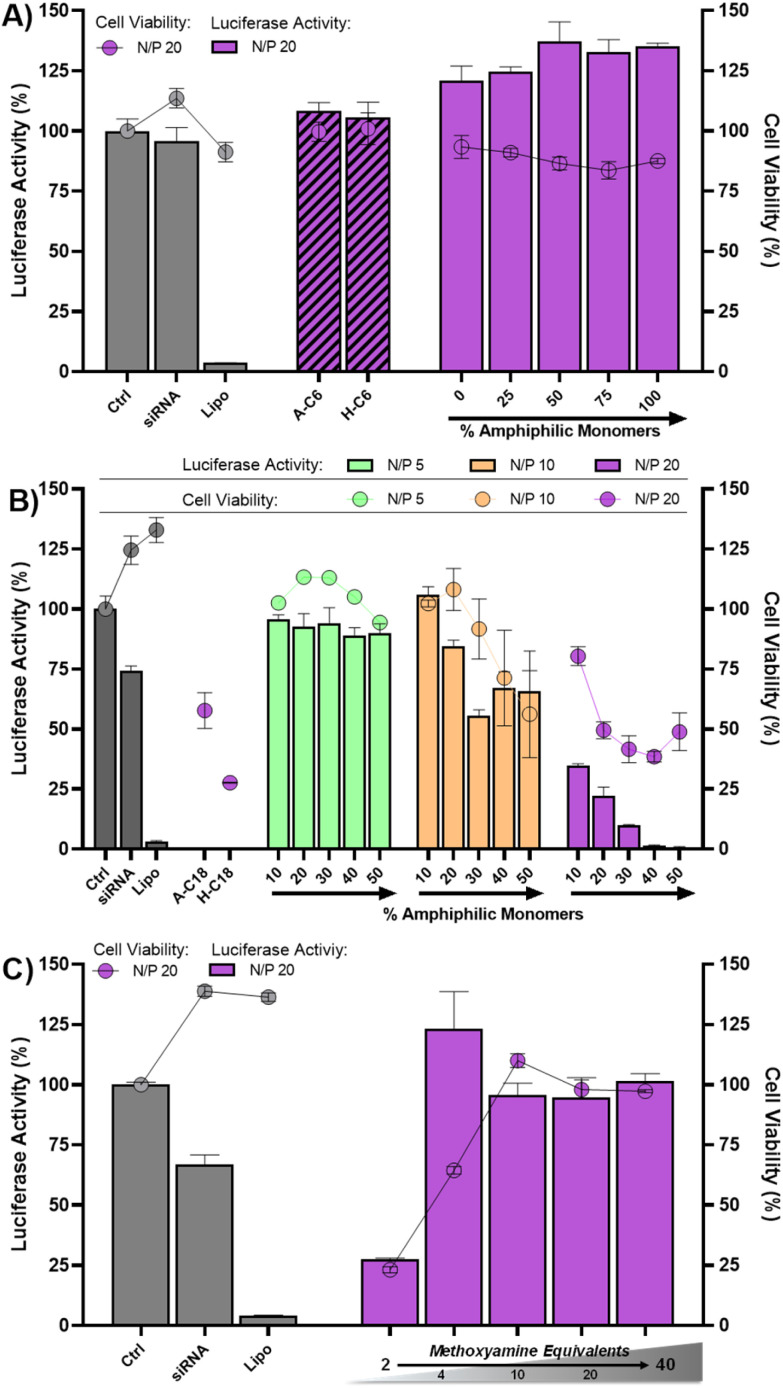
Luciferase activity knockdown and cell viability in MCF7-Luc cells of siRNA polyplexes formed from 4-components amphiphilic DCPs with: (A) A-C6 and H-C6; (B) A-C18 and H-C18; (C) A-C12 and H-C12 in the presence of the polymerization terminator methoxyamine (2 to 40 equivalents with respect to bisaldehyde A-C12). Luminescence readout was performed 72 h after transfection. Data shows the mean ± S.E.M. of conditions performed in triplicates. SiRNA dose: 100 nM.

We then tested the effect of chain length. The amphiphilic DCPs bearing the short C6 chain were found totally inactive (Fig. 6(A)). Even increasing the chain length to C10 did not elicit any siRNA delivery activity (Fig. S37†). On the other hand, those with the C18 chain were found very active. Although the introduction of only 10% of C18-bearing amphiphilic monomers was sufficient to elicit a potent silencing activity with low associated cytotoxicity, the toxicity displayed by the monomers A-C18 and H-C18 discarded the selection of C18-bearing amphiphilic DCPs for further development (Fig. 6(B)).

Finally, to check whether the formation of DCP A-C12–H-C12, involving multiple acylhydrazone bonds, is indeed the underlying cause promoting siRNA complexation and delivery, we repeated the luciferase knockdown experiment in the presence of methoxyamine as a polymerization terminator. Indeed, by reacting with the bisaldehyde component to form stable oxime products, the action of methoxyamine prevents complementary monomers to associate into DCPs.^10,38^ Interestingly, transfection was inhibited in the presence of methoxyamine, thus demonstrating that templated polymerization is required to trigger siRNA transfection and luciferase silencing (Fig. 6(C)).

### Physicochemical properties

The size of the polyplexes formed from the amphiphilic DCPs bearing the C12 chain was characterized by dynamic light scattering (DLS) and *ζ* potential measurements.

When combined with siRNA at N/P 10, none of the monomers formed any detectable object by DLS (data not shown). Large nanoparticles (>800 nm) were detected for the half-amphiphilic DCPs A-C12–H or A–H-C12 (Table 1, entries 1 and 2), which may explain their inability to deliver siRNA in cells. However, no nanoparticles were detected in the absence of siRNA, showing again the critical role of this templating component. Interestingly, the polyplexes constructed at N/P 20 from the amphiphilic DCPs, composed of both C12 monomers, show smaller nanoparticle sizes – between 170 and 400 nm – and a decreasing polydispersity index (PDI down to 0.08) as the percentage of amphiphilic monomers increased (Table 1, entries 3–7). A correlation with the percentage of amphiphilic monomers is also observed looking at the surface potential, with *ζ* potential values ranging from a minimum of −22 mV to a maximum of +26 mV (Table 1). This suggests that lipid moieties help the structuration of the assembly and promote positive charges on the surface of the assembly, which might also contribute to stabilize the particles by electrostatic repulsion.

**Table 1:** DLS analyses (size and polydispersity index) and *ζ* potential measurements of DCP-based polyplexes. Polyplexes formation was performed *in situ* by mixing and incubating overnight siRNA with complementary monomers (composition indicated, 100 μM for each monomer) in sodium acetate buffer (25 mM, pH 5.5). Further dilution in the same buffer to a final concentration of 100 nM siRNA was performed prior to the measurements

Entry	Composition	N/P	*x*% C12	Size (nm)	PDI	*ζ* potential (mV)
1	A-C12 + H	20	50	1536 ± 99	0.22	−22 ± 12
2	A + H-C12		50	805 ± 19	0.32	−10 ± 20
3	(100 − *x*)% (A + H) + *x*% (A-C12 + H-C12)	20	0	332 ± 126	0.27	−2 ± 5
4			25	171 ± 6	0.44	−2 ± 25
5			50	324 ± 54	0.32	−5 ± 11
6			75	407 ± 114	0.19	+1 ± 13
7			100	195 ± 47	0.08	+26 ± 9
8		10	0	n.d.	—	—
9			25	333 ± 88	0.39	—
10			50	255 ± 190	0.27	—
11			75	977 ± 60	0.54	—
12			100	760 ± 154	0.25	−2 ± 8
13		5	0	n.d.	—	—
14			25	n.d.	—	—
15			50	1198 ± 61	0.63	—
16			75	1055 ± 26	0.44	—
17			100	2293 ± 77	0.51	−14 ± 9

Decreasing the N/P ratio from 20 down to 10 and 5 has a major impact, with nanoparticles increasing in size, being more polydisperse, and having decreased *ζ* potential values (Table 1, entries 8–17). The study of the polyplexes formed from the DCPs bearing the C6 and C18 chains revealed that the former generated larger nanoparticles of *ca.* 700 nm while the latter display sizes of 200–525 nm but more positive *ζ* potential values of 15–56 mV (Table S6†).

**Fig. 7:**
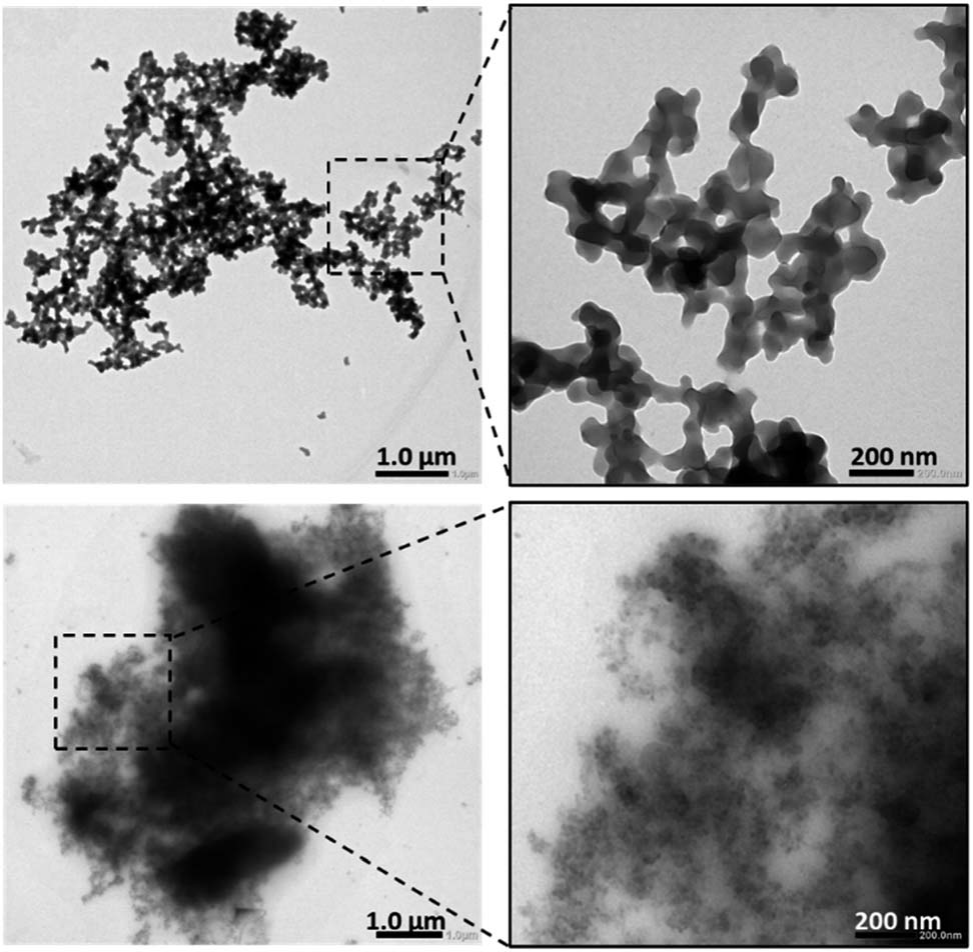
TEM images of the polyplex formed *in situ* from the DCP A-C12–H-C12 (top), and of the same system in the absence of siRNA (bottom). Dynamic covalent lipopolyplexes formation was performed overnight at a monomer concentration of 1 mM in sodium acetate buffer pH 5.5.

The nanoparticles formed from the DCP A-C12–H-C12 were further characterized by transmission electron microscopy (TEM). The polyplexes at N/P 20 were formed at a higher concentration to improve the signal and obtain sufficient contrast. The results show that these polyplexes appear as dense spherical nanoparticles cross-linked in larger aggregates (Fig. 7, top, and S38†), in stark contrast with the blurry and ill-defined structure observed with the exact same system but in the absence of siRNA (Fig. 7, bottom), thereby confirming again the prime role of the templating siRNA in directing the complex self-assembly pathway towards a more organized state. A particle size analysis revealed an average size for the individual spheres around 80 nm.

## Conclusions

We reported here the design and synthesis of new amphiphilic cationic peptide monomers for the siRNA-templated formation of DCPs and dynamic covalent lipopolyplexes. By screening multi-component libraries of amphiphilic DCPs, we successfully identified effective vectors of siRNA capable of eliciting a complete knockdown of luciferase at 100 nM dose, where our previous generation only achieved a 50% knockdown at 200 nM siRNA dose.^16,17^ Significant siRNA activity was even detected at a 50 nM dose which sets a new record for DCP-based vectors. Moreover, by introducing these amphiphilic monomers, we also found that the physico-chemical properties of the resulting dynamic covalent lipopolyplexes are improved with the best hit (DCP A-C12–H-C12) leading to more defined nanoparticles (*ca.* 200 nm, PDI = 0.08) compared to the previous generation (*ca.* 400 nm, PDI = 0.4).^16,17^ Overall, the results show that hydrophobization plays a major role in siRNA complexation, in the self-assembly of DCPs and dynamic covalent lipopolyplexes, and also boost the activity of siRNA delivery in cells, even though it comes with the critical challenge of dealing with associated cytotoxicity. This work contributes to the emerging exploration of (supra)molecular vectors which designs combine the best from lipoplexes and polyplexes.^39^ We believe the simplicity and versatility of our *in situ* screening methodology which operates in mild conditions (simple mixing, room temperature, aqueous media) will enable further exploration of adaptive and evolutive multi-components libraries from which, through the templating action of nucleic acids and cell membranes, effective vectors can be selected and identified.^14,40^

## Author contributions

Investigation (J. G. C., P. T., S. D. P., Y. B.); resources (Y. B., L. L.); visualization (J. G. C., S. U.); writing – original draft (J. G. C., S. U.); writing – review & editing (all); supervision/validation (E. S., N. B., S. U.); conceptualization/methodology/funding acquisition/project administration (J. L. C., S. U.).

## Conflicts of interest

There are no conflicts to declare.

## Supplementary Material

SC-016-D4SC07668K-s001

## Data Availability

The data supporting this article have been included as part of the ESI.†
