# Supplementary material for: Amphiphilic dynamic covalent polymer vectors of siRNA
Source: Chem Sci. 2024 Dec 30;16(5):2413–9. doi: 10.1039/d4sc07668k (PMC11707677; doi:10.1039/d4sc07668k)
Supplement: SC-016-D4SC07668K-s001 [file SC-016-D4SC07668K-s001.pdf]

Supplementary Information (SI) for Chemical Science.
This journal is © The Royal Society of Chemistry 2024

SUPPORTING INFORMATION

1. General procedures and materials 2
2. Synthesis of Fmoc-*L*-Glu[NHNH(2Cl-Trt)]-OH 6
3. Synthesis of Bisaldehyde Peptides A-Cn....................9
   a) **Compound A-C6** 9
   b) **Compound A-C10** 13
   c) **Compound A-C12** 17
   d) **Compound A-C18** 21
4. Synthesis of Bishydrazide Peptides H and H-Cn 26
   a) **Compound H**....................26
   b) **Compound H-C6** 29
   c) **Compound H-C10** 30
   d) **Compound H-C12** 32
   e) **Compound H-C18** 35
5. MALDI-TOF mass spectrometry 37
6. SiRNA delivery 39
   a) **Fluorescence imaging**....................39
   b) **LDH assay** 39
   c) **Dose dependent C12-DCP activity**....................40
   d) **4-component C10-DCP activity**....................40
7. DLS & ζ Potential 41
8. TEM 44
9. References....................44

1. General procedures and materials

All reagents and solvents were obtained from commercial sources and used without further purification. The Fmoc-protected hydrazine resin (0.8 mmol/g) was prepared from the 2-chlorotrityl chloride resin (loading; 1.60 mmol Cl/g) as previously described.[1,2] The bisaldehyde peptide **A** was synthesized as previously described.[3]

The different siRNA sequences are, for anti-firefly luciferase (siLuc): 5′ CUUACGCUGAGUACUUCGAdTdT-3’ (sense strand), and 5’-UCGAAGUACUCAGCGUAAdTdT-3’ (anti-sense strand), for siRNA without biological activity (siCtrl): 5’-CGUACGCGGAAUACUUCGAdTdT-3’ (sense strand) and 5’-UCGAAGUAUUCCGCGUACG dTdT-3’ (anti-sense strand), and for epifluorescence imaging (Atto-488-siCtrl): 5’-CGUACGCGGAAUACUUCGAdTdT-3’ (sense strand) and 5’-UCGAAGUAUUCCGCGUACGdTdT- 3’ (anti-sense strand) were purchased from Eurogentec (Serring, Belgium). Lipofectamine RNAiMAX was purchased from Invitrogen (Cergy Pontoise, France). Cell viability reagent 3-(4,5-dimethylthiazol-2-yl)-2,5-diphenyltetrazolium bromide (MTT) was purchased from Sigma-Aldrich (Saint-Quentin-Fallavier, France).

Nuclear magnetic resonance spectroscopy (NMR). ^{1}H and ^{13}C NMR spectra were recorded at 400 MHz for ^{1}H and 100 MHz for ^{13}C (Bruker Avance 400 instruments) in deuterated solvents. Peaks were referenced in ppm with respect to the residual solvent peak. Data are reported as follows: chemical shift (δ in ppm), multiplicity (s for singlet, d for doublet, t for triplet, m for multiplet), coupling constant (*J* in Hertz), and integration.

High-performance liquid chromatography (HPLC). Analytical reverse-phase HPLC (RP-HPLC) analyses were performed on a Thermo Scientific™ - UltiMate™ 3000 UHPLC system equipped with a Thermo Scientific™ Hypersil GOLD™ aQ C18 Polar Endcapped HPLC Column 25302-052130, (1.9 µm, 2.1 x 50 mm) column and a Thermo Scientific™ Dionex™ UltiMate™ DAD 3000 detector using the following linear gradients (Solution A: 99.9% Water, 0.1% TFA; Solution B: 99.9% Acetonitrile, 0.1% TFA). Retention times (t_R) are given in minutes.

i) Method **[HPLC 1]:** 95% A (5% B) to 0% A (100% B) in 5 minutes, then up to 10 min at 100% B; flow: 0.5 mL/min.
ii) Method **[HPLC 2]:** 100% A (0% B) to 0% A (100% B) in 5 minutes, then up to 10 min at 100% B; flow: 0.5 mL/min.

Preparative HPLC (Prep-HPLC) was performed on a Gilson® PLC 2250 Purification System equipped with a UV-Vis Glison® DAD detector and using the following solutions (Solution A: 99.9% Water, 0.1% TFA; Solution B: 99.9% Acetonitrile, 0.1% TFA). The purification system was equipped with a:

i) **[Prep-HPLC]** WATERS™ XSelect™ CSH C18 OBD Prep Column 186005493, (130Å, 5 µm, 30 nm X 250 nm)
ii) **[Semiprep-HPLC]** Macherey-Nagel® VP HPLC Column 250/10 NUCLEODUR C18 Htec 762566.100 (110Å, 7 µm, 10 nm X 250 nm).

Liquid chromatography-mass spectrometry (LC/MS). Analyses were performed on a

Shimadzu LCMS2020 (Phenomex Kenetex C18, 2.6 μm x 7.5 cm, 100Å) equipped with a SPD-M20A detector with the following linear gradient of solvent B (99.9% acetonitrile, 0.1% HCOOH) and solvent A (99.9% water and 0.1% HCOOH): 5 to 95% of solvent B in 5 min; flow 1 ml/min. Retention times (t_R) are given in minutes.

ESI mass spectrometry (ESI-MS). Analyses were carried out at the Laboratoire de Mesures Physiques, IBMM, Université de Montpellier using Micromass Q-Tof instruments.

MALDI-TOF mass spectrometry. The DCPs were analyzed by the Matrix-assisted laser desorption ionization-time of flight (MALDI-TOF) carried using Bruker UltrafleXtreme (smartbeam Nd: YAG laser source model). The mode of analysis of MALDI TOF is positive, linear mode, 20 kV acceleration voltage, and an acquisition mass range of 1000–10000 *m/z*. The radiation source was a pulsed nitrogen laser with a wavelength of 355 nm. The software for data acquisition and data analysis were flex control and flex analysis, respectively. α-cyano-4-hydroxy cinnamic acid (CHCA) was used as the matrix, at 10mg/mL, in H_2O/acetonitrile, containing 0.1% trifluoroacetic acid (TFA) and the peptide mixture pepmix 4 from LaserBioLabs was used as reference for spectrum calibration. The DCPs or siRNA complexes were mixed with the matrix in equal ratio (v/v) and applied on the MALDI-TOF MS target steel plate. All samples were prepared at N/P 5 at a 0.4 mM concentration of monomers in Ammonium Bicarbonate Buffer (A.B) at pH 5.5. After solvent evaporation, the sample spots were analyzed using 20,000 laser shots at a frequency of 200 Hz.

Solid Phase Peptide Synthesis (SPPS). All peptide syntheses were based on a Fmoc strategy and were carried out manually at room temperature. The following conditions for peptide couplings were used:

-Resin deprotection (Fmoc removal): piperidine/DMF (2/8) at r.t for 2 min (twice).

-Coupling conditions: Fmoc-AA-OH 0.6 M in DMF (5 eq.), HATU 0.2 M in DMF (5 eq.), DIEA (10 eq.), stirred at r.t. for 10 min. Double coupling was used except for the modified amino acid Fmoc-*L*-Glu[$NHNH_2$(2Cl-Trt)]-OH.

-Fmoc deprotection conditions: piperidine/DMF (2/8) at r.t. for 5 min (twice).

-Cleavage conditions:

[SPPS 1]

- Mild cleavage conditions: (TFA/CH_2Cl_2 (1/99) for 5 minutes, 4 times, then MeOH/Pyridine (8/2)) were used in the synthesis of **hydrophilic peptides** to cleave the peptide without deprotecting the amino acid side chains (Note that under this conditions Cl-Trt protecting groups are also deprotected) in order to facilitate purification by RP-HPLC.
- Deprotection conditions after mild cleavage: TFA/TIS/H_2O (95/2.5/2.5) solution at 3 mM concentration of protected peptide for 3 hours at room temperature.

[SPPS 2]

- Harsh cleavage/deprotection conditions: TFA/TIS/H_2O (95/2.5/2.5) solution for 3 hours at room temperature.

The final peptides were all titrated by ^{1}H NMR (D_2O or MeOD) using *tert*-butanol (in D_2O) or toluene (in MeOD) as internal reference to determine the exact concentration. For this, the compound was solubilized in D_2O or MeOD (final concentration around 30 mM) and *tert*-butyl alcohol or toluene was added (50 µL of a 30 mM solution in the corresponding deuterated solvent) in the NMR tube (total volume of 500 µL). ^{1}H NMR was recorded, and the relative peak integration was used to calculate the exact concentration of the compound.

Gel retardation assay. In sodium acetate buffer (25 mM, pH 5.5), a fixed concentration (1.9 µM) of siCtrl was mixed with the appropriate amounts of monomers to reach the different N/P ratios for a final volume of 20 µL. After 16 hours incubation time, 5 µL of blue 6X loading dye (Fisher Scientific) were added to the mixture. Electrophoresis was carried out on a 2 % w/v agarose gel mixed with GelRed™ nucleic acid gel stain (Interchim, France) in 1X TBE buffer (90 mM Tris-borate/2 mM EDTA, pH 8.2). The gel was run in 0.5X TBE at 100 V for 20 minutes. A 100 bp DNA ladder from Sigma-Aldrich (Saint-Quentin-Fallavier, S4 France) was used as a reference for the gel. The GelRed-stained siRNA was visualized using a TFX-20 M model-UV transilluminator (Vilber Lourmat, Marne-la-Vallée, France) and gel photographs were obtained with a smartphone camera.

Dynamic light scattering (DLS) and ζ-potential measurements. In sodium acetate buffer (25 mM, pH 5.5), siRNA complexes were prepared using different concentrations of siCtrl depending on the desired N/P (N/P 20: 0.72 µM; N/P 10: 1.44 µM; N/P 5: 2.88 µM); and at a fixed concentration of monomers (0.1 mM). Samples were diluted down to 100 nM siRNA before analysis using the same buffer. Measurements were performed using Zetasizer Nano-ZS instrument (Malvern, United Kingdom) with transparent ZEN0040 disposable micro-cuvette (40 µL) at 25°C. The same samples were used for ζ-potential measurements, which were performed using Zetasizer Nano-ZS instrument and DTS 1070 zeta potential cells at 25°C.

Transmission electron microscopy (TEM). TEM images were obtained by using a JEM 1400+ electron microscopy, at Microscopie Electronique et Analytique (MEA), Université de Montpellier. In sodium acetate buffer (25 mM, pH 5.5), siRNA complexes were prepared using different concentrations of siCtrl depending on the desired N/P (N/P 20: 7.2 µM; N/P 10: 14.4 µM; N/P 5: 28.8 µM); and at a fixed concentration of monomers (1 mM). 20 µL of the sample was dropped on a carbon coated copper grid and dried at room temperature, then the samples were observed at a 120 kV acceleration voltage at 25 °C.

Cell culture. Human Breast cancer (MCF-7, ATCC® HTB-22™) cell line was purchased from ATCC (Manassas, Virginia, USA). The MCF-7-Luc cell line derived from MCF-7 human breast cancer cells by stable transfection of firefly luciferase gene (PCDNA 3.1 CMV-Luc-SVNeo) was generously provided by Dr. P. Balaguer (ICM Montpellier, France). Selection of resistant clones was performed by geneticin addition at 1 $mg.mL^{-1}$ in free phenol red medium until experiments. MCF-7 cells were grown in F12/Dulbecco's modified Eagle's medium (DMEM) supplemented with 10% FVS , 100 $U.mL^{-1}$ penicillin and 100 $µg.mL^{-1}$ streptomycin. All cell lines were incubated at 37°C in a humidified atmosphere with 5% CO_2.

Cell luciferase assay. MCF-7-Luc were seeded at a density of 20.000 cells per well in 96-well white opaque tissue culture plates in 200 µL of their corresponding medium. Twenty-four hours after, the medium was exchanged by serum free fresh medium, and the appropriate amount of polyplex was added to reach 200 µL. Samples were prepared in sodium acetate buffer (25 mM, pH 5.5), using different concentrations of siLuc depending on the desired N/P (N/P 20: 0.72 µM; N/P 10: 1.44 µM; N/P 5: 2.88 µM); and at a fixed concentration of monomers (0.1 mM of bisaldehyde(s) + 0.1 mM of bishydrazide(s)). Thereafter, serum was added to achieve a final serum concentration of 4%. Three days after transfection (72 h), medium was replaced by fresh medium (100 µL) and then the expression of luciferase was assessed by addition of luciferin (10^{-3} M, final concentration) into culture medium. After 5 min, living cell luminescence was measured using a Varioskan™ LUX multimode microplate reader at 562 nm and were averaged from triplicates. The percentage of luminescence of treated cells was calculated by using the control cell as 100%. Luciferase activity was normalized in accordance with the total number of living cells in each sample as determined by the MTT assay as described below.

Cell viability (MTT) assay. MTT (4,5-dimethylthiazol-2-yl)-2,5-diphenyltetrazolium bromide) assay was performed to evaluate the cell viability.[5] Briefly, 20.000 cells were seeded into a 96 multi-well plate in 200 µL complete culture medium. Twenty-four hours after seeding, cells were treated with DCP/siLuc for 72 h as described in the section of “cell luciferase assay”. Cells treated with the vehicle were considered as a control. After this incubation, cells were treated for 4 h with 0.5 mg.mL^{-1} of MTT in media. The MTT/media solution was then removed, and the precipitated crystals were dissolved in EtOH/DMSO (1:1). The solution absorbance was read at 540 nm. The percentage of viable cells was calculated according to the following equation: %viability = $A_{measured}$/ $A_{control}$*100.

LDH assay. LDH (Lactate dehydrogenase) colorimetric assay was performed to evaluate cell cytotoxicity using CyQUANT™ LDH Cytotoxicity Assay Kit (ThermoFisher) and following the protocol recommended by the supplier. Briefly, 20.000 cells were seeded into a 96 multi-well plate in 200 µL complete culture medium. Twenty-four hours after seeding, cells were treated with DCP/siLuc for 72 h as described in the section of “cell luciferase assay”. Cells treated with 20 µL of ultrapure water were considered as a control (Spontaneous LDH activity), and cells treated with 20 µL of 10X “Lysis Buffer” for 45 minutes were considered as total cell death (Maximum LDH activity). After this incubation, 50 µL of each sample medium were transferred to a 96 multi-well flat bottom plate. Then, media was treated for 30 minutes with 50 µL of “Reaction Mixture”, followed by the addition of 50 µL of “Stop solution”. The solution absorbance was read at 490 nm and 680 nm. To determine LDH activity, the 680 nm absorbance value (background) was subtracted from the 490 nm absorbance before calculation of % cytotoxicity. The percentage of cytotoxicity was calculated according to the following equation: % Cytotoxicity = ([Compound-treated LDH activity – Spontaneous LDH activity] / [Maximum LDH activity – Spontaneous LDH activity]) *100.

Fluorescent Microscopy. EVOS microscope. Micrographs were captured using an EVOS M5000 Imaging System (ThermoFisher Scientific, Waltham, MA, USA). Analysis was performed using ImageJ 2.0 software.

2. Synthesis of Fmoc-*L*-Glu[NHNH(2Cl-Trt)]-OH

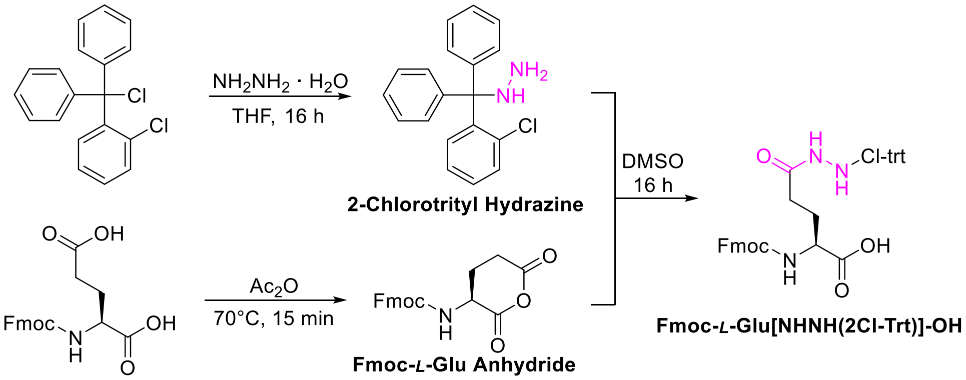

Scheme 1: Synthetic route for the preparation of Fmoc-*L*-Glu[NHNH(2Cl-Trt)]-OH.

2-Chlorotrityl Hydrazine. 2-Chlorotrityl Hydrazine was synthesized following the cited literature.[4]

Fmoc-*L*-Glu Anhydride. The compound was synthetized from an adapted literature procedure,[4] only decreasing the reaction temperature from 110ºC down to 70ºC to avoid

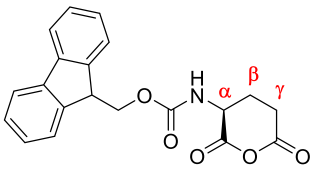

epimerization of the amino acid (Scheme 1). In a round bottomed flask, Fmoc-*L*-Glutamic acid (10.0 g, 26.5 mmol) was mixed with acetic anhydride (30 mL) to give a white suspension. Then the flask was stirred and heated at 70°C until a clear solution was obtained (15 min). The reaction mixture was then cooled to room temperature and then concentrated under vacuum. The residue obtained was washed with diethyl ether (2 x 10 mL) and dried under vacuum to yield Fmoc-*L*-Glu anhydride (9.45 g, 95%) as a white solid. **NMR ^{1}H** (DMSO-d6) δ_H: 7.92 (d, ^3J = 8.3, 1H, NH), 7.88 (d, ^3J = 7.5, 2H, Fmoc), 7.69 (d, ^3J = 7.5, 2H, Fmoc), 7.41 (t, ^3J = 7.4, 2H, Fmoc), 7.33 (t, ^3J = 7.4, 2H, Fmoc), 4.56 (ddd, ^3J = 13.4, ^3J = 7.8, ^3J = 5.7, 1H, α), 4.35 (m, 2H, Fmoc-CH_2), 4.24 (t, ^3J = 6.4, 1H, Fmoc-CH), 3.04 – 2.90 (m, 1H, γ-CH_2), 2.88 – 2.75 (m, 1H, γ-CH_2'), 2.15 – 1.98 (m, 1H, β-CH_2), 1.98 – 1.85 (m, 1H, β-CH_2').

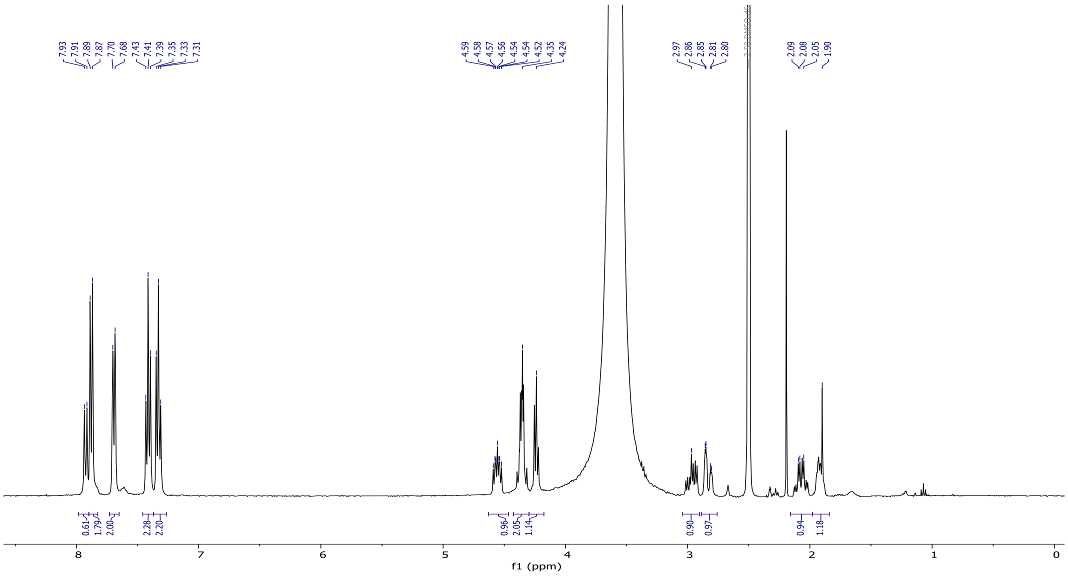

Figure 1. ^{1}H NMR (DMSO-d$_6$) spectrum of Fmoc-*L*-Glu Anhydride**.**

Fmoc-*L*-Glu[NHNH(2Cl-Trt)]-OH. The compound was synthetized from an adapted literature procedure,[4] changing the purification methodology, now enabling scale-up of the synthesis up to 10 g. Fmoc-*L*-Glu Anhydride (6.8 g, 19.2 mmol) and 2-chlorotrityl hydrazine (8.92 g, 28.8 mmol, 1.5 eq.) were loaded in a round bottomed flask. DMSO was then added (14 ml), and the mixture was stirred until complete dissolution. Then, the reaction mixture was stirred at room temperature for 2 h. Next, 150 mL of DCM were added, and organic layer was washed first with a 1M HCl solution (3 x 100 ml), followed by a saturated NaCl solution (3 x 1000 ml). The organic phase was then dried over Na_2SO_4 and concentrated under vacuum affording a viscous yellowish oil. The residue was dissolved in 100 mL of AcOEt in a large flask then, ice-cold petroleum ether (100 mL) was slowly added while stirring. A white powder started to precipitate, and the mixture was left to precipitate at -18°C overnight. Then, a white solid was collected by filtration and dried under vacuum to afford Fmoc-*L*-Glu[NHNH(2Cl-Trt)]-OH as a white powder (9.6 g, 75%). **LC-MS** t_R: 5.00 min. m/z calcd for $[C_{39}H_{34}ClN_3O_5+Na]^+$ 682.21, found 682.15; $[2C_{39}H_{34}ClN_3O_5+H]^+$ 1319.44, found 1319.40; $[2C_{39}H_{34}ClN_3O_5+Na]^+$ 1341.43, found 1341.35. Peak assignment and determination of the regiochemistry of the reaction was performed using 2D NOESY NMR (Figure 4). **^{1}H NMR** (DMSO-d_6) δ_H: 9.09 (d, ^3J = 8.4, 1H, NH), 7.88 (d, ^3J = 7.5, 2H, Fmoc), 7.79 (d, ^3J = 7.5, 1H, 2Cl-Trt), 7.69 (d, ^3J = 7.5, 2H, Fmoc), 7.48 – 7.12 (m, 4H, Fmoc) (m, 13H, 2Cl-Trt), 6.10 (d, ^3J = 8.2, 1H, NH’), 4.23 (m, 3H, Fmoc-CH_2 & Fmoc-CH), 3.73 – 3.60 (m, 1H, α-CH), 1.82 (t, ^3J = 7.9, 2H, γ-CH_2), 1.62 – 1.47 (m, 1H, β-CH_2), 1.47 – 1.35 (m, 1H, β-CH_2’).

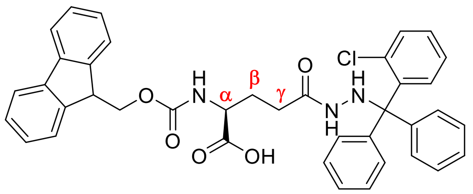

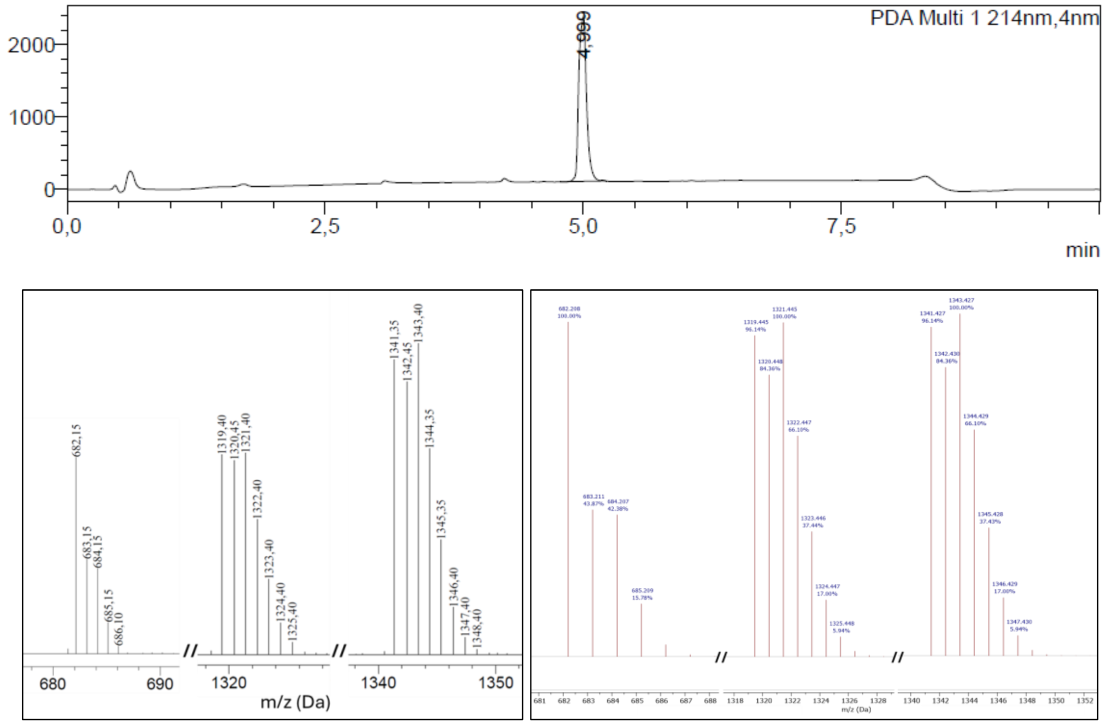

Figure 2. HPLC chromatogram (top), and experimental (bottom left) and theoretical (bottom right) MS spectra of Fmoc-*L*-Glu[NHNH(2Cl-Trt)]-OH.

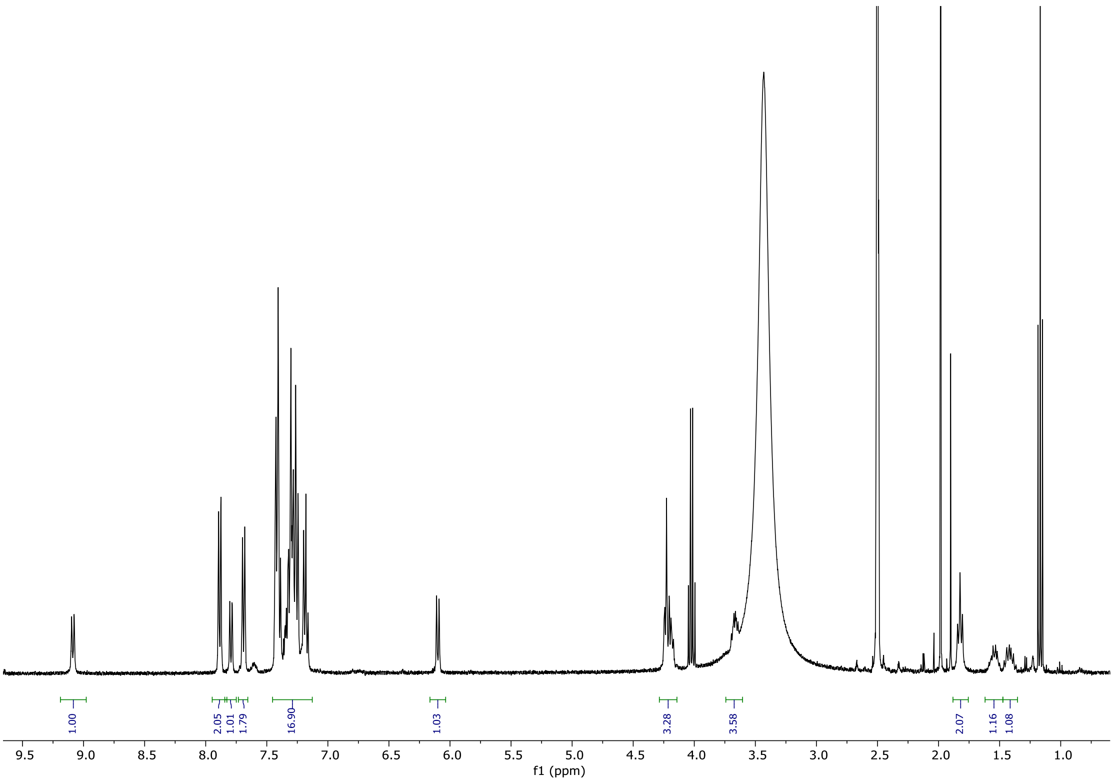

Figure 3. ^{1}H NMR (DMSO-d6) spectrum of Fmoc-*L*-Glu[NHNH(2Cl-Trt)]-OH.

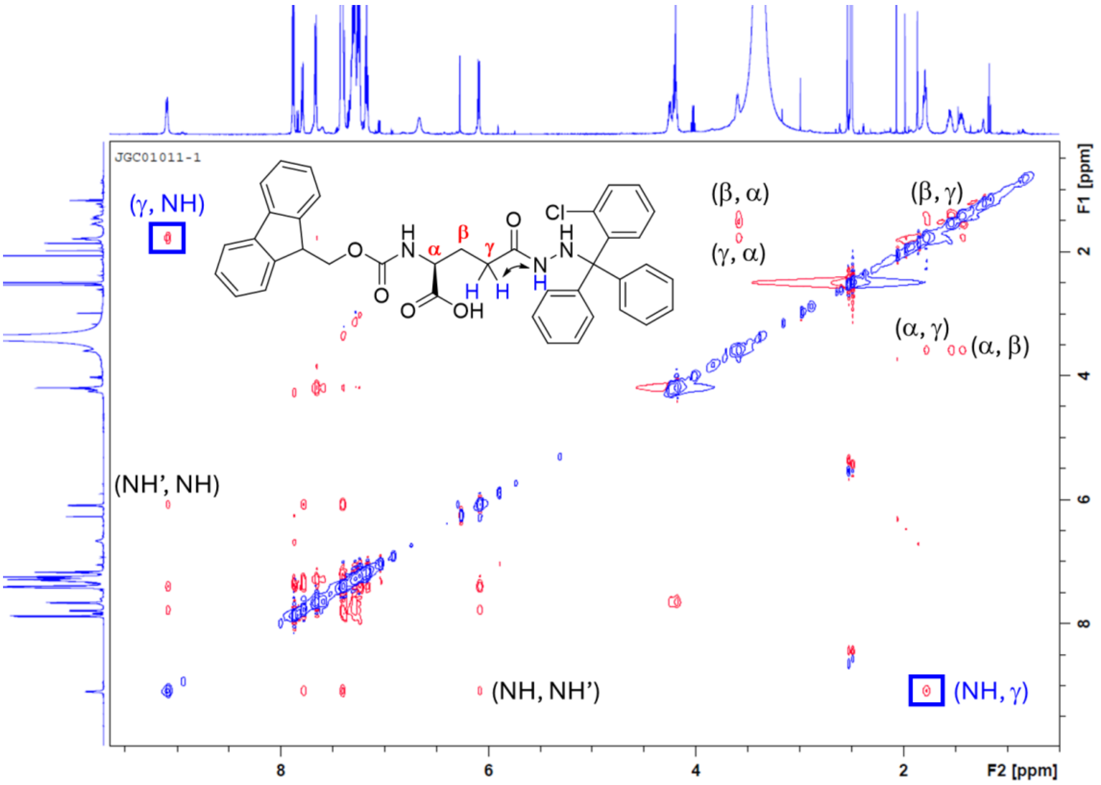

Figure 4. ^{1}H NOESY NMR spectrum (400 MHz, DMSO-d6) of Fmoc-*L*-Glu[NHNH(2Cl-Trt)]-OH. The correlation between α and NH protons proves the obtention of the right regio-isomer.

3. Synthesis of Bisaldehyde Peptides A-Cn

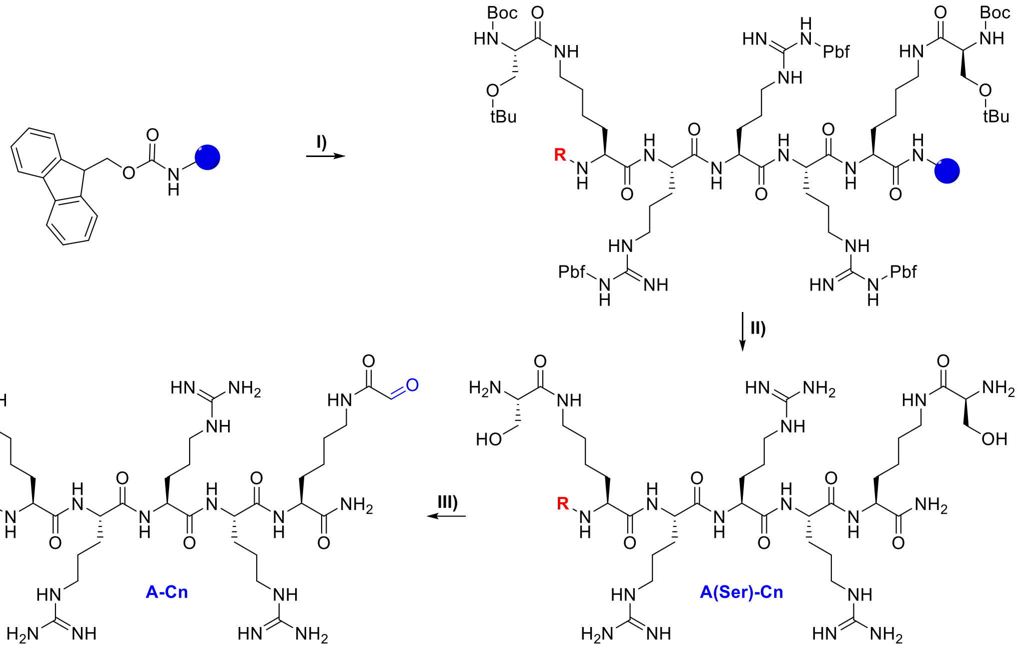

Scheme 2: Synthetic scheme for the preparation of the bisaldehyde amphiphilic peptides **A-Cn**. I) SPPS, II) cleavage and deprotection, III) oxidative cleavage of serine residues.

a) Compound A-C6

Compound A(Ser)-C6. The peptide was synthesized according to the general **[SPPS 2]** procedure using Fmoc-*L*-Arg(Pbf)-OH, Fmoc-*L*-Lys[Boc-*L*-Ser(O*t*Bu)]-OH and hexanoic acid.

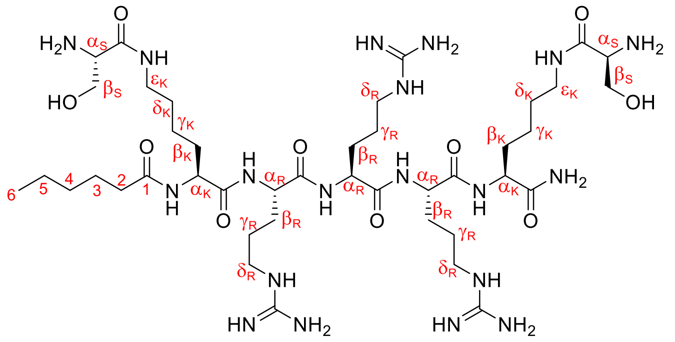

The solution was then concentrated, precipitated by adding ice cold Et_2O, and centrifuged. The supernatant was removed, and the crude material was freeze-dried. The compound **A(Ser)-C6** was obtained after preparative **[Prep-HPLC]** purification (gradient: 0 min, 100% A, 0% B; 5 min, 100% A, 0% B; 45 min, 0% A, 100% B). 220 mg were obtained (59%). **[HPLC 2]** t_R: 2.96 min. **HR-ESI-MS** m/z calcd for $[C_{42}H_{83}N_{19}O_{10}+2H]^{2+}$ 507.8385, found 507.8378; $[C_{42}H_{83}N_{19}O_{10}+3H]^{3+}$ 338.8930, found 338.8937. **^{1}H NMR** (D_2O) δ_H: 4.39 – 4.29 (m, 3H, $H\alpha_R$), 4.29 – 4.17 (m, 2H, $H\alpha_K$), 4.13 – 4.04 (m, 2H, $H\alpha_S$), 4.03 – 3.86 (m, 4H, $H\beta_S$), 3.33 – 3.15 (m, 6H, $H\delta_R$) (m, 4H, $H\varepsilon_K$), 2.29 (td, ^3J = 7.2, ^4J = 2.0, 2H, CH_2-CO(H2)), 1.94 – 1.19 (m, 4H, $H\beta_K$) (m, 4H, $H\gamma_K$) (m, 4H, $H\delta_K$) (m, 6H, Hb_R) (m, 6H, $H\gamma_R$) (m, 6H, CH_2(H3-H5)), 0.87 (t, ^3J = 6.9, 3H, CH_3(H6)).

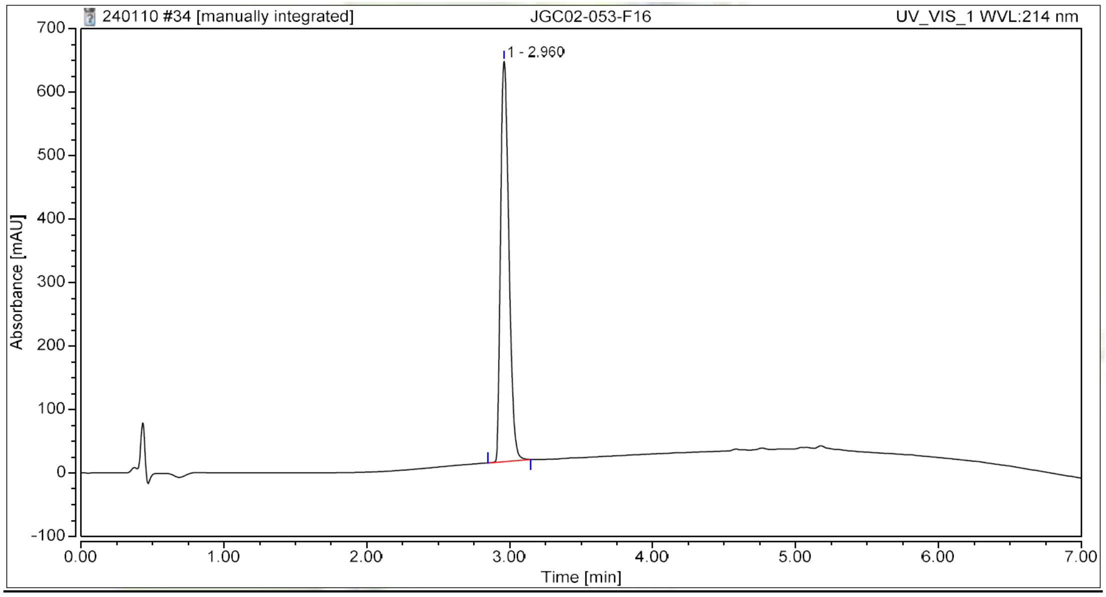

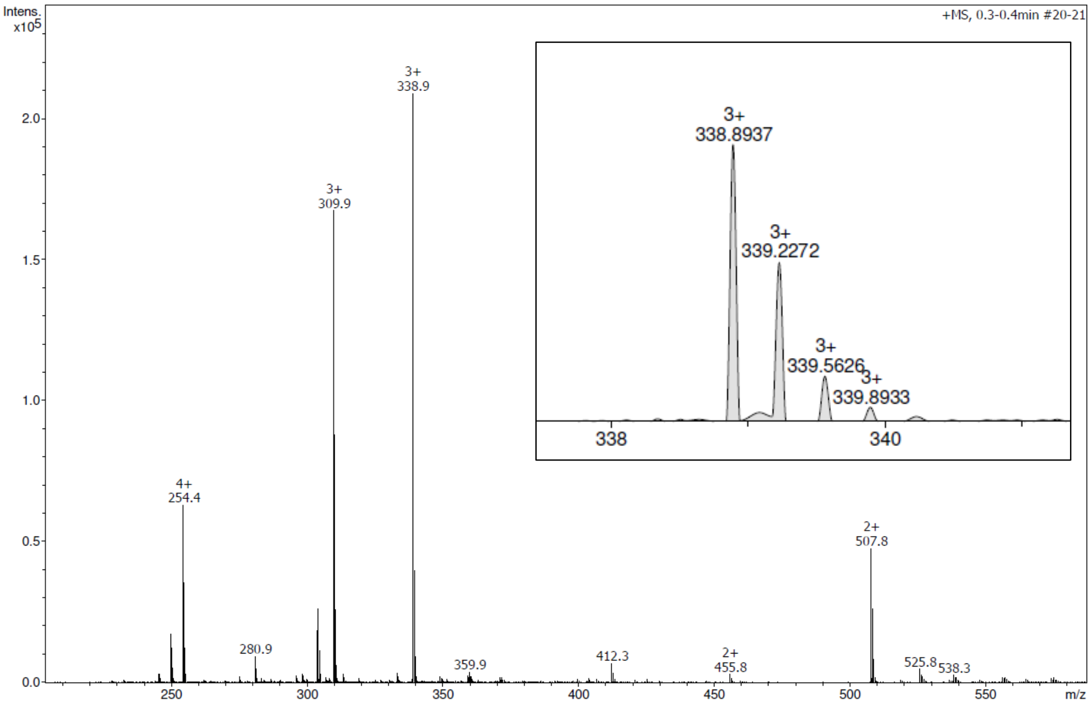

Figure 5. HPLC chromatogram (top) and HR-ESI-MS spectra (bottom) of compound **A(Ser)-C6**.

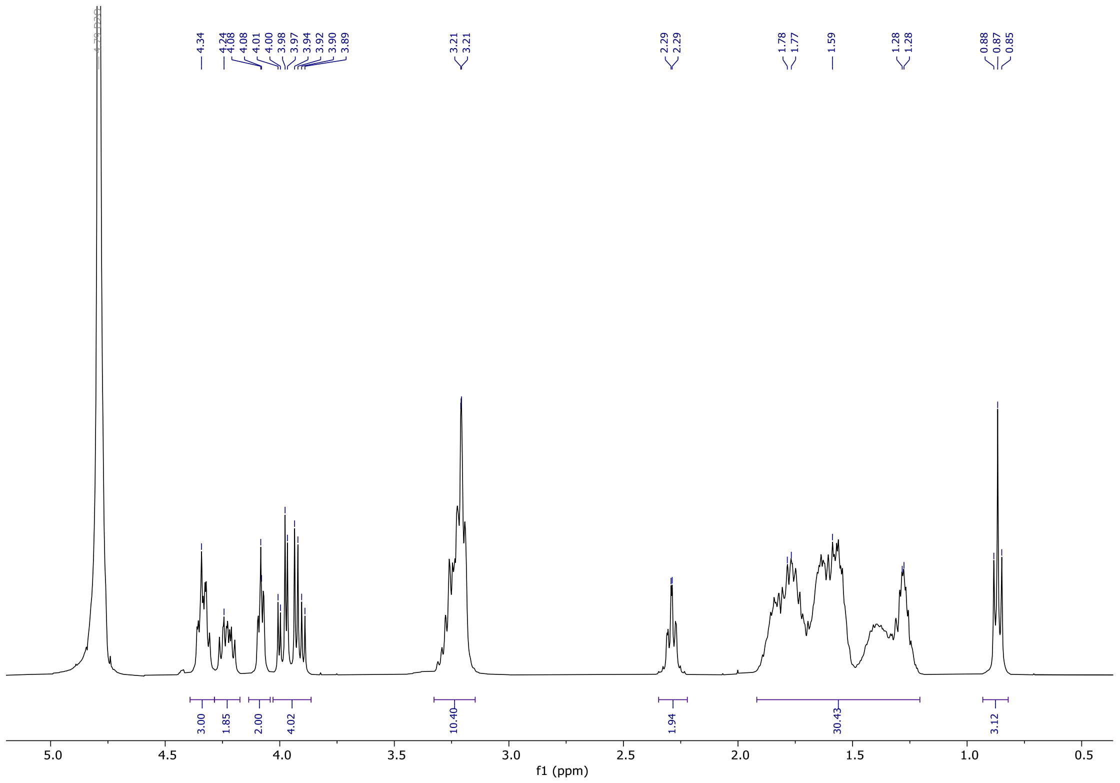

Figure 6. ^{1}H NMR (D_2O) spectrum of compound **A(Ser)-C6**.

Compound A-C6. The oxidative cleavage was carried out by dissolving **A(Ser)-C6** (220 mg, 0.22 mmol, 1 eq.) in H_2O (10 mM) and then adding $NaIO_4$ (471 mg, 2.2 mmol, 10 eq.). After 2 hours stirring, the desired product was obtained after preparative **[Prep-HPLC]** purification (gradient: 0 min, 100% A, 0% B; 5 min, 100% A, 0% B; 45 min, 0% A, 100% B). 47 mg were obtained (22%). **[HPLC 2]** t_R: 3.04 min. **HR-ESI-MS** m/z calcd for $[C_{40}H_{73}N_{17}O_{10}+2H]^{2+}$ 476.7936, found 476.7939; $[C_{40}H_{73}N_{17}O_{10}+3H]^{3+}$ 318.1982, found 318.1988; $[C_{40}H_{73}N_{17}O_{10}+H_2O+3H]^{3+}$ 324.2017, found 324.2031; $[C_{40}H_{73}N_{17}O_{10}+2H_2O+3H]^{3+}$ 330.2052, found 330.2063. **^{1}H NMR** (D_2O) δ_H: 5.29 (s, 2H, CH(H7 and H8)), 4.40 – 4.30 (m, 3H, $H\alpha_R$), 4.30 – 4.18 (m, 2H, $H\alpha_K$), 3.30 – 3.15 (m, 6H, $H\delta_R$) (m, 4H, $H\varepsilon_K$), 2.29 (td, ^{3}J = 7.2, ^{4}J = 2.0, 2H, CH_2-CO(H2)), 1.93 – 1.20 (m, 4H, $H\beta_K$) (m, 4H, $H\gamma_K$) (m, 4H, $H\delta_K$) (m, 6H, $H\beta_R$) (m, 6H, $H\gamma_R$) (m, 6H, CH_2(H3-H5)), 0.87 (t, ^{3}J = 6.9, 3H, CH_3(H6)).

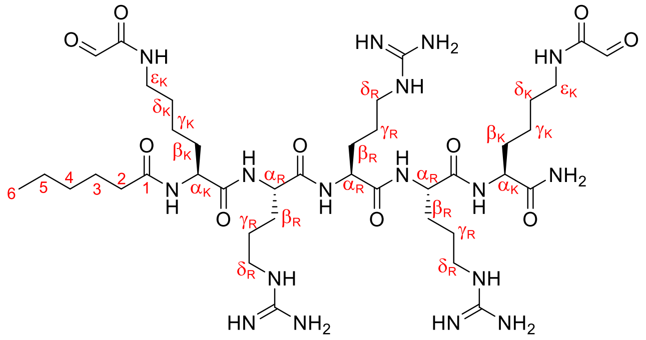

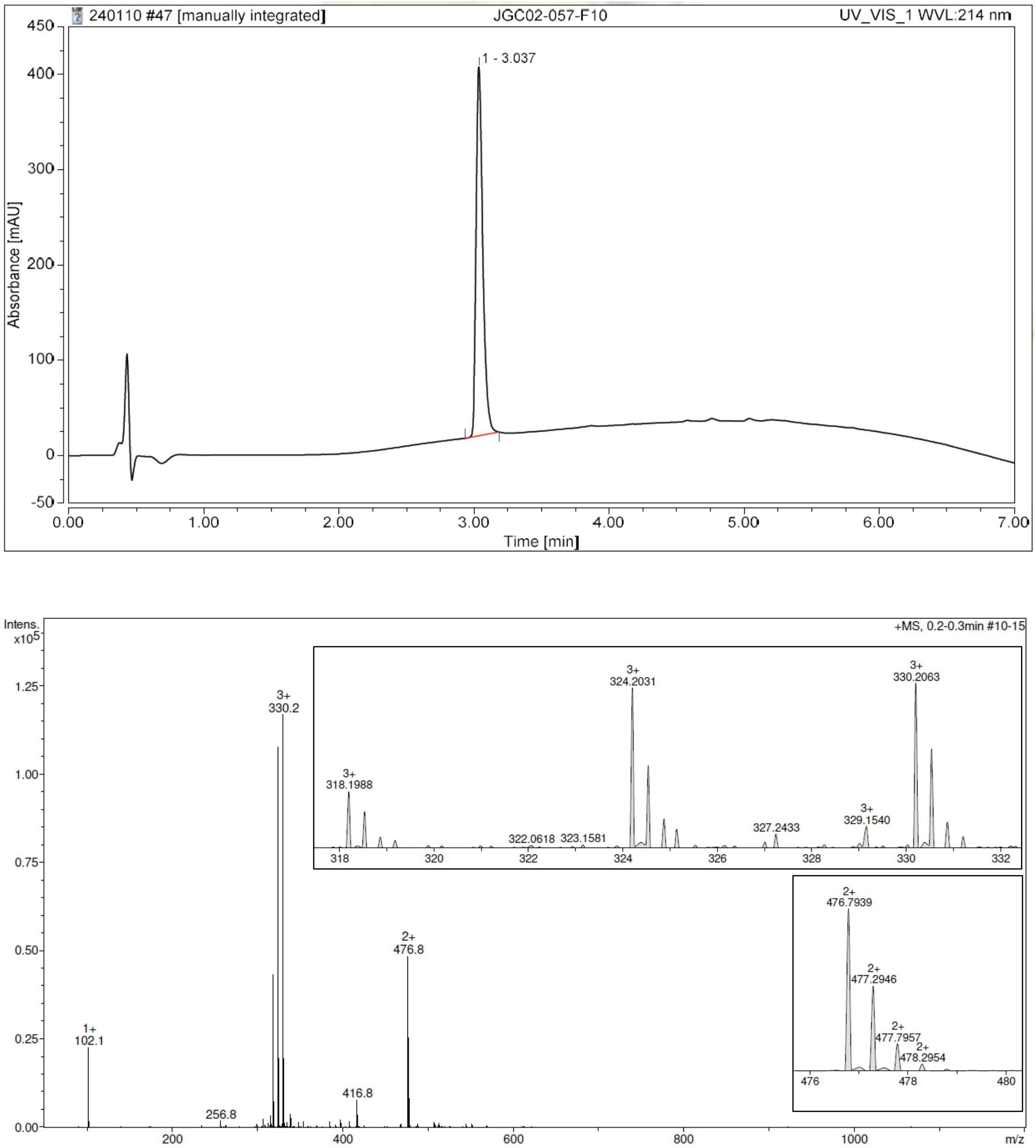

Figure 7. HPLC chromatogram (top) and HR-ESI-MS spectra (bottom) of compound **A-C6**.

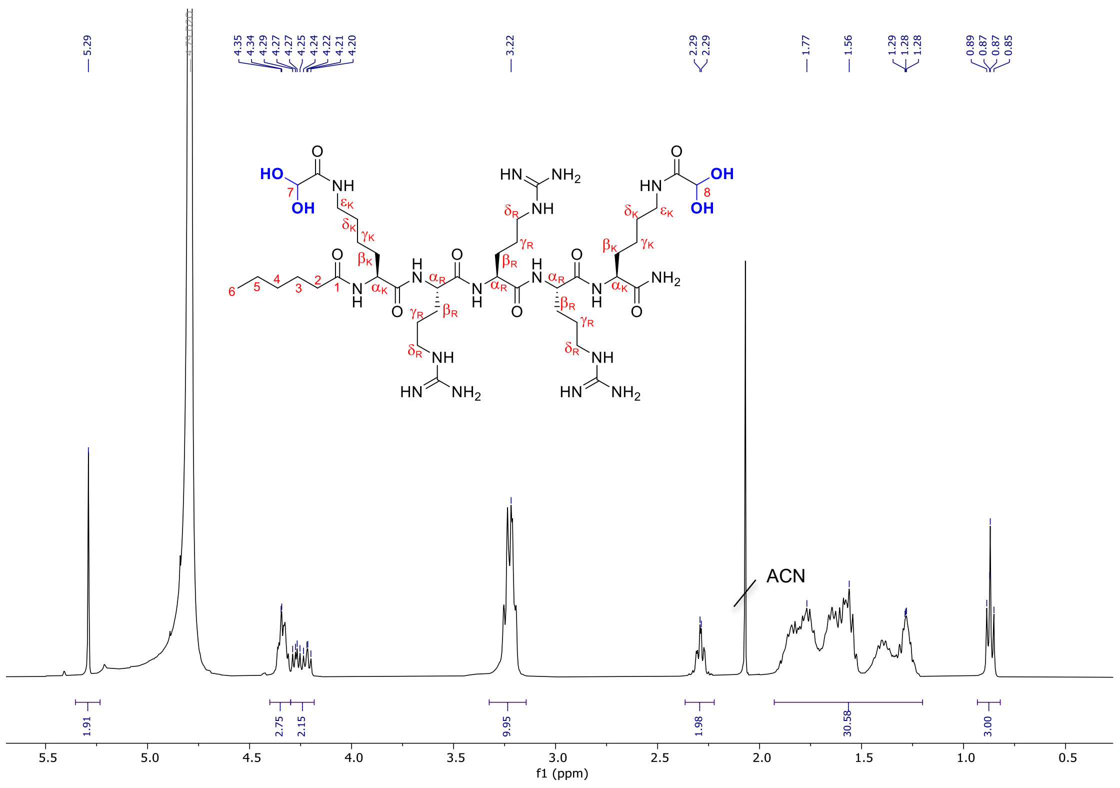

Figure 8. ^1H NMR (D_2O) spectrum of compound **A-C6** which was found to exist exclusively in its hydrated form depicted here.

b) Compound A-C10

Compound A(Ser)-C10. The peptide was synthesized according to the general **[SPPS 2]** procedure using Fmoc-*L*-Arg(Pbf)-OH, Fmoc-*L*-Lys[Boc-*L*-Ser(*O*tBu)]-OH and decanoic acid.

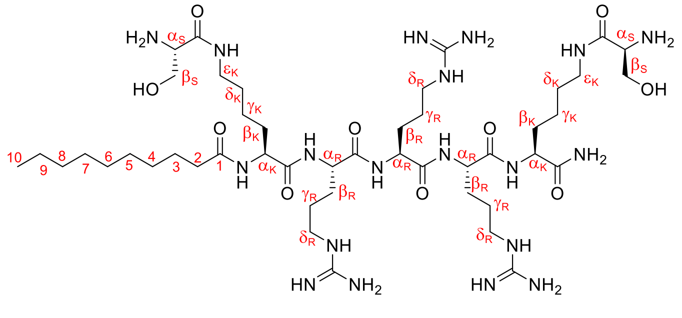

The solution was then concentrated, precipitated by adding ice cold Et_2O, and centrifuged. The supernatant was removed, and the crude material was freeze-dried. The compound **A(Ser)-C10** was obtained after preparative **[Prep-HPLC]** purification (gradient: 0 min, 100% A, 0% B; 5 min, 100% A, 0% B; 45 min, 0% A, 100% B). 80 mg were obtained (50%). **[HPLC 1]** t_R: 3.55 min. **HRESI-MS** m/z calcd for $[C_{46}H_{91}N_{19}O_{10}+2H]^{2+}$ 535.8671, found 535.8668; $[C_{46}H_{91}N_{19}O_{10}+3H]^{3+}$ 357.5805, found 357.5811. **^{1}H NMR** (400 MHz, D_2O) δH: 4.41 – 4.30 (m, 3H, $H\alpha_R$), 4.30 – 4.16 (m, 2H, $H\alpha_K$), 4.14 – 4.07 (m, 2H, $H\alpha_S$), 4.04 – 3.96 (dd, ^2J = 12.3, ^3J = 5.8, 2H, $H\beta_S$), 3.95 – 3.88 (dd, ^2J = 12.3, ^3J = 5.8, 2H, $H\beta'_S$), 3.34 – 3.16 (m, 6H, $H\delta_R$) (m, 4H, $H\varepsilon_K$), 2.30 (t, ^3J = 7.2, 2H, CH_2-CO(H2)), 1.98 – 1.50 (m, 4H, $H\beta_K$) (m, 4H, $H\delta_K$) (m, 6H, $H\beta_R$) (m, 6H, $H\gamma_R$), 1.50 – 1.18 (m, 4H, $H\gamma_K$) (m, 14H, CH_2(H3-H9)), 0.86 (t, ^3J = 6.9, 3H, CH_3(H10)).

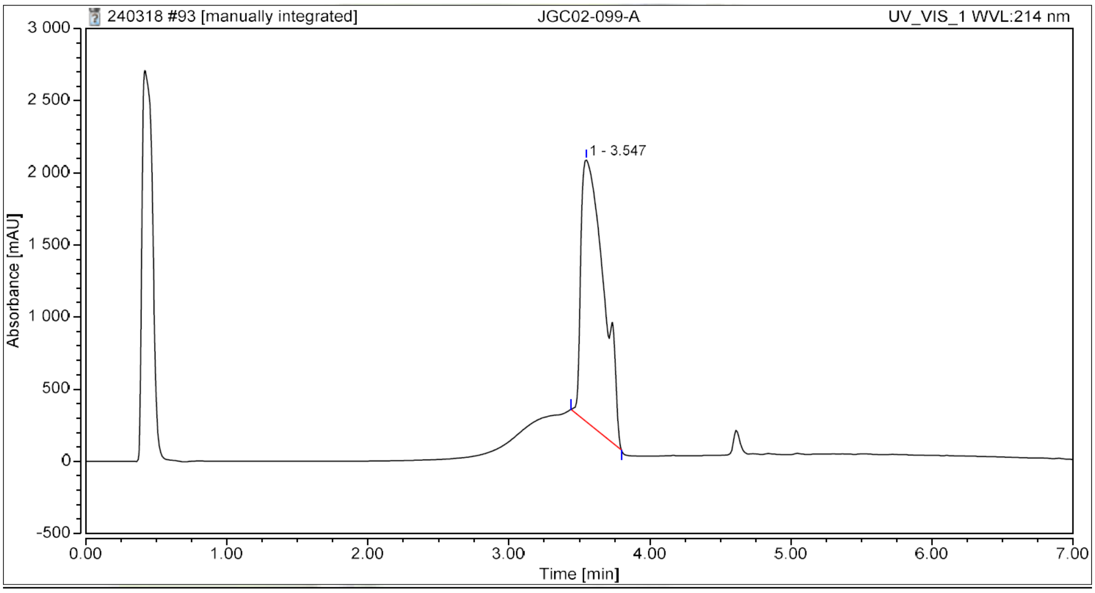

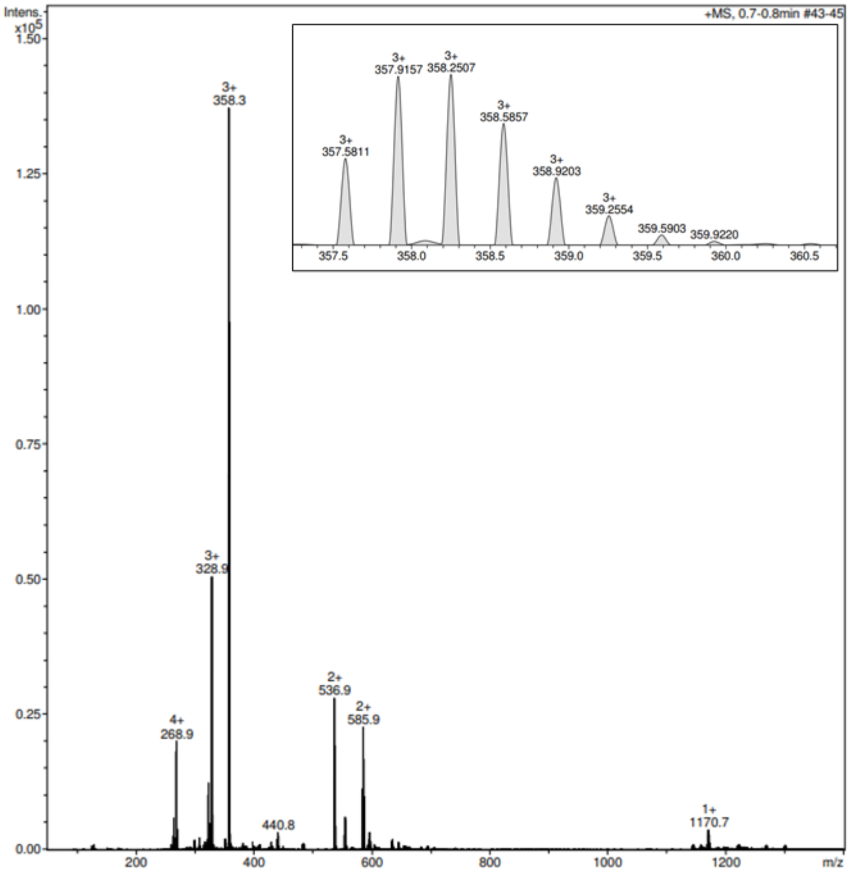

Figure 9. HPLC chromatogram (top) and HR-ESI-MS spectra (bottom) of compound **A(Ser)-C10**.

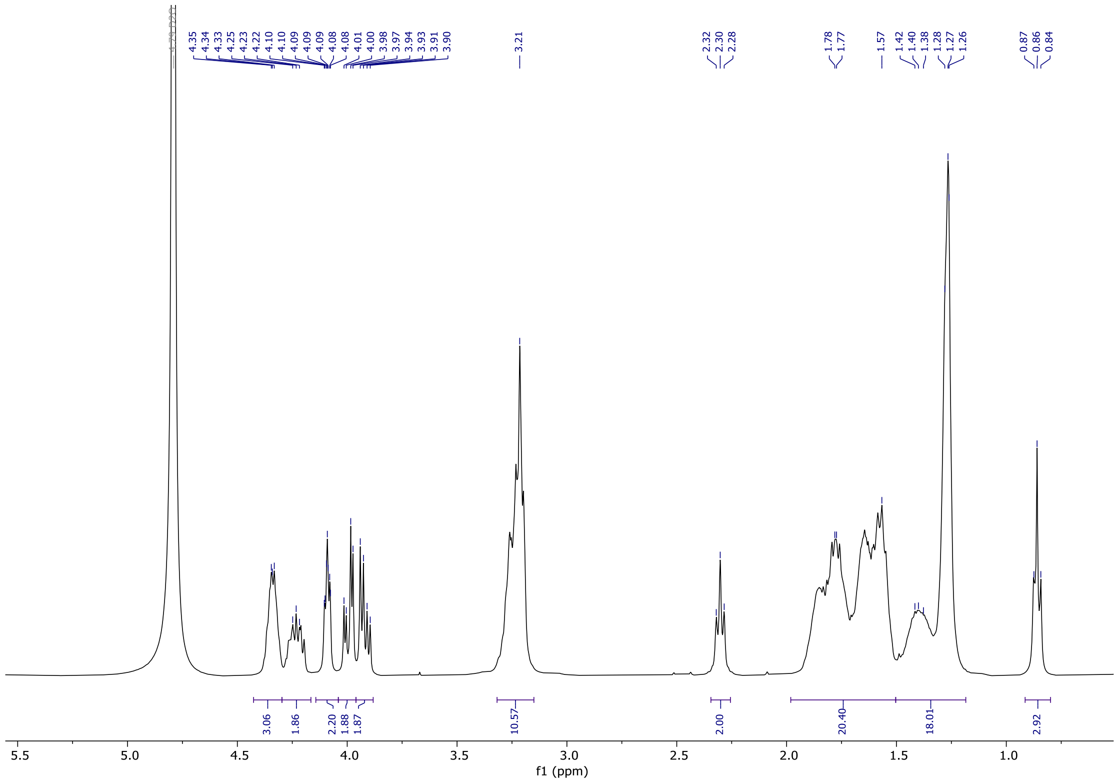

Figure 10. ^{1}H NMR (D_2O) spectrum of compound **A(Ser)-C10**.

Compound A-C10. The oxidative cleavage was carried out by dissolving **A(Ser)-C10** (80 mg, 0.074 mmol, 1 eq.) in H_2O (10 mM) and then adding $NaIO_4$ (160 mg, 0,74 mmol, 10 eq.). After 2 hours stirring, the desired product was obtained after preparative **[Prep-HPLC]** purification (gradient: 0 min, 100% A, 0% B; 5 min, 100% A, 0% B; 45 min, 0% A, 100% B). 43 mg were obtained (57%). **[HPLC 1]** t_R: 3.82 min. **HR-ESI-MS** m/z calcd for $[C_{44}H_{81}N_{17}O_{10}+2H]^{2+}$ 504.8251, found 504.8249; $[C_{44}H_{81}N_{17}O_{10}+3H]^{3+}$ 336.8857, found 336.8865. **^{1}H NMR** (400 MHz, D_2O) δH: 5.29 (s, 2H, CH-C(O)), 4.39 – 4.16 (m, 3H, $H\alpha_R$) (m, 2H, $H\alpha_K$), 4.14 – 4.07 (m, 2H, $H\alpha_S$), 3.32 – 3.14 (m, 6H, $H\delta_R$) (m, 4H, $H\varepsilon_K$), 2.30 (td, ^3J = 7.2, ^4J = 2.0, 2H, CH_2-CO(H2)), 1.93 – 1.43 (m, 4H, $H\beta_K$) (m, 4H, $H\delta_K$) (m, 6H, $H\beta_R$) (m, 6H, $H\gamma_R$), 1.48 – 1.17 (m, 4H, $H\gamma_K$) (m, 14H, CH_2(H3-H9)), 0.86 (t, ^3J = 6.9, 3H, CH_3(H10)).

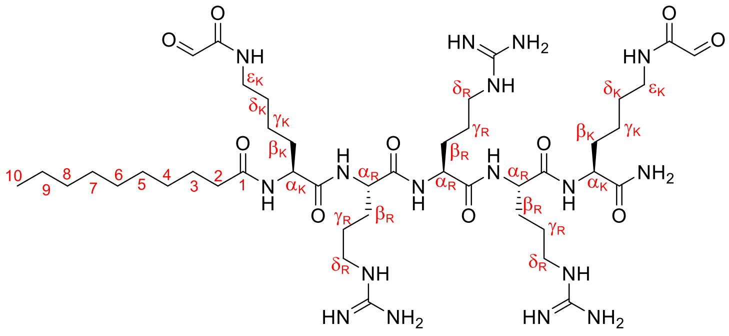

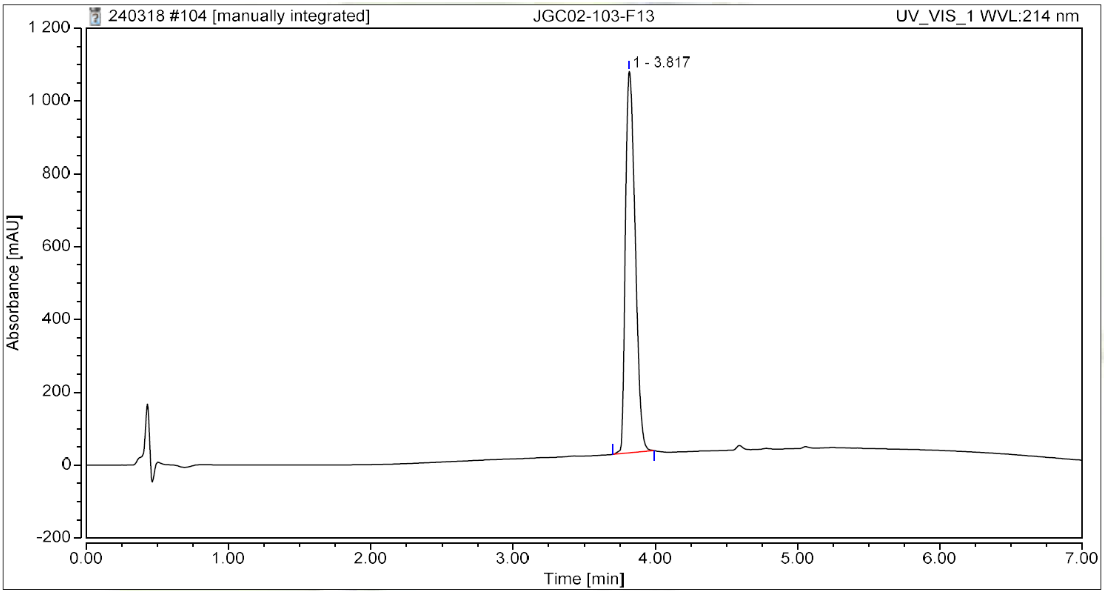

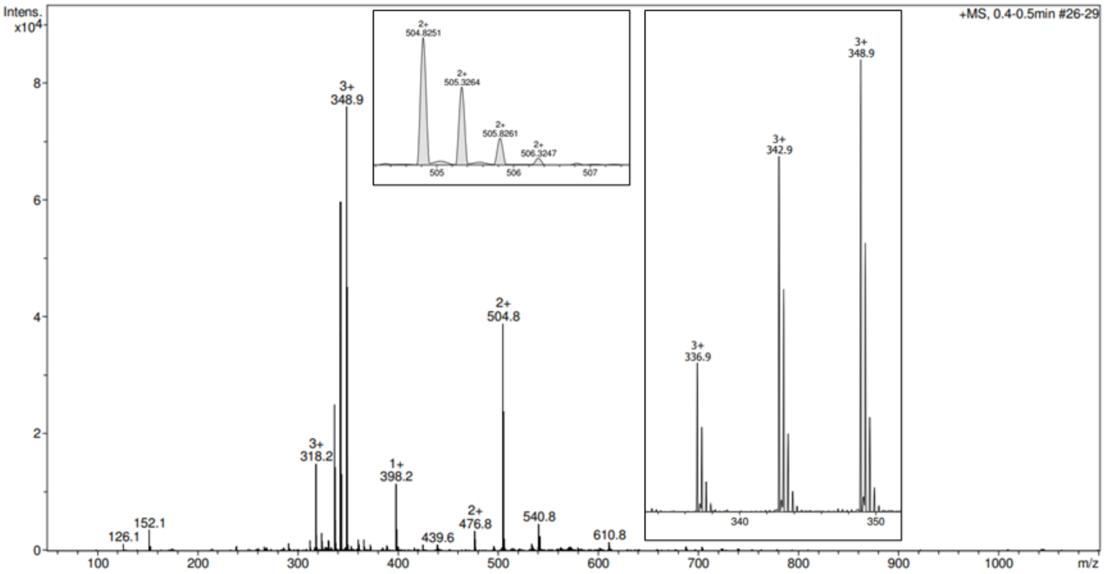

Figure 11. HPLC chromatogram (top) and HR-ESI-MS spectra (bottom) of compound **A)-C10**.

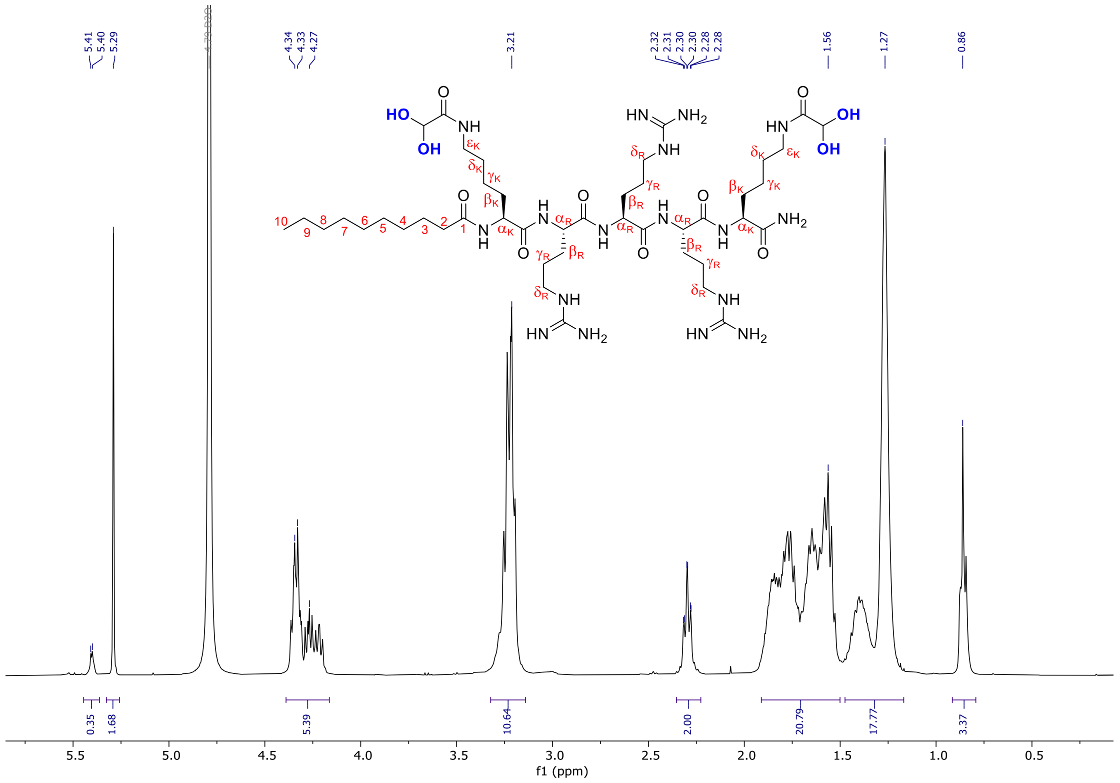

Figure 12. ^{1}H NMR (D_2O) spectrum of compound **A(Ser)-C10** which was found to exist exclusively in its hydrated form depicted here.

c) Compound A-C12

Compound A(Ser)-C12. The peptide was synthesized according to the general **[SPPS 2]** procedure using Fmoc-*L*-Arg(Pbf)-OH, Fmoc-*L*-Lys[Boc-*L*-Ser(O*t*Bu)]-OH and dodecanoic acid.

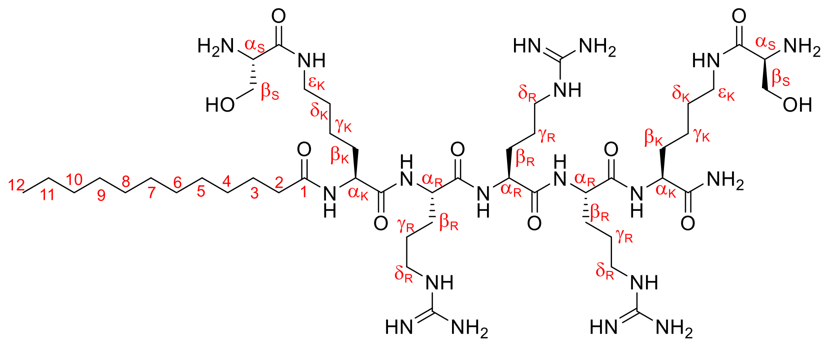

The solution was then concentrated, precipitated by adding ice cold Et_2O, and centrifuged. The supernatant was removed, and the crude material was freeze-dried. The compound **A(Ser)-C12** was obtained after preparative **[Prep-HPLC]** purification (gradient: 0 min, 100% A, 0% B; 5 min, 100% A, 0% B; 45 min, 0% A, 100% B). 250 mg were obtained (78%). **[HPLC 1]** t_R: 4.04 min. **HR-ESI-MS** m/z calcd for $[C_{48}H_{95}N_{19}O_{10}+2H]^{2+}$ 549.8827, found 549.8825; $[C_{48}H_{95}N_{19}O_{10}+3H]^{3+}$ 366.9243, found 366.9244. **^{1}H NMR** (MeOD) δ_H: 4.44 – 4.24 (m, 3H, $H\alpha_R$) (m, 1H, $H\alpha_K$) , 4.24 – 4.14 (m, 1H, $H\alpha_K$), 3.97 – 3.89 (m, 4H, $H\beta_S$), 3.87 – 3.78 (m, 2H, $H\alpha_S$), 3.28 – 3.15 (m, 6H, $H\delta_R$) (m, 4H, $H\varepsilon_K$), 2.26 (td, ^3J = 7.2, ^4J = 2.0, 2H, CH_2-CO(H2)), 1.96 – 1.24 (m, 4H, $H\beta_K$) (m, 4H, $H\gamma_K$) (m, 4H, $H\delta_K$) (m, 6H, $H\beta_R$) (m, 6H, $H\gamma_R$) (m, 18H, CH_2(H3-H11)), 0.87 (t, ^3J = 6.9, 3H, CH_3(H12)).

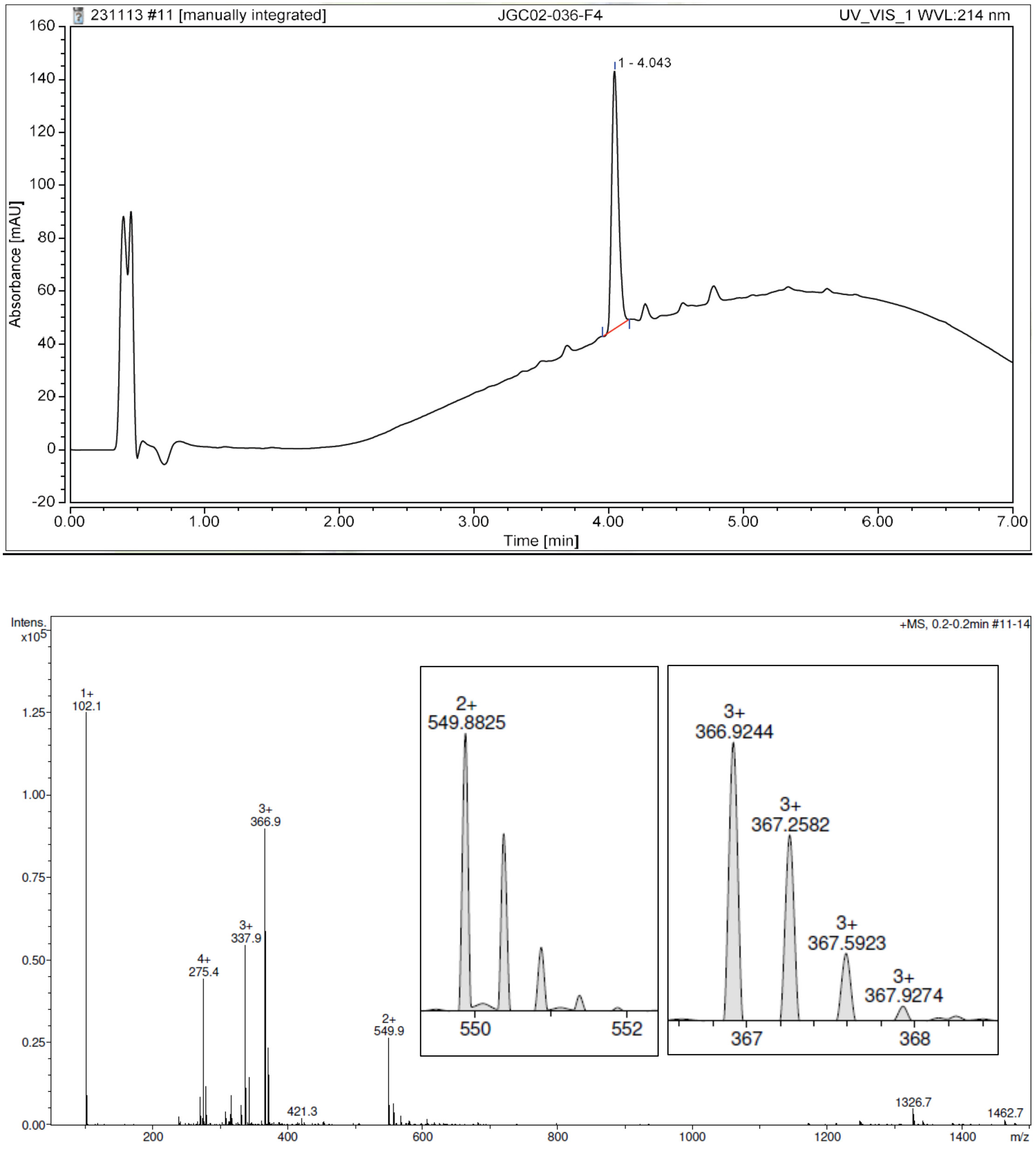

Figure 13. HPLC chromatogram (top) and HR-ESI-MS spectra (bottom) of compound **A(Ser)-C12**.

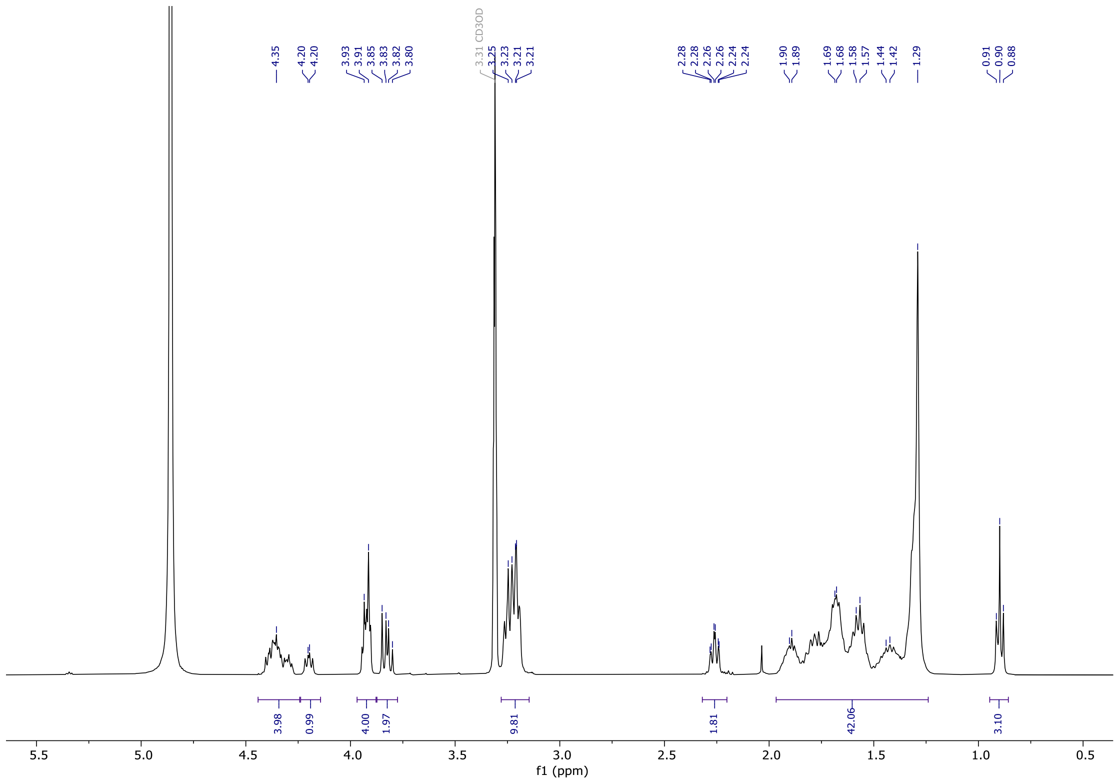

Figure 14. ^{1}H NMR (MeOD) spectrum of compound **A(Ser)-C12**.

Compound A-C12. The oxidative cleavage was carried out by dissolving **A(Ser)-C12** (250 mg, 0.23 mmol, 1 eq.) in H_2O (10 mM) and then adding $NaIO_4$ (487 mg, 2.3 mmol, 10 eq.). After 2 hours stirring, the desired product was obtained after preparative **[Prep-HPLC]** purification

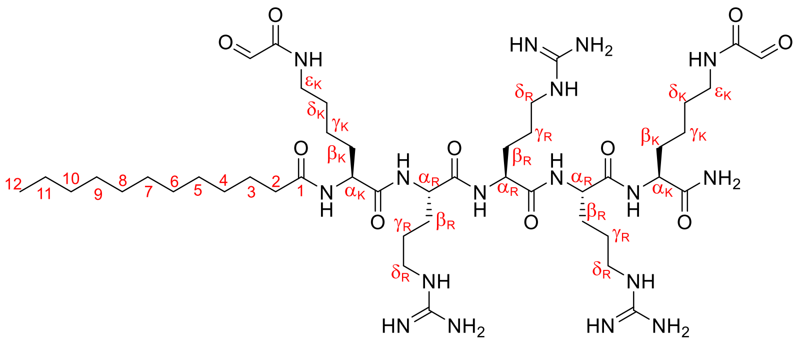

(gradient: 0 min, 100% A, 0% B; 5 min, 100% A, 0% B; 45 min, 0% A, 100% B). 44 mg were obtained (17%). **[HPLC 1]** t_R: 4.15 min. **HR-ESI-MS** m/z calcd for $[C_{46}H_{85}N_{17}O_{10}+2H]^{2+}$ 518.8405, found 518.8410; $[C_{46}H_{85}N_{17}O_{10}+3H]^{3+}$ 346.2295, found 346.2305; $[C_{46}H_{85}N_{17}O_{10}+H_2O+3H]^{3+}$ 352.2330, found 352.2343; $[C_{46}H_{85}N_{17}O_{10}+2H_2O+3H]^{3+}$ 358.2365, found 358.2378. **^{1}H NMR** (MeOD) δ_H: 4.41 – 4.25 (m, 3H, $H\alpha_R$) (m, 1H, $H\alpha_K$) , 4.24 – 4.14 (m, 1H, $H\alpha_K$), 3.29 – 3.13 (m, 6H, $H\delta_R$) (m, 4H, $H\varepsilon_K$), 2.26 (td, ^3J = 7.2, ^4J = 2.0, 2H, CH_2-CO(H2)), 1.98 – 1.20 (m, 4H, $H\beta_K$) (m, 4H, $H\gamma_K$) (m, 4H, $H\delta_K$) (m, 6H, $H\beta_R$) (m, 6H, $H\gamma_R$) (m, 18H, CH_2(H3-H11)), 0.89 (t, ^3J = 6.9, 3H, CH_3(H12)).

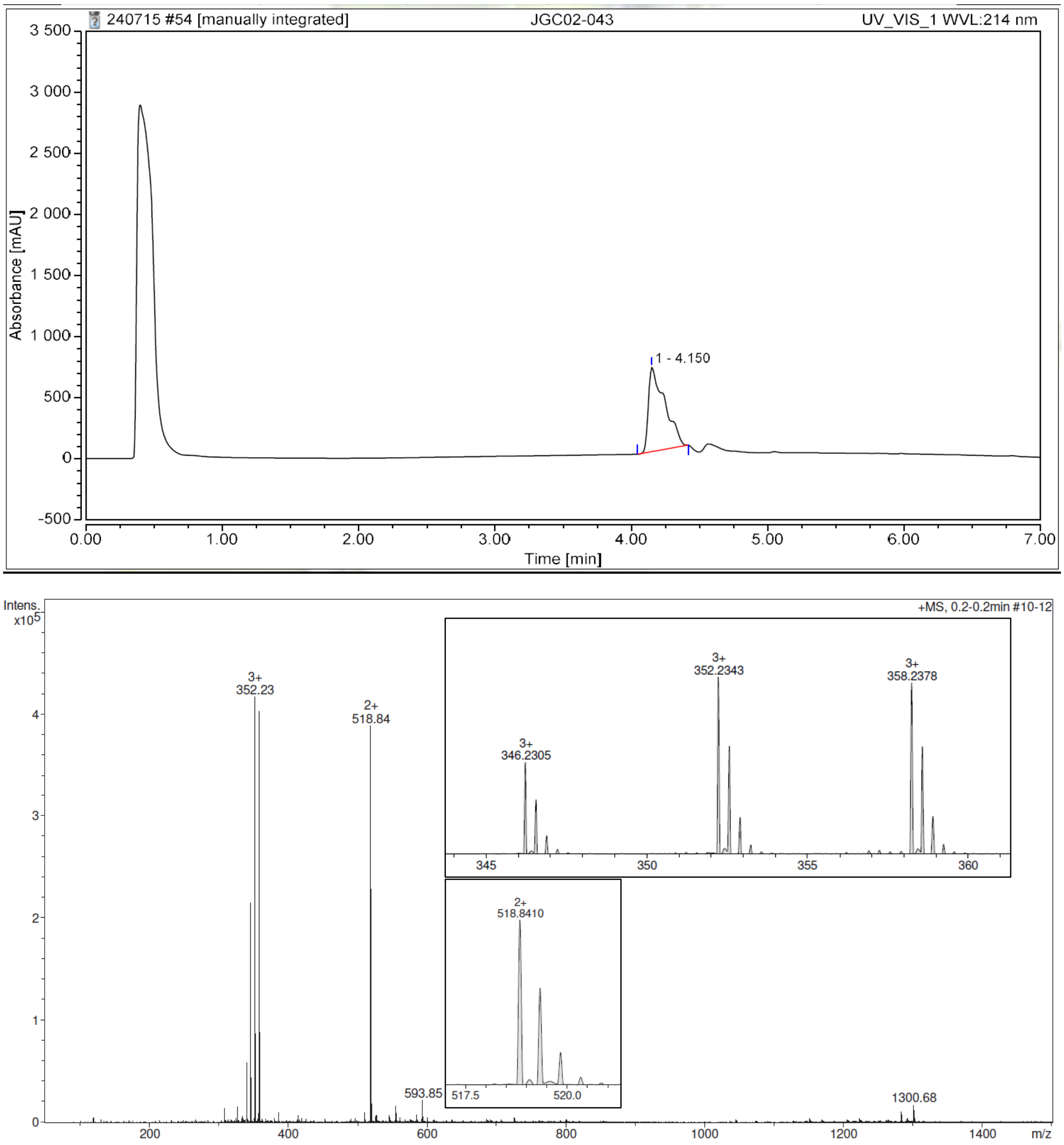

Figure 15. HPLC chromatogram (top) and HR-ESI-MS spectra (bottom) of compound **A-C12**.

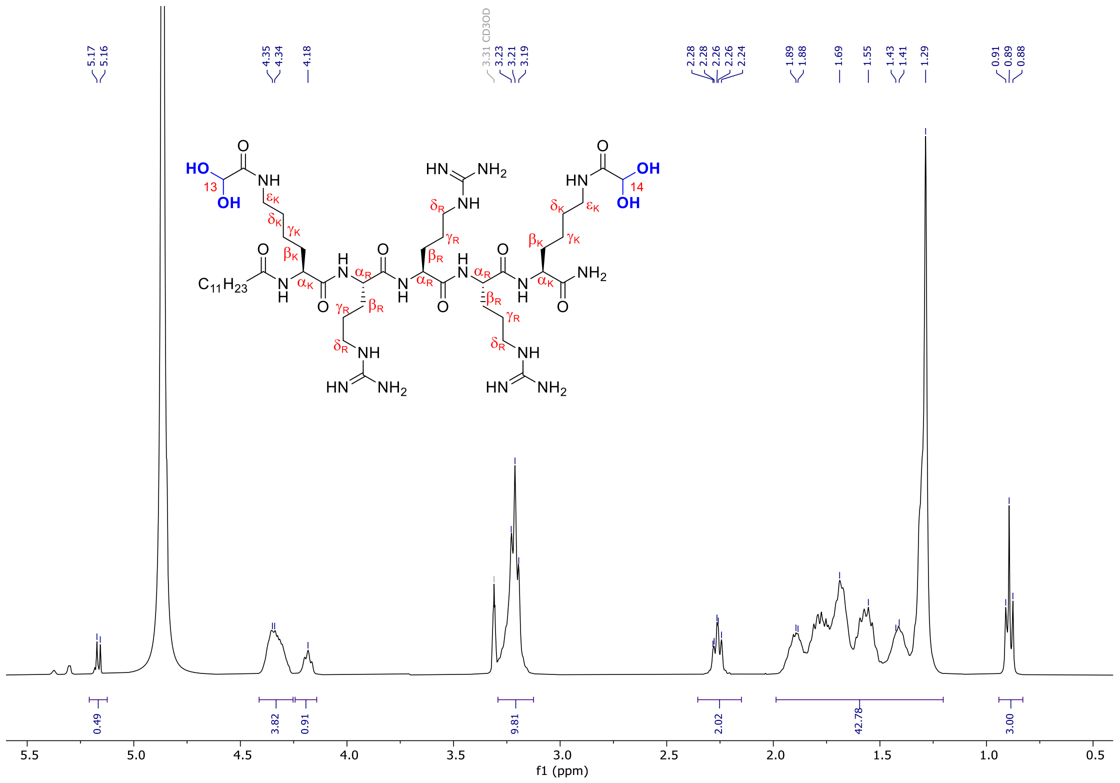

Figure 16. ^{1}H NMR (MeOD) spectrum of compound **A-C12** which was found to exist exclusively in its hydrated form depicted here.

d) Compound A-C18

Compound A(Ser)-C18. The peptide was synthesized according to the general **[SPPS 2]** procedure using Fmoc-*L*-Arg(Pbf)-OH, Fmoc-*L*-Lys[Boc-*L*-Ser(O*t*Bu)]-OH and octadecanoic acid. The solution was then concentrated, precipitated by adding ice cold Et_2O, and centrifuged. The supernatant was removed, and the crude material was freeze-dried. The compound

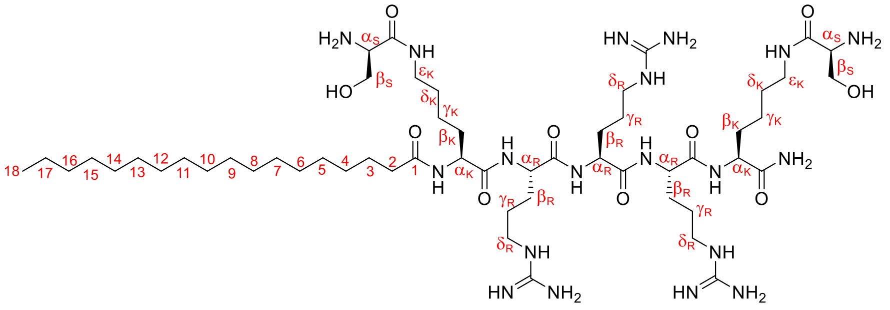

A(Ser)-C18 was obtained after preparative **[Prep-HPLC]** purification (gradient: 0 min, 70% A, 30% B; 5 min, 70% A, 30% B; 45 min, 0% A, 100% B). 260 mg were obtained (75%). **[HPLC 1]** t_R: 4.82 min. **HR-ESI-MS** m/z calcd for $[C_{54}H_{107}N_{19}O_{10}+2H]^{2+}$ 591.9297, found 591.9309; $[C_{54}H_{107}N_{19}O_{10}+3H]^{3+}$ 394.9556, found 394.9563. **^{1}H NMR** (MeOD) δ_H: 4.43 – 4.15 (m, 3H, Hα_R) (m, 2H, Hα_K), 3.98 – 3.86 (m, 4H, Hβ_S), 3.86 – 3.76 (m, 2H, Hα_S), 3.28 – 3.15 (m, 6H, Hδ_R) (m, 4H, Hε_K), 2.27 (td, ^{3}J = 7.2, ^{4}J = 2.0, 2H, CH_2-CO(H2)), 2.08 – 1.22 (m, 4H, Hβ_K) (m, 4H, Hγ_K) (m, 4H, Hδ_K) (m, 6H, Hβ_R) (m, 6H, Hγ_R) (m, 30H, CH_2(H3-H17)), 0.90 (t, ^{3}J = 6.9, 3H, CH_3(H18)).

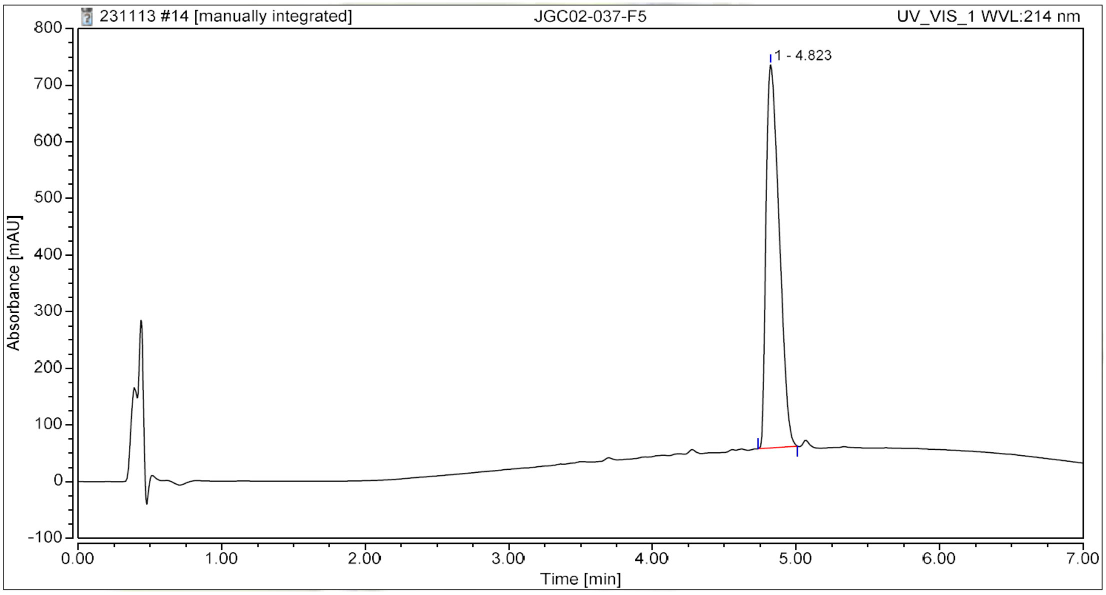

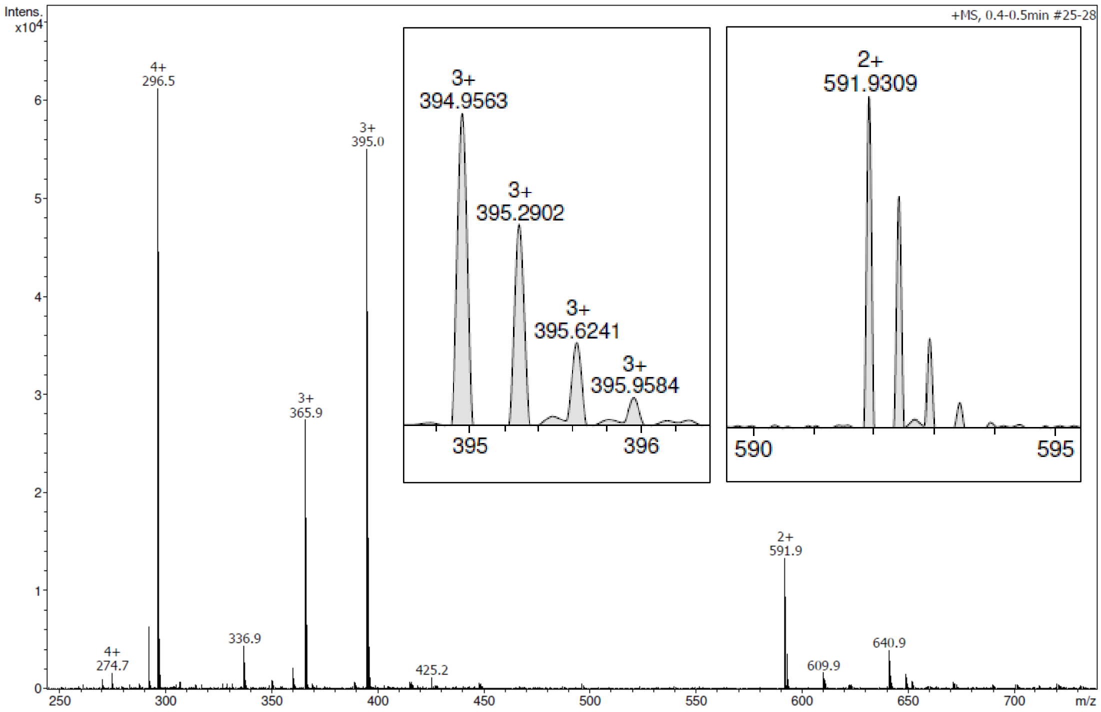

Figure 17. HPLC chromatogram (top) and HR-ESI-MS spectra (bottom) of compound **A(Ser)-C18**.

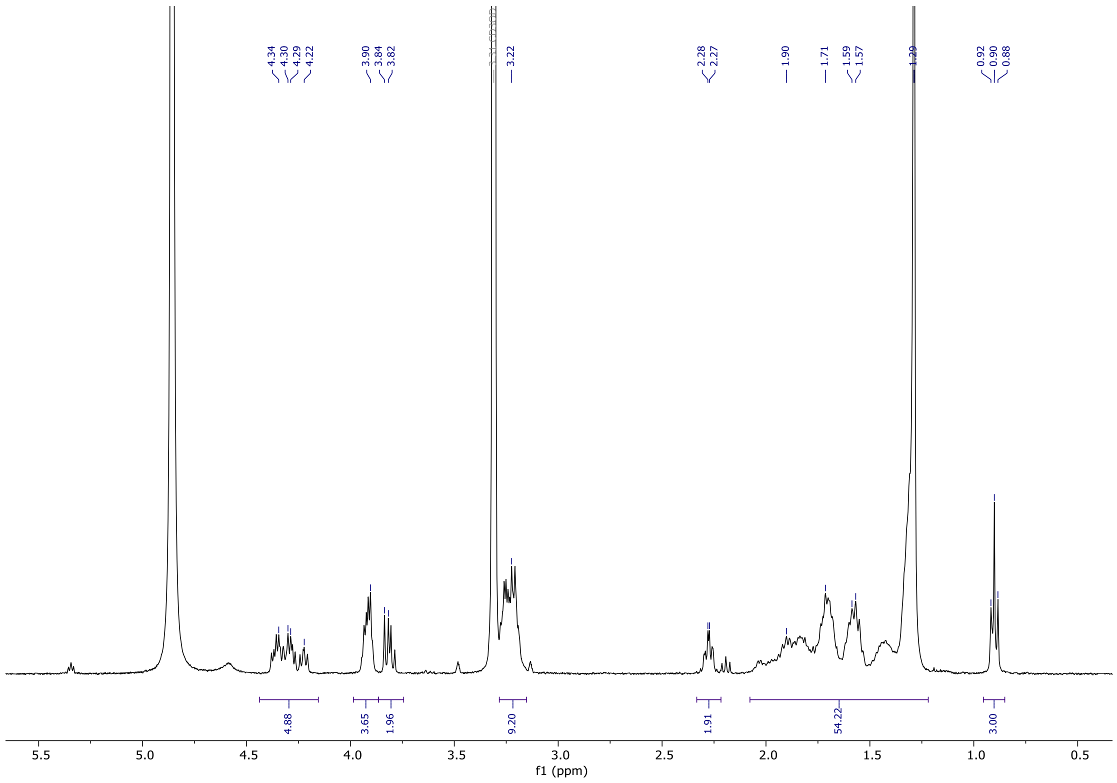

Figure 18. ^{1}H NMR (MeOD) spectrum of compound **A(Ser)-C18**.

Compound A-C18. The oxidative cleavage was carried out by dissolving **A(Ser)-C18** (260 mg, 0.22 mmol, 1 eq.) in H_2O/DMF (50:50; 10 mM) and then adding $NaIO_4$ (487 mg, 2.3 mmol, 10 eq.).

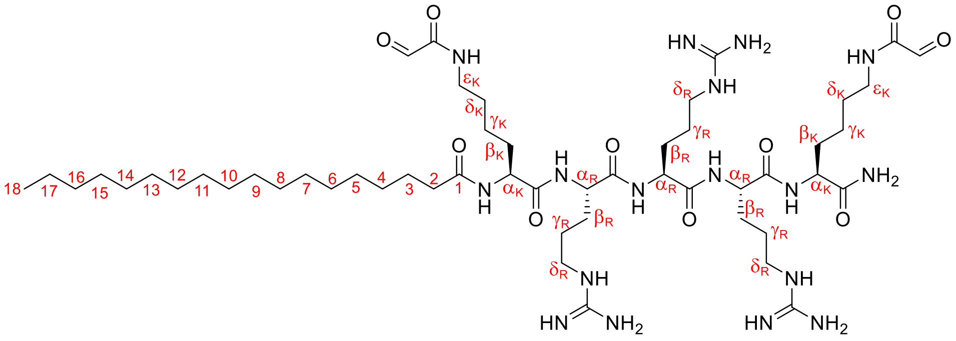

After 2 hours stirring, the desired product was obtained after preparative **[Prep-HPLC]** purification (gradient: 0 min, 70% A, 30% B; 5 min, 70% A, 30% B; 45 min, 0% A, 100% B). 11 mg were obtained (5%). **[HPLC 1]** t_R: 5.05 min. **HR-ESI-MS** m/z calcd for $[C_{52}H_{97}N_{17}O_{10}+2H]^{2+}$ 560.8875, found 560.8877; $[C_{52}H_{97}N_{17}O_{10}+3H]^{3+}$ 374.2608, found 374.2611; $[C_{52}H_{97}N_{17}O_{10}+H_2O+3H]^{3+}$ 380.2643, found 380.2651; $[C_{52}H_{97}N_{17}O_{10}+2H_2O+3H]^{3+}$ 386.2678, found 386.2690.**^{1}H NMR** (MeOD) δ_H: 4.43 – 4.25 (m, 4H, Hα_R) (m, 1H, Hα_K), 4.24 – 4.13 (m, 1H, Hα_K), 3.29 – 3.12 (m, 6H, Hδ_R) (m, 4H, Hε_K), 2.26 (td, ^3J = 7.2, ^4J = 2.0, 2H, CH_2-CO(H2)), 1.97 – 1.23 (m, 4H, Hβ_K) (m, 4H, Hγ_K) (m, 4H, Hδ_K) (m, 6H, Hβ_R) (m, 6H, Hγ_R) (m, 30H, CH_2(H3-H17)), 0.90 (t, ^3J = 6.9, 3H, CH_3(H18)).

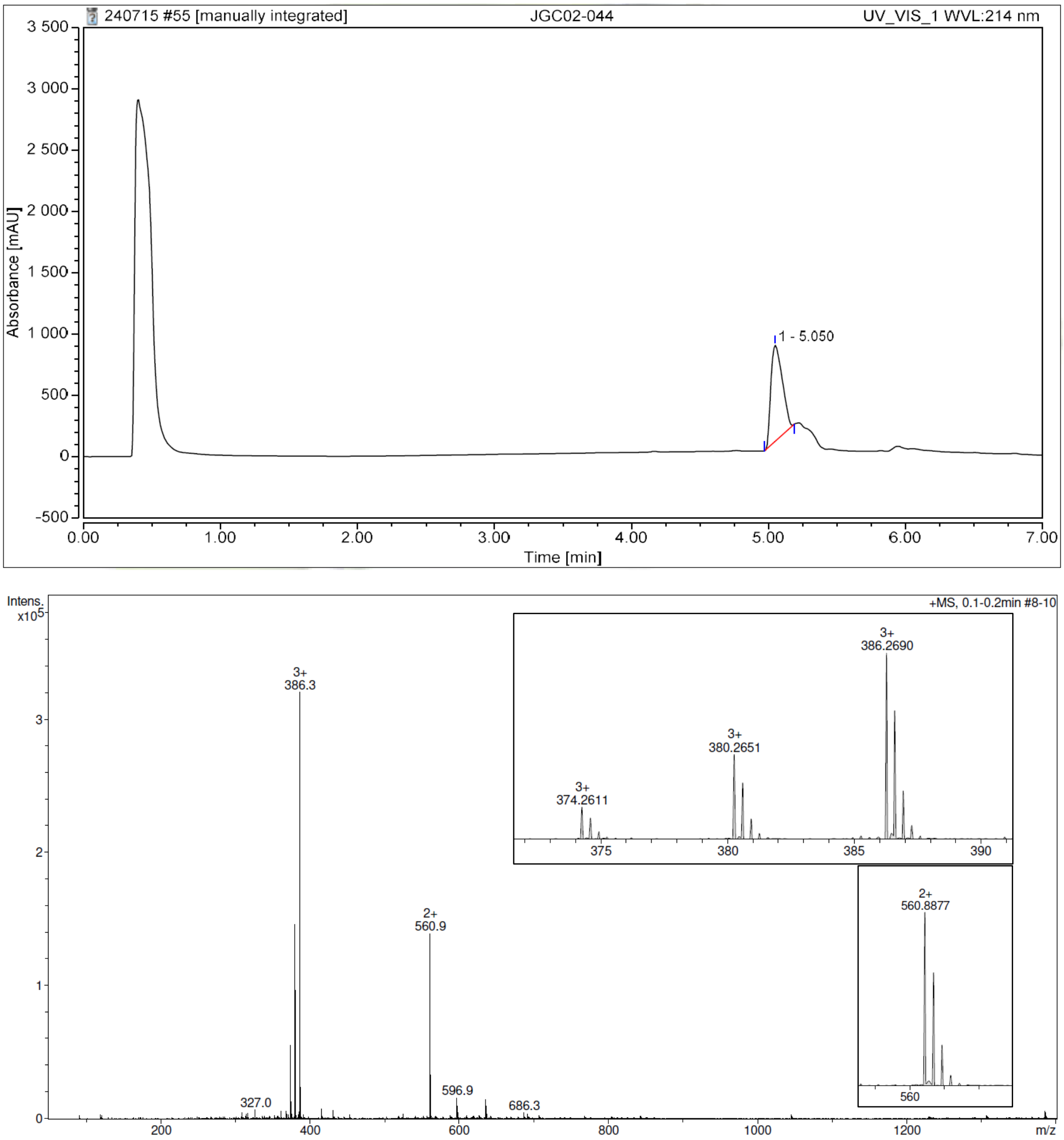

Figure 19. HPLC chromatogram (top) and HR-ESI-MS spectra (bottom) of compound **A-C18**.

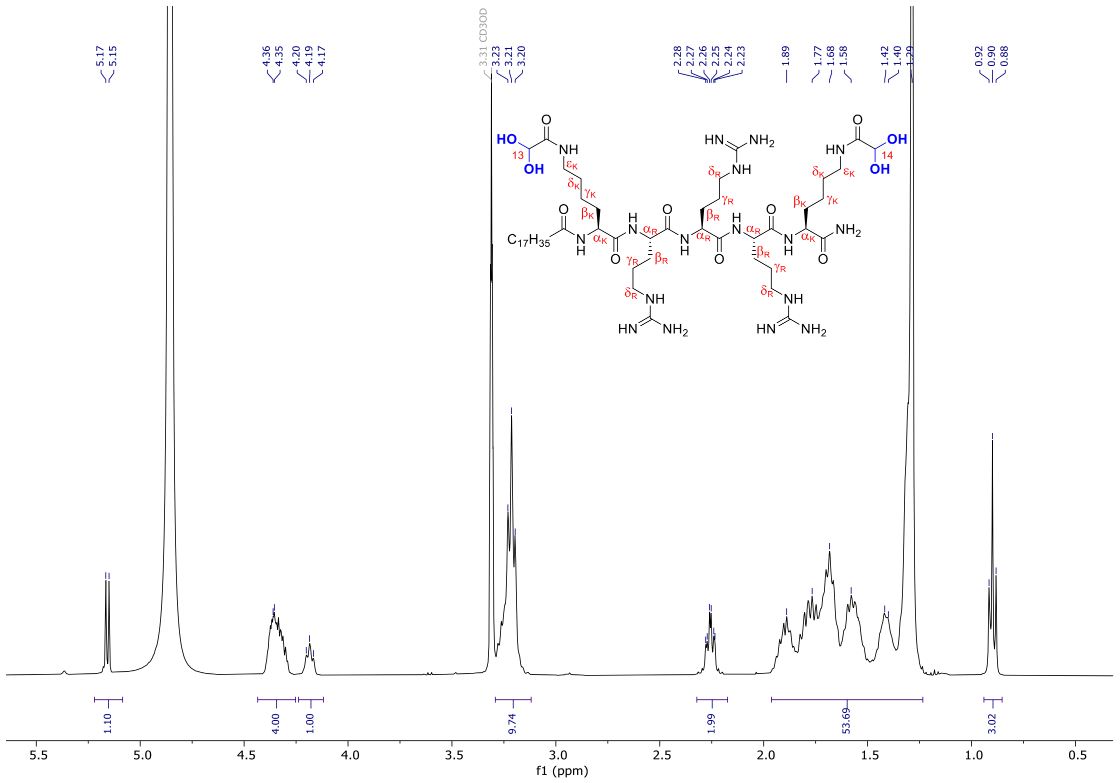

Figure 20. ¹H NMR (400 MHz, MeOD) spectrum of compound **A-C18** which was found to exist exclusively in its hydrated form depicted here.

4. Synthesis of Bishydrazide Peptides H and H-Cn

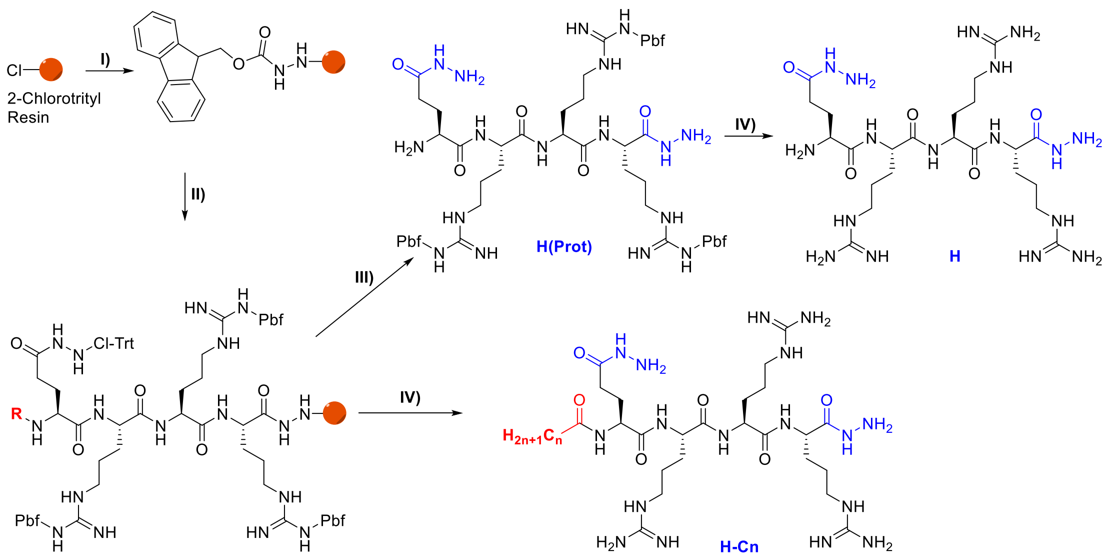

Scheme 3: Synthetic scheme for the preparation of the bishydrazide peptides **H** and **H-Cn**. I) Resin modification, II) SPPS, III) mild cleavage and Clt deprotection, and deprotection, IV) harsh cleavage and/or Pbf deprotection

a) Compound H

H-(Prot). The peptide was synthesized according to the general **[SPPS 1]** procedure using Fmoc-*L*-Arg(Pbf)-OH and Fmoc-*L*-Glu[NHNH(2Cl-Trt)]-OH. The crude product was obtained after freeze drying. The compound **H-(Prot)** was obtained after preparative **[Prep-HPLC]** purification (gradient: 0 min, 70% A, 30% B; 5 min, 70% A, 30% B; 30 min, 20% A, 80% B; 35 min, 100% B). 60 mg (20%) were obtained. **[HPLC 1]** t_R: 4.95 min. **ESI-MS** m/z calcd for $[C_{62}H_{97}N_{17}O_{14}S_3+H]^+$ 1400.66, found 1400.69; $[C_{62}H_{97}N_{17}O_{14}S_3+2H]^{2+}$ 700.83, found 700.84; $[C_{62}H_{97}N_{17}O_{14}S_3+3H]^{3+}$ 467.56, found 467.56.

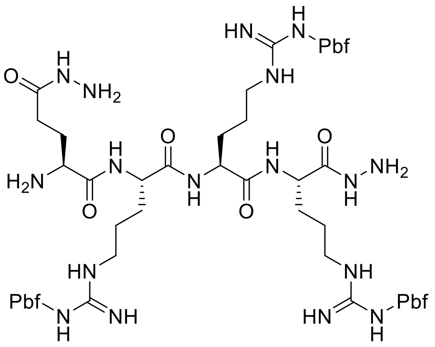

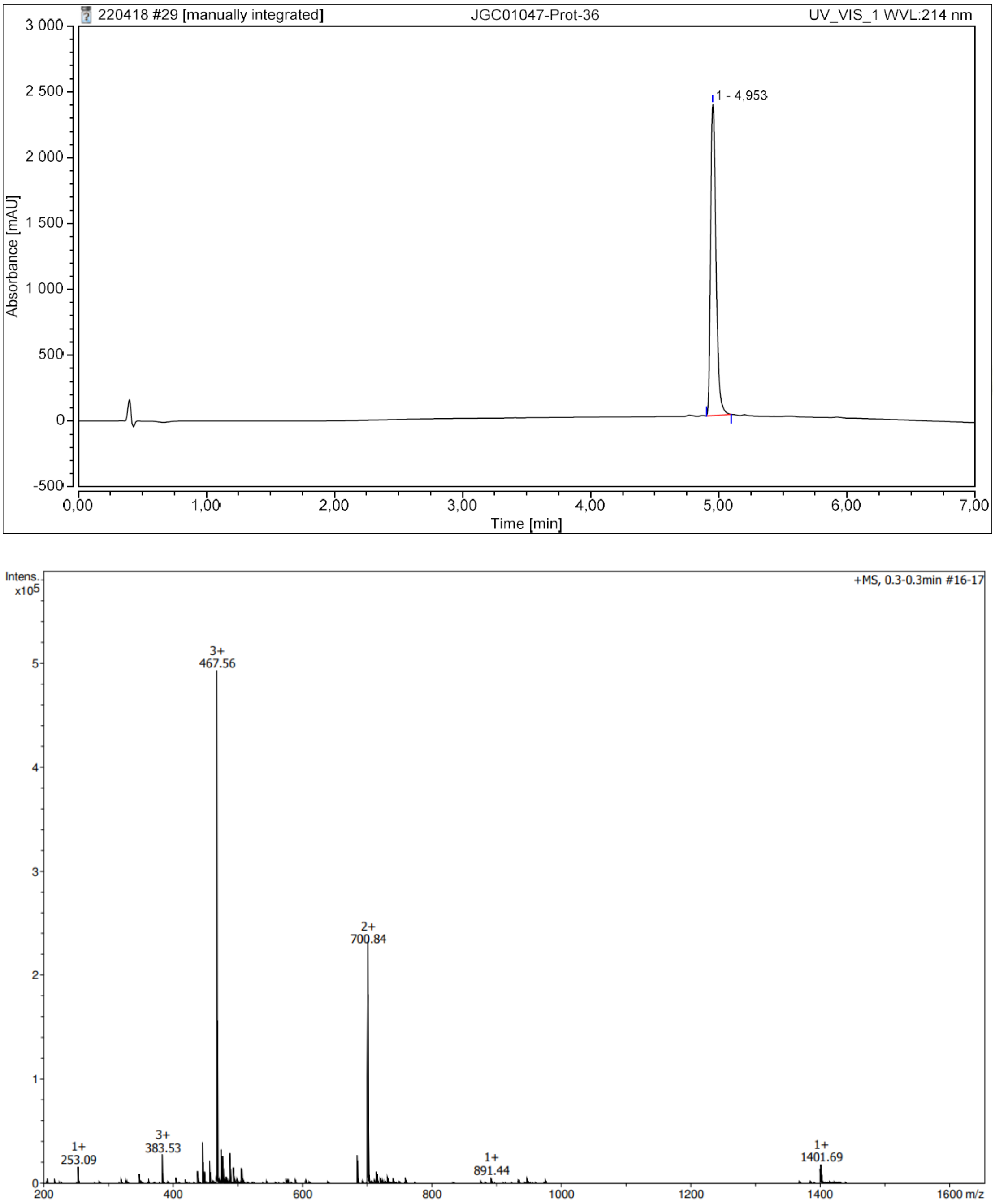

Figure 21. HPLC chromatogram (top) and ESI-MS spectra (bottom) of **H-(Prot).**

Compound H. Compound **H-(Prot)** was deprotected according to the general **[SPPS 1]** procedure. The solution was then concentrated, precipitated by adding ice cold Et_2O, and centrifuged. The supernatant was removed, and the crude material was freeze-dried to give 24 mg (50%) of a white solid. **LC-MS** m/z calcd for $[C_{23}H_{49}N_{17}O_5+H]^+$ 644.42, found 644.45; $[C_{23}H_{49}N_{17}O_5+TFA+H]^+$ 758.48, found 758.45; $[C_{23}H_{49}N_{17}O_5+2H]^{2+}$ 322.71, found 322.80; $[C_{23}H_{49}N_{17}O_5+TFA+2H]^{2+}$ 379.91, found 379.80; $[C_{23}H_{49}N_{17}O_5+3H]^{3+}$ 215.48, found 215.55. **^{1}H NMR** (D_2O) δ_H: 4.48 - 4.27 (m, 3H, $H\alpha_R$), 4.15 (t, ^3J = 6.5, 1H, $H\alpha_{E*}$), 3.29 - 3.13 (m, 6H, $H\delta_R$), 2.68 - 2.46 (m, 2H, $H\gamma_{E*}$), 2.26 - 2.16 (m, 2H, $H\beta_{E*}$), 1.93 - 1.75 (m, 6H, $H\beta_R$), 1.75 - 1.58 (m, 6H, $H\gamma_R$).

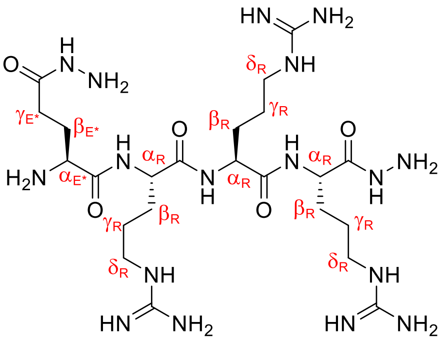

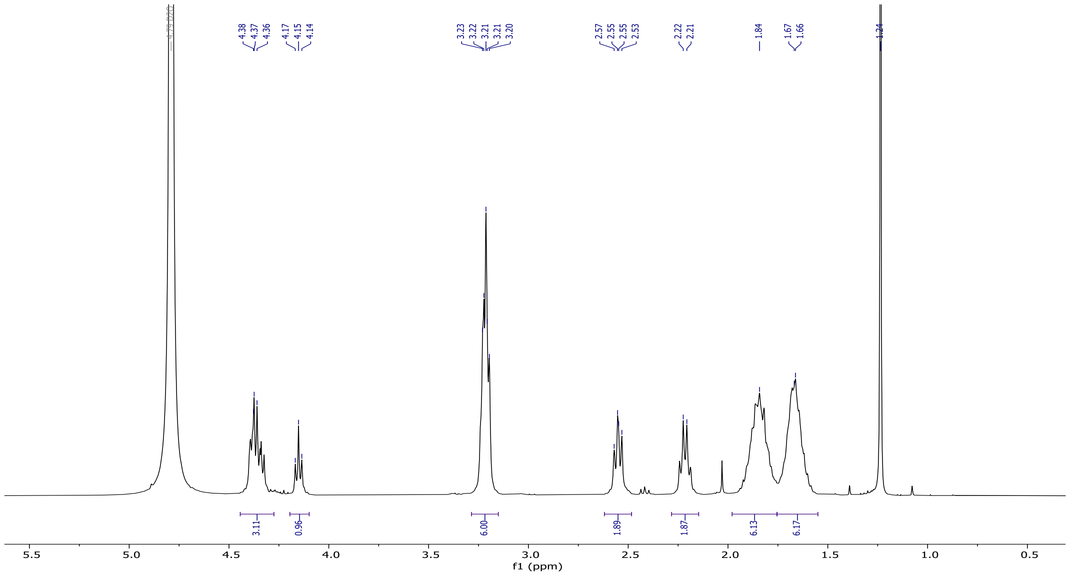

Figure 22. ^{1}H NMR (D_2O) spectrum of compound **H**.

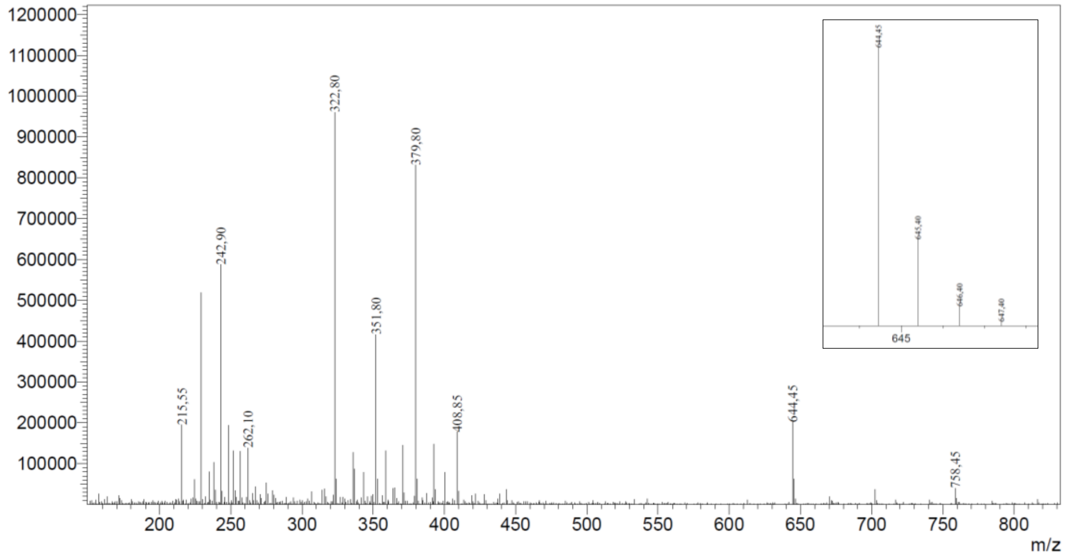

Figure 23. ESI-MS spectrum of compound **H**.

b) Compound H-C6

The peptide was synthesized according to the general **[SPPS 2]** procedure using Fmoc-*L*-Arg(Pbf)-OH, Fmoc-*L*-Glu[NHNH(2Cl-Trt)]-OH and hexanoic acid. The solution was then concentrated, precipitated by adding ice cold Et_2O, and centrifuged. The supernatant was removed, and the crude material was freeze-dried. The compound **H-C6** was obtained after preparative **[Prep-HPLC]** purification (gradient: 0 min, 100% A, 0% B; 5 min, 100% A, 0% B; 45 min, 0% A, 100% B). 18 mg were obtained (7%). **[HPLC 2]** t_R: 3.21 min. **HR-ESI-MS** m/z calcd for $[C_{29}H_{59}N_{17}O_6+H]^+$ 742.4907, found 742.4888; $[C_{29}H_{59}N_{17}O_6+2H]^{2+}$ 371.7490, found 371.7499. **^{1}H NMR** (D_2O) δ_H: 4.45 – 4.26 (m, 3H, Hα_R) (m, 1H, Hα_{E^*}), 3.29 – 3.14 (m, 6H, Hδ_R), 2.54 – 2.36 (m, 2H, Hγ_{E^*}), 2.29 (t, ^3J = 6.9, 2H, CH_2-CO(H2)), 2.22 – 2.07 (m, 2H, Hβ_{E^*}), 2.05 – 1.93 (m, 2H, Hβ'_{E^*}), 1.93 – 1.73 (m, 6H, Hβ_R), 1.73 – 1.52 (m, 6H, Hγ_R) (m, 2H, CH_2(H3)), 1.35 – 1.22 (m, 2H, CH_2(H4)) (m, 2H, CH_2(H5)), 0.86 (t, ^3J = 7.0, 3H, CH_3(H6)).

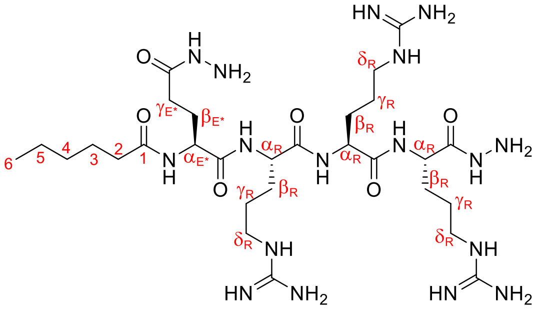

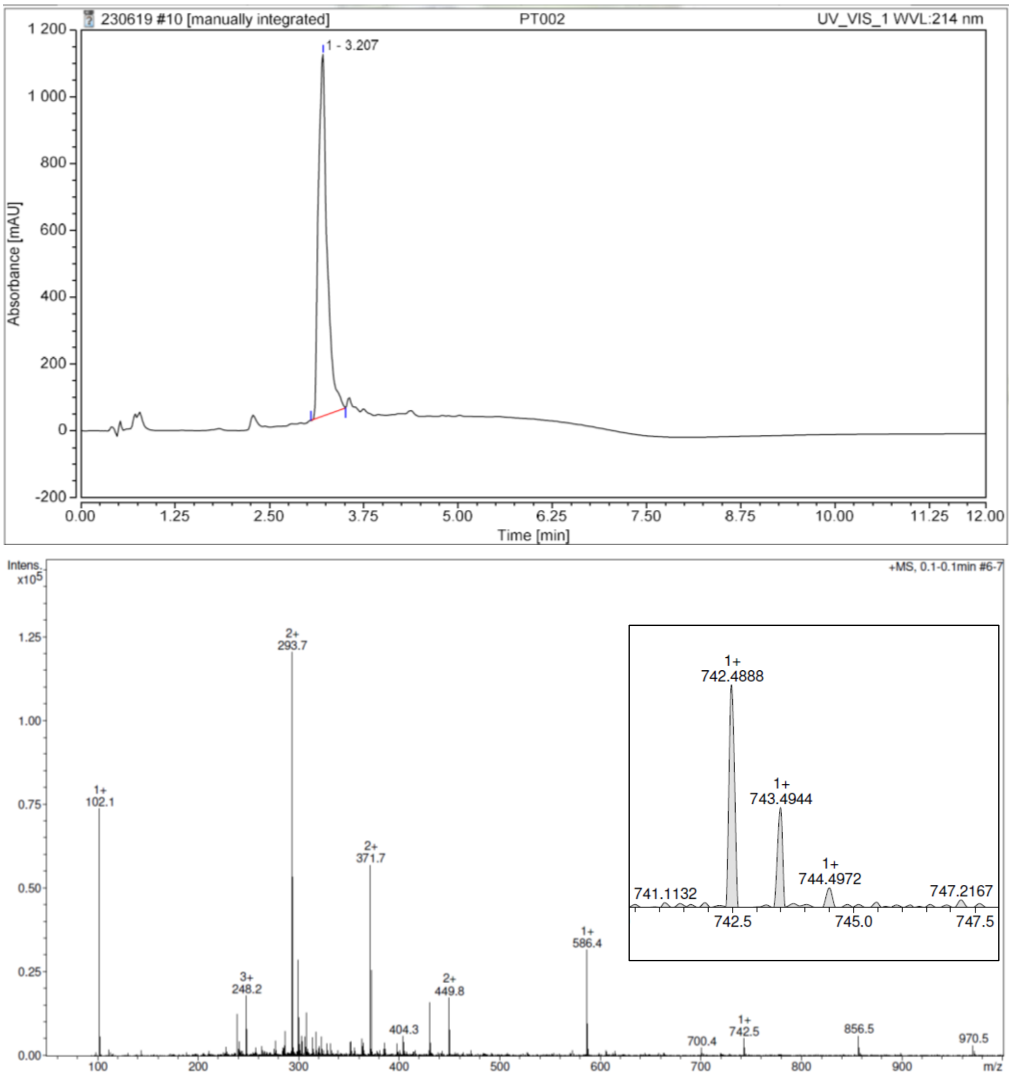

Figure 24. HPLC chromatogram (top) and HR-ESI-MS spectra (bottom) of compound **H-C6**.

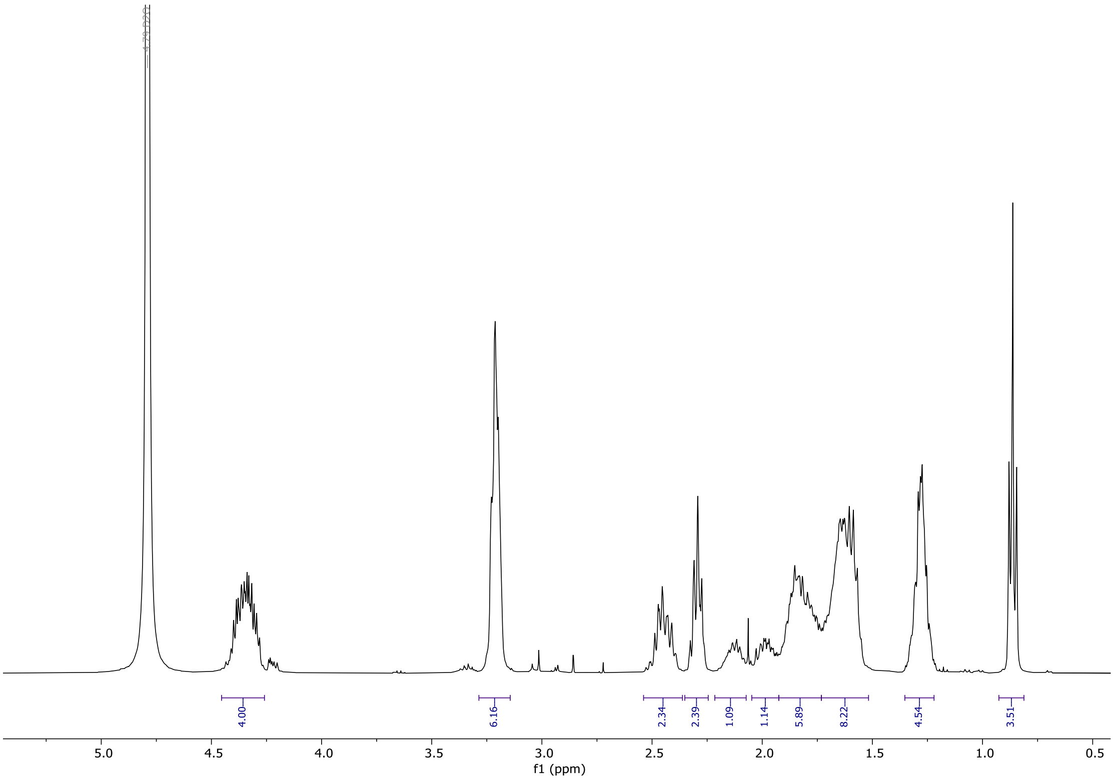

Figure 25. ^{1}H NMR (D_2O) spectrum of compound **H-C6**.

c) Compound H-C10

The peptide was synthesized according to the general **[SPPS 2]** procedure using Fmoc-*L*-Arg(Pbf)-OH, Fmoc-*L*-Glu[NHNH(2Cl-Trt)]-OH and decanoic acid. The solution was then concentrated, precipitated by adding ice cold Et_2O, and centrifuged. The supernatant was removed, and the crude material was freeze-dried. The compound **H-C10** was obtained after preparative **[Prep-HPLC]** purification (gradient: 0 min, 100% A, 0% B; 5 min, 100% A, 0% B; 45 min, 0% A, 100% B). 89 mg were obtained 30%). **[HPLC 1]** t_R: 3,70 min. **HR-ESI-MS** m/z calcd for $[C_{33}H_{67}N_{17}O_6+2H]^{2+}$ 399.7803, found 399.7802; $[C_{33}H_{67}N_{17}O_6+3H]^{3+}$ 266.8560, found 266.8566. **^{1}H NMR** (400 MHz, D_2O) δ_H: 4.44 – 4.22 (m, 4H, Hα), 3.29 – 3.12 (m, 6H, $H\delta_R$), 2.56 – 2.36 (m, 2H, $H\gamma_{E^*}$), 2.29 (t, ^3J = 7.2, 2H, CH_2-CO(H2)), 2.20 – 2.06 (m, 1H, $H\beta_{E^*}$), 2.06 – 1.94 (m, 1H, $H\beta'_{E^*}$), 1.94 – 1.50 (m, 6H, $H\beta_R$) (m, 6H, $H\gamma_R$) (m, 2H, CH_2(H3)), 1.39 – 1.15 (m, 12H, CH_2(H4-H9)), 0.85 (t, ^3J = 6.6, 3H, CH_3(H10)).

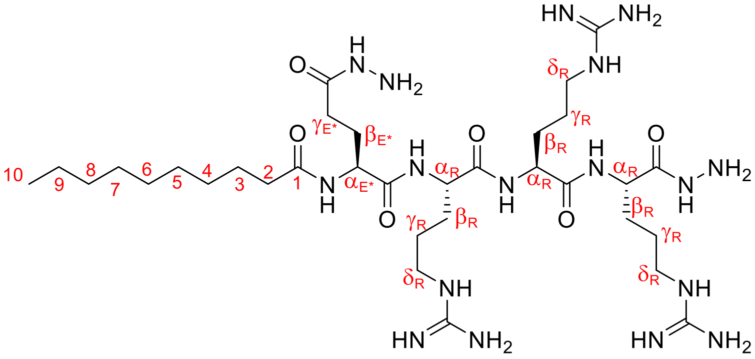

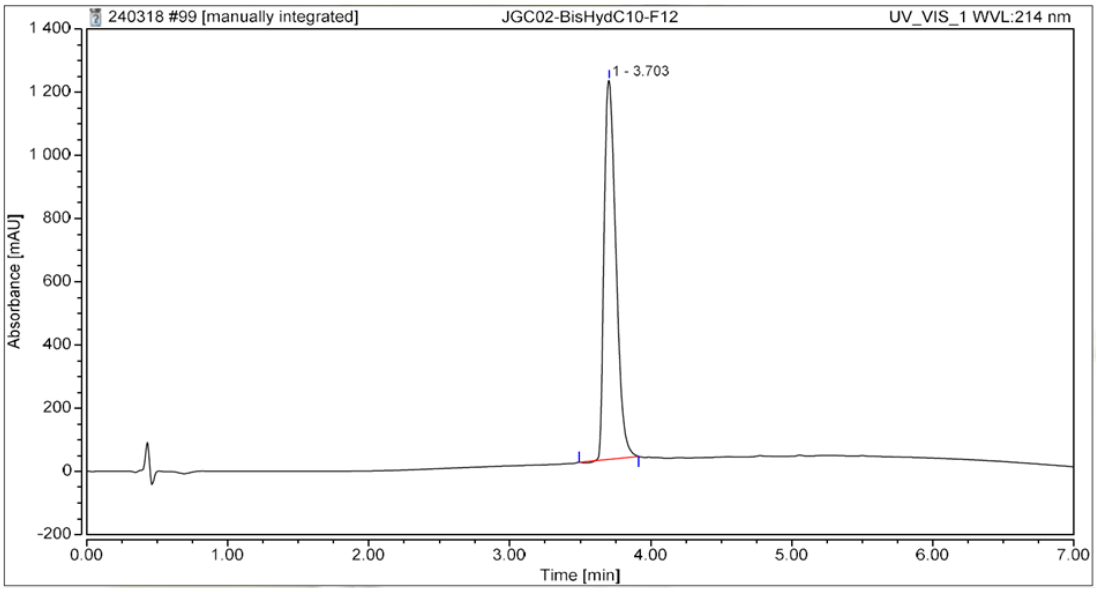

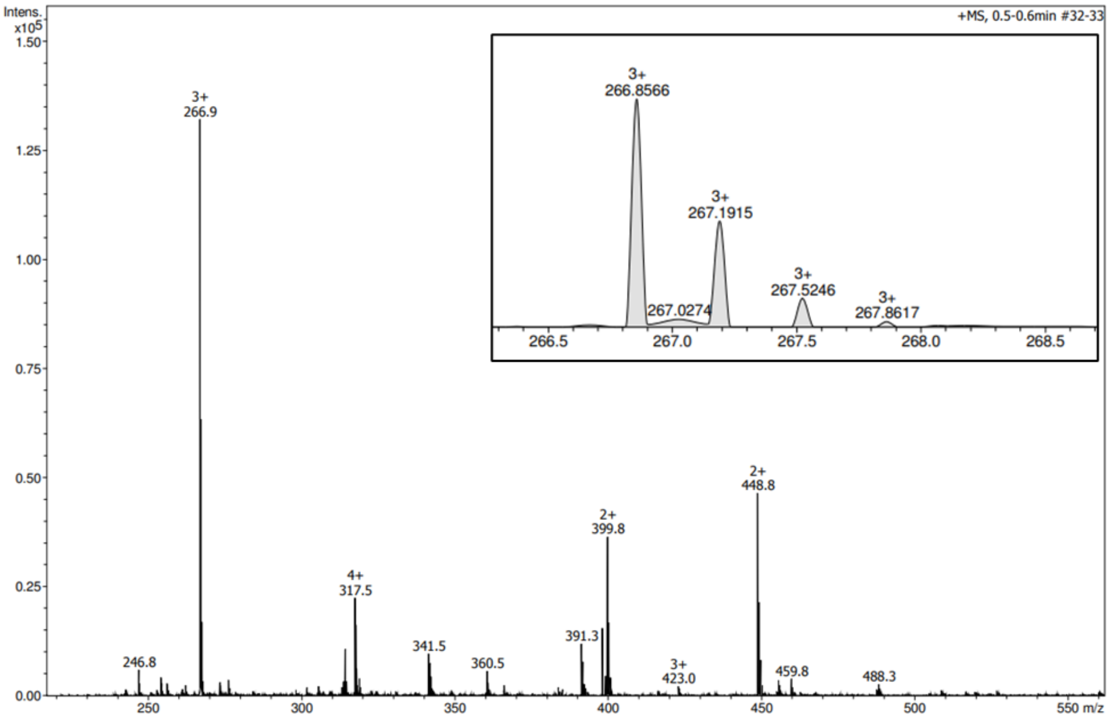

Figure 26. HPLC chromatogram (top) and HR-ESI-MS spectra (bottom) of compound **H-C10**.

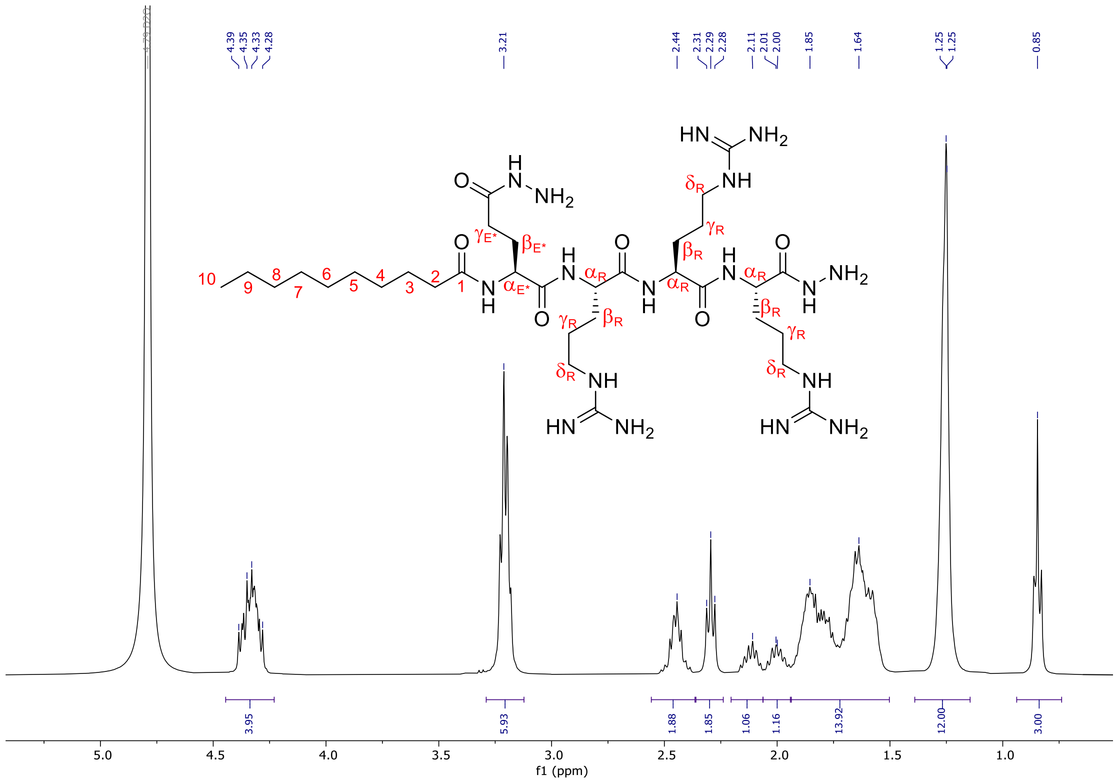

Figure 27. ^{1}H NMR (D_2O) spectrum of compound **H-C10**.

d) Compound H-C12

The peptide was synthesized according to the general **[SPPS 2]** procedure using Fmoc-*L*-Arg(Pbf)-OH, Fmoc-*L*-Glu[NHNH(2Cl-Trt)]-OH and dodecanoic acid. The solution was then concentrated, precipitated by adding ice cold Et_2O, and centrifuged. The supernatant was removed, and the crude material was freeze-dried. The compound **H-C12** was obtained after preparative **[Prep-HPLC]** purification (gradient: 0 min, 100% A, 0% B; 5 min, 100% A, 0% B; 45 min, 0% A, 100% B). 6 mg were obtained (2%). **[HPLC 1]** t_R: 4.26 min. **HR-ESI-MS** m/z calcd for $[C_{35}H_{71}N_{17}O_6+H]^+$ 826.5846, found 826.5827; $[C_{29}H_{59}N_{17}O_6+2H]^{2+}$ 371.75, found 371.75; $[C_{35}H_{71}N_{17}O_6+2H]^{2+}$ 413.7966, found 413.7961; $[C_{35}H_{71}N_{17}O_6+3H]^{3+}$ 276.2011, found 276.2007. **^{1}H NMR** (D_2O) δ_H: 4.39 – 4.30 (m, 3H, Hα_R), 4.30 – 4.26 (m, 1H, Hα_{E^*}), 3.29 – 3.15 (m, 6H, Hδ_R), 2.53 – 2.37 (m, 2H, Hγ_{E^*}), 2.31 (t, ^3J = 7.2, 2H, CH_2-CO (H2)), 2.19 – 2.08 (m, 2H, Hβ_{E^*}), 2.05 – 1.93 (m, 2H, Hβ'_{E^*}), 1.93 – 1.72 (m, 6H, Hβ_R), 1.72 – 1.52 (m, 6H, Hγ_R) (m, 2H, CH_2(H3)), 1.35 – 1.20 (m, 16H, CH_2(H4-H11)), 0.86 (t, ^3J = 7.0, 3H, CH_3 (H12)).

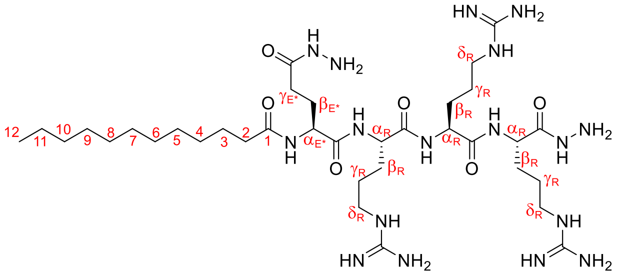

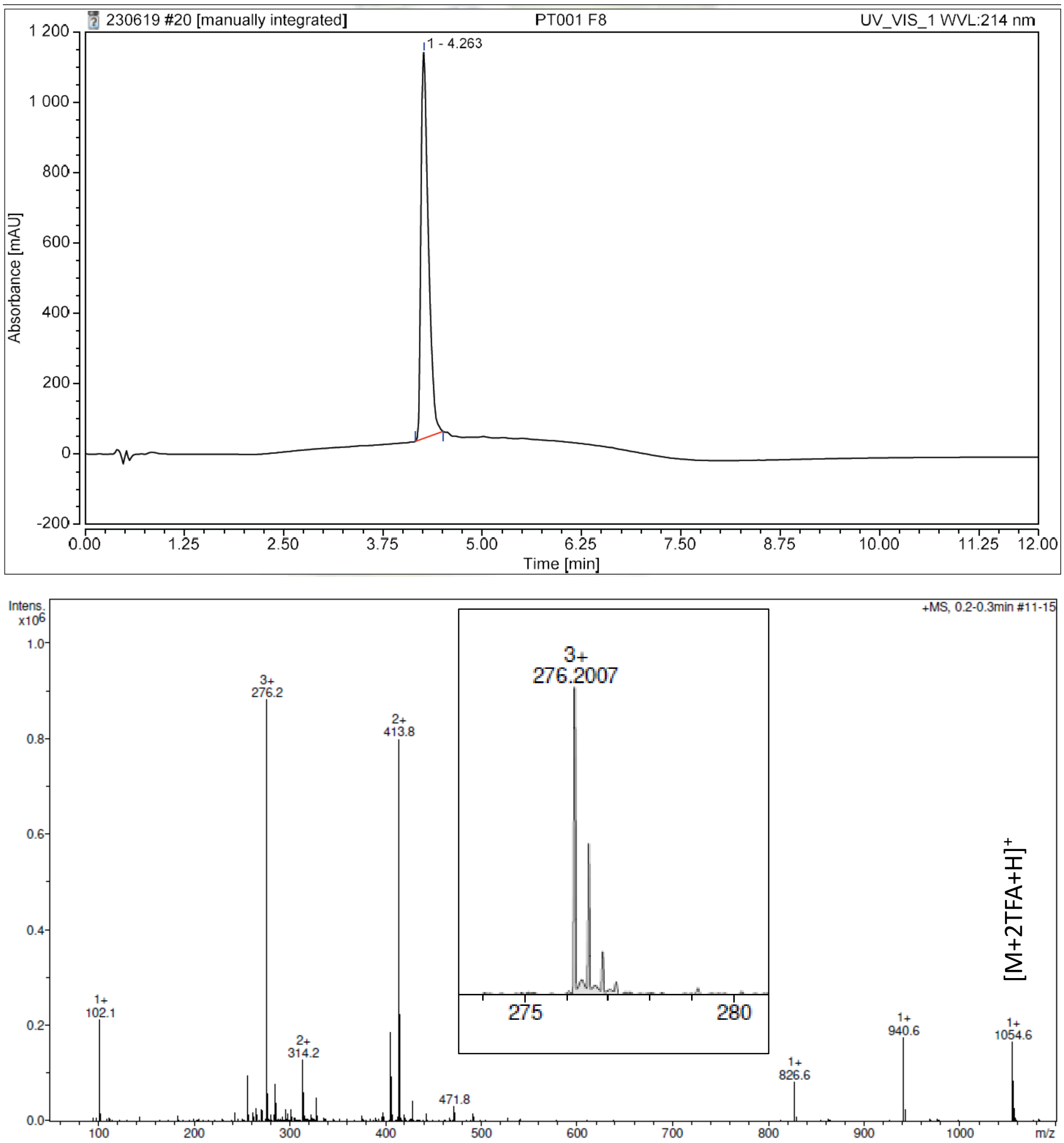

Figure 28. HPLC chromatogram (top) and HR-ESI-MS spectra (bottom) of compound **H-C12**.

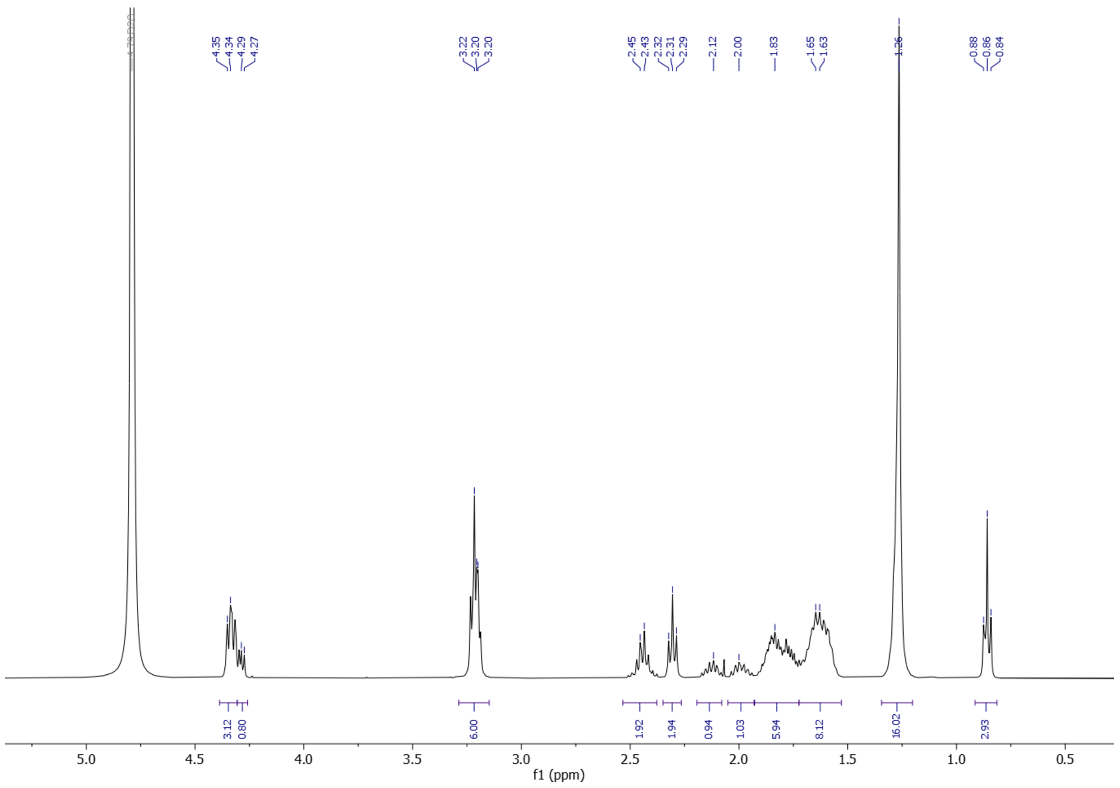

Figure 29. ^{1}H NMR (D_2O) spectrum of compound **H-C12**.

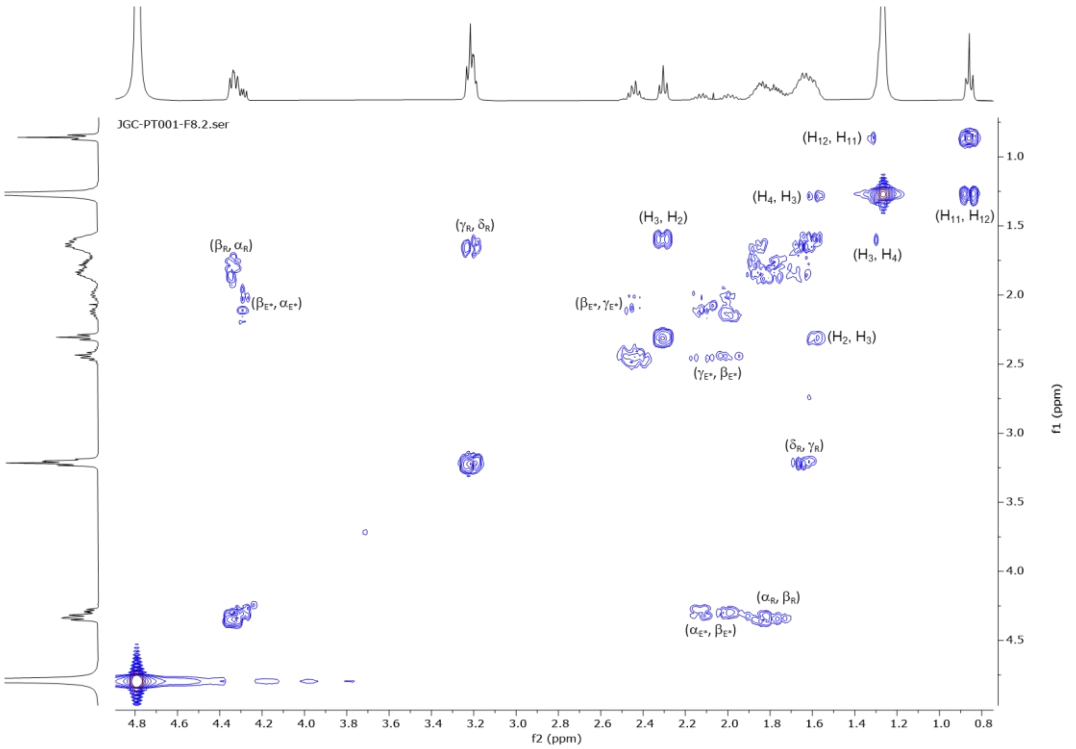

Figure 30. ^{1}H COSY NMR (400 MHz, D_2O) spectrum of compound **H-C12**.

e) Compound H-C18

The peptide was synthesized according to the general **[SPPS 2]** procedure using Fmoc-*L*-Arg(Pbf)-OH, Fmoc-*L*-Glu[NHNH(2Cl-Trt)]-OH and octadecanoic acid. The solution was then concentrated, precipitated by adding ice cold Et_2O, and centrifuged. The supernatant was removed, and the crude material was freeze-dried. The compound **H-C18** was obtained after preparative **[Prep-HPLC]** purification (gradient: 0 min, 70% A, 30% B; 5 min, 70% A, 30% B; 45 min, 0% A, 100% B). 7 mg were obtained (3%). **[HPLC 1]** t_R: 5.21 min. **HR-ESI-MS** m/z calcd for $[C_{41}H_{83}N_{17}O_6+H]^+$ 910.6785, found 910.6775; $[C_{41}H_{83}N_{17}O_6+2H]^{2+}$ 455.8429, found 455.8434; $[C_{41}H_{83}N_{17}O_6+3H]^{3+}$304.2, found 304.2. **^{1}H NMR** (MeOD) δ_H: 4.40 – 4.29 (m, 3H, $H\alpha_R$), 4.29 – 4.22 (m, 1H, $H\alpha_{E^*}$), 3.26 – 3.16 (m, 6H, $H\delta_R$), 2.34 (t, ^3J = 7.5, 2H, $H\gamma_{E^*}$), 2.25 (t, ^3J = 7.2, 2H, CH_2-CO(H2)), 2.15 – 2.03 (m, 2H, $H\beta_{E^*}$), 2.03 – 1.95 (m, 2H, $H\beta'_{E^*}$), 1.95 – 1.54 (m, 6H, $H\beta_R$) (m, 6H, $H\gamma_R$) (m, 2H, CH_2(H3)), 1.36 – 1.25 (m, 28H, CH_2(H4-H17)), 0.90 (t, ^3J = 6.9, 3H, CH_3(H18)).

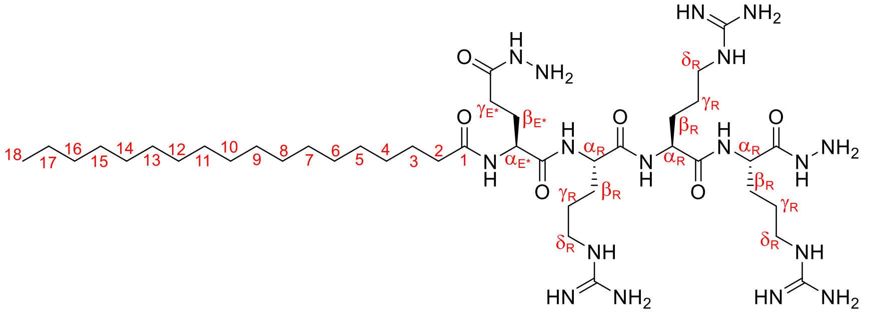

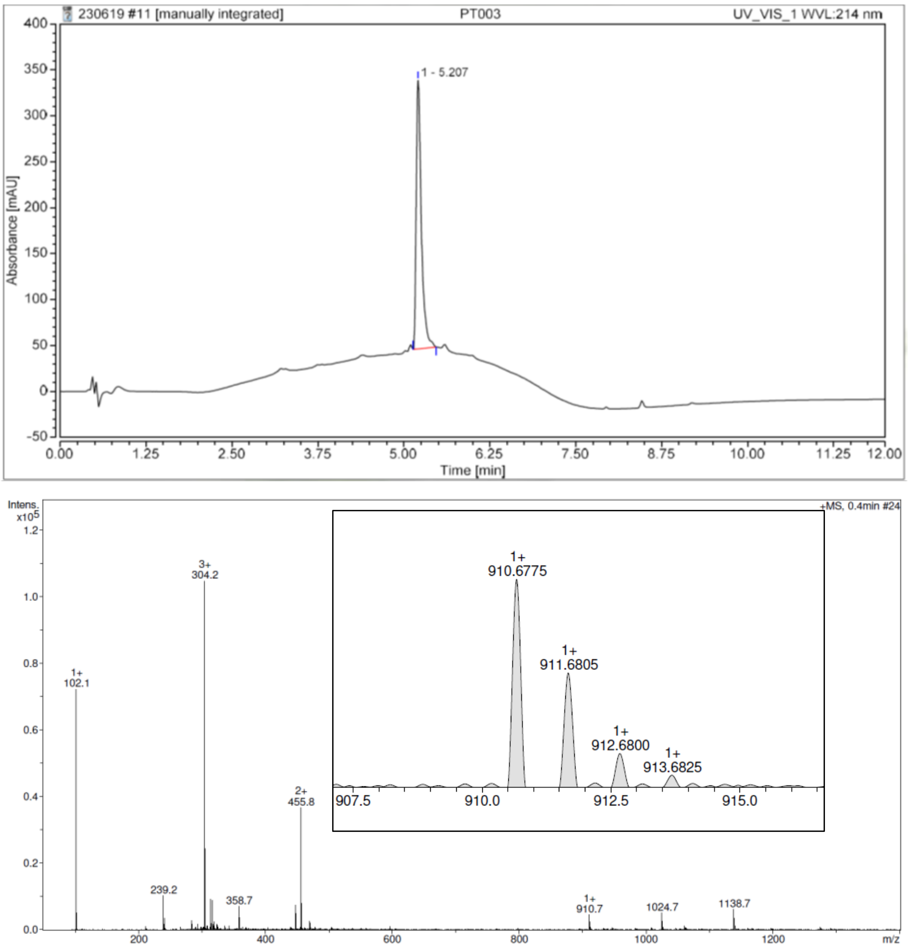

Figure 31. HPLC chromatogram (top) and HR-ESI-MS spectra (bottom) of compound **H-C18**.

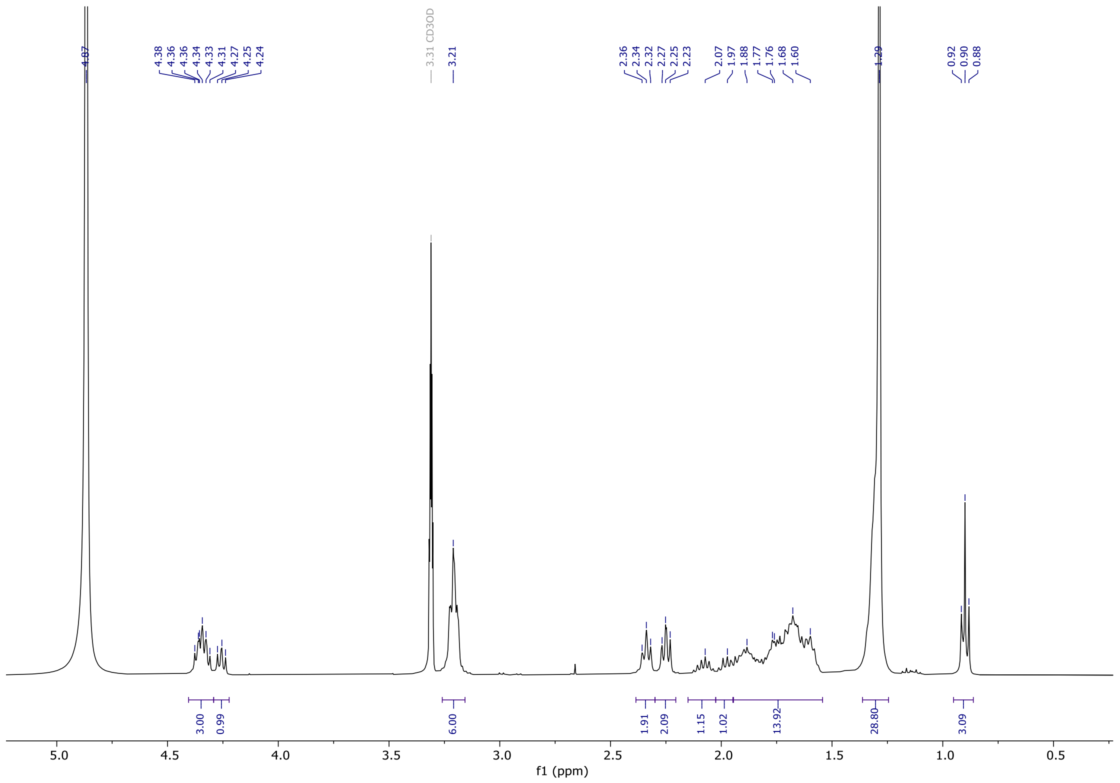

Figure 32. ^{1}H NMR (MeOD) spectrum of compound **H-C18**.

5. MALDI-TOF mass spectrometry

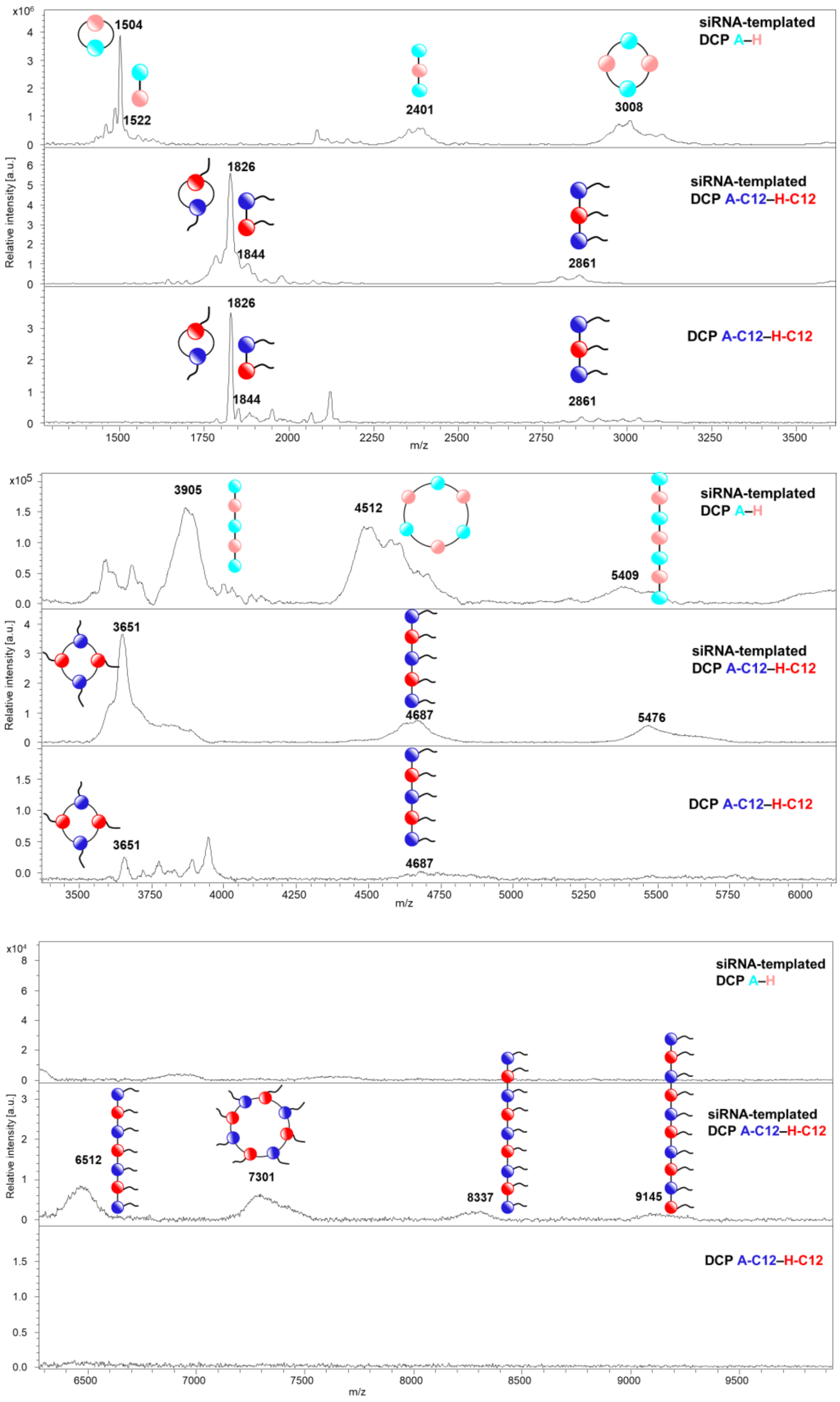

Figure 33. Zoom of MALDI-TOF spectra characterizing the DCPs: i) formed by siRNA-templated polymerization: DCP **A–H** (top) and DCP **A-C12–H-C12** (middle); and ii) formed through non-templated assembly: DCP **A-C12–H-C12** (bottom).

	DP	DCP Composition	m/z Calculated	m/z found (w/ siRNA, N/P 5)	m/z found (w/o siRNA)
Non-amphiphilic	2	Cyclic [**1**A+**1**H]$^+$	1504.89	1504	
	2	Linear [**1**A+**1**H]$^+$	1522.90	1522	
	3	Linear [**2**A+H]$^+$	2401.38	2401	
	4	Cyclic [**2**A+**2**H]$^+$	3007.76	3008	
	5	Linear [**3**A+**2**H]$^+$	3904.25	3905	
	6	Cyclic [**3**A+**3**H]$^+$	4512.64	4512	
	7	Linear [**4**A+**3**H]$^+$	5409.13	5409	
Amphiphilic	2	Cyclic [**1**A-C12+**1**H-C12]$^+$	1826.23	1826	1826
	2	Linear [**1**A-C12+**1**H-C12]$^+$	1844.24	1844	1844
	3	Linear [**2**A-C12+**1**H-C12]$^+$	2861.90	2861	2861
	4	Cyclic [**2**A-C12+**2**H-C12]$^+$	3651.45	3651	3651
	5	Linear [**3**A-C12+**2**H-C12]$^+$	4687.12	4687	4687
	6	Cyclic [**3**A-C12+**3**H-C12]$^+$	5476.66	5476	n.d.
	7	Linear [**4**A-C12+**3**H-C12]$^+$	6512.33	6512	n.d.
	8	Cyclic [**4**A-C12+**4**H-C12]$^+$	7301.88	7301	n.d.
	9	Linear [**5**A-C12+**4**H-C12]$^+$	8337.55	8337	n.d.
	10	Linear [**5**A-C12+**5**H-C12]$^+$	9145.11	9145	n.d.

Table 1. List of calculated and experimental m/z for the cyclic and linear oligomers of varying degree of polymerization (DP), which form upon mixing complementary bisaldehyde and bishydrazide monomers. All products ionize as protonated cations.

6. SiRNA delivery

a) Fluorescence imaging

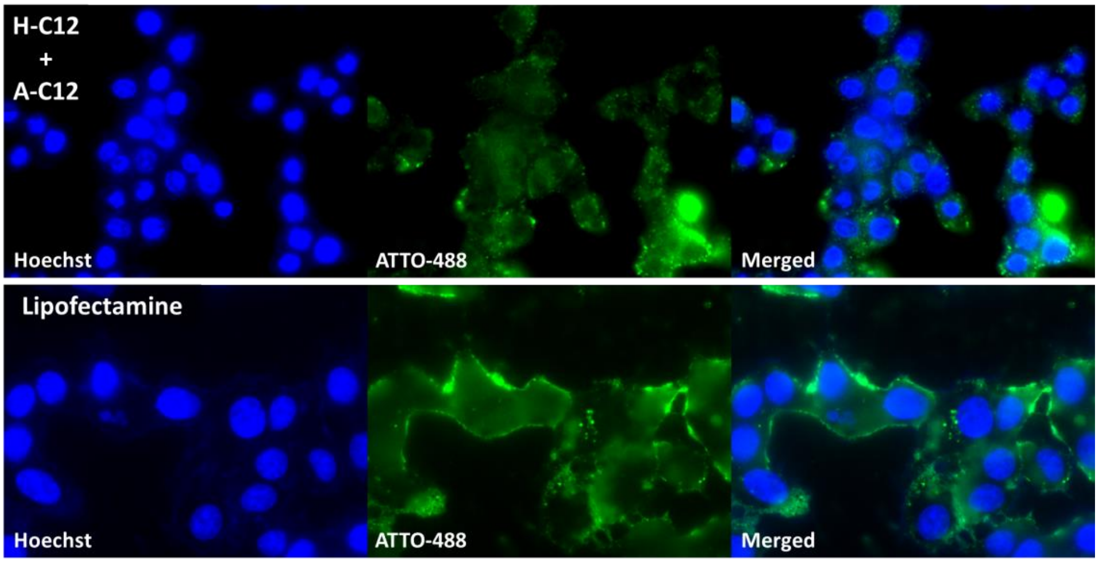

Figure 34. Fluorescence microscopy of siRNA-atto488 delivery into MCF-7 cells by C12 hydrophobized DCPs at N/P 20 (top) and lipofectamine (bottom) at a siRNA dose of 100 nM, 4 hours of incubation post-transfection. Cell nucleic were stained with DAPI dye (Hoeschst, blue channel: ex 357/44 nm, em 447/60 nm), and the siRNA-atto488 was detected in the green channel (Ex 482/25 nm; Em 524/24 nm).

b) LDH assay

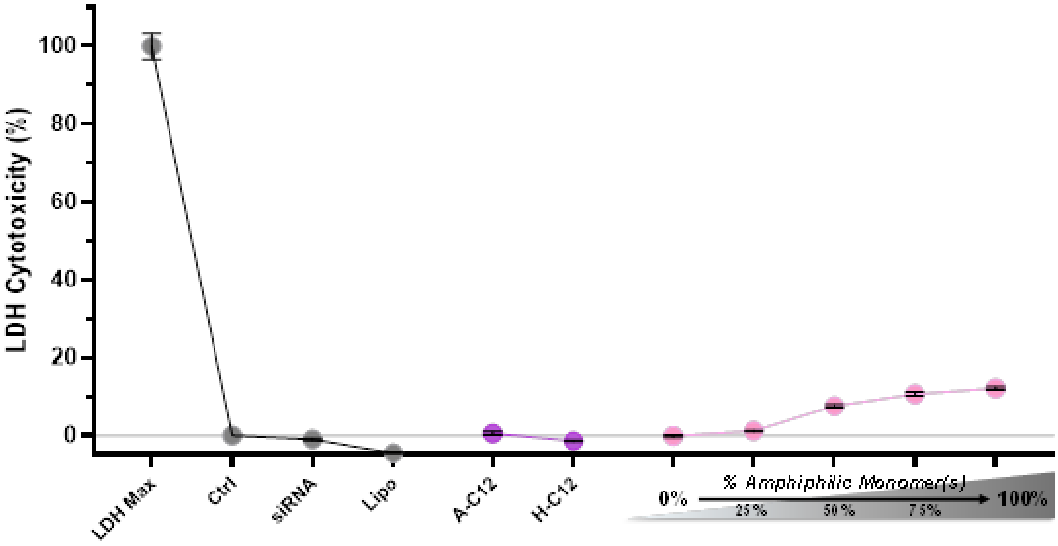

Figure 35. LDH cytotoxicity assay in MCF7-Luc cells of siRNA polyplexes formed from 4-components amphiphilic DCPs ((100-x)% (A+H) + x% (**A-C12**+**H-C12**): pink circles), compared to siRNA alone (negative control), lipofectamine (positive control), **A-C12** alone, and **H-C12** alone. The cytotoxicity was normalized against LDH Max (Cell lysis). Absorbance readout was performed 72 h after transfection. Data shows the mean ± S.E.M. of conditions performed in triplicates. The experiments with DCPs were carried out at N/P 20 and those with the monomers **A-C12** and **H-C12** alone were therefore performed at N/P 10. SiRNA dose: 100 nM

c) Dose dependent C12-DCP activity

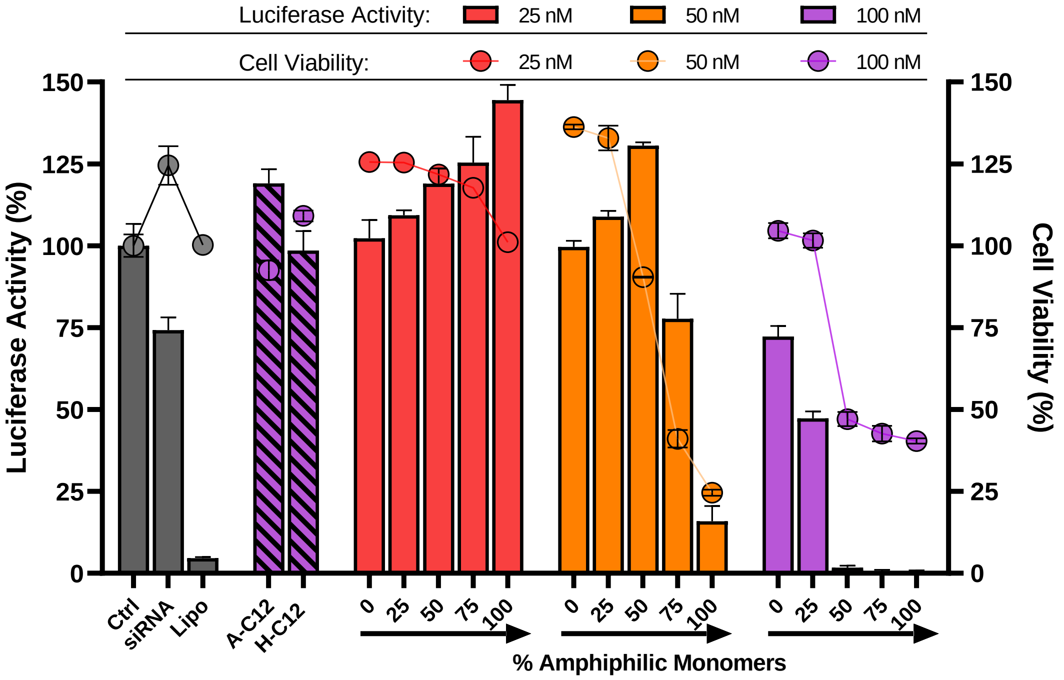

Figure 36. siRNA Dose dependent luciferase activity knockdown and Cell Viability of C12 Amphiphilic DCPs (red 25 nM, orange 50 nM and purple 100 nM). MCF7-Luc cells at N/P ratio of 20 with the corresponding DCP.

d) 4-component C10-DCP activity

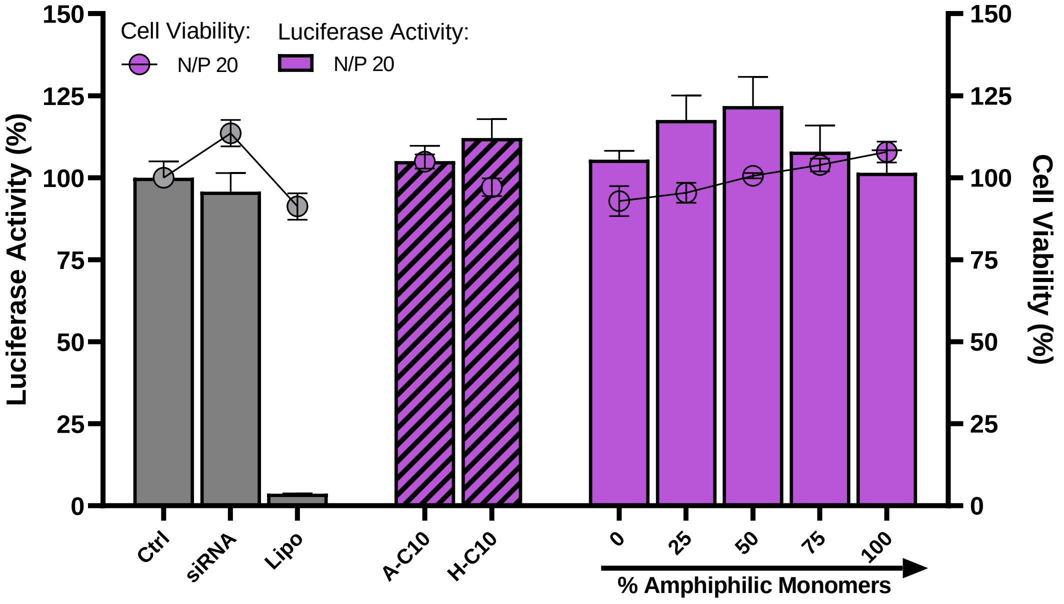

Figure 37. Luciferase activity knockdown and cell viability in MCF7-Luc cells of siRNA polyplexes formed from 4-components amphiphilic DCPs ((100-x)% (A+H) + x% (A-C10+H-C10): purple bars/circles), compared to siRNA alone (negative control), lipofectamine (positive control), A-C10 alone, and H-C10 alone. Luminescence readout was performed 72 h after transfection. Data shows the mean ± S.E.M. of conditions performed in triplicates. The experiments with DCPs were carried out at N/P 20 and those with the monomers A-C10 and H-C10 alone were therefore performed at N/P 10. SiRNA dose: 100 nM.

7. DLS & ζ Potential

	A-12 + H	H-12 + A	H + A
**Correlogram**	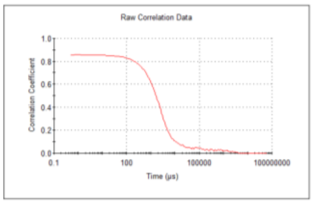	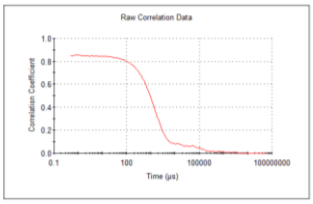	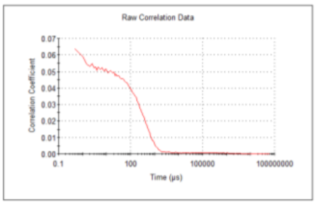
**Size Distribution (Intensity)**	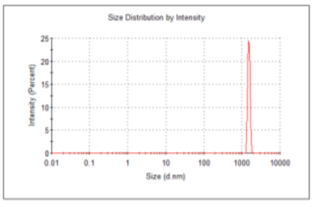	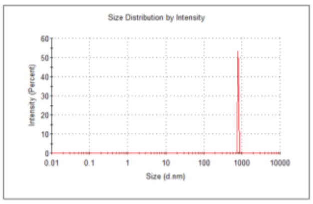	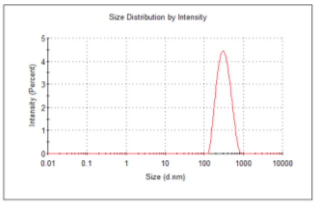
**ζ-Potential Distribution**	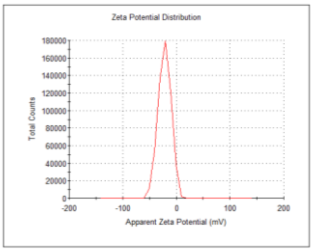	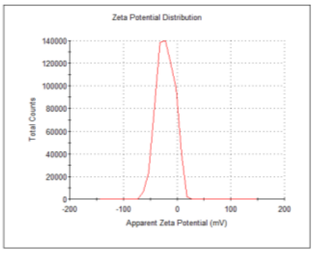	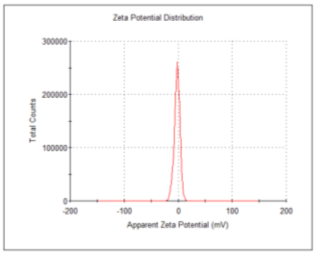

Table 2. DLS and ζ Potential studies of 2-component mixtures at N/P 20.

**% C_{12}**	**25**	**50**	**75**	**100**
**Correlogram**	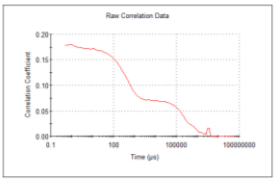	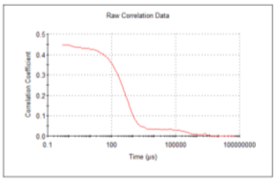	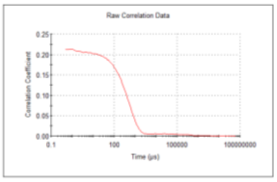	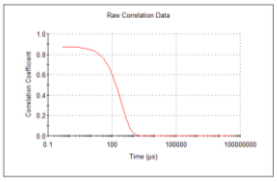
**Size Distribution (Intensity)**	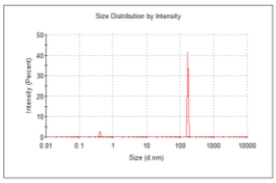	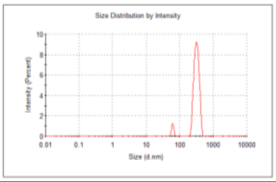	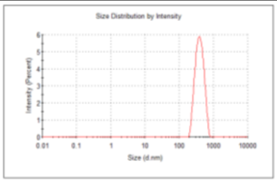	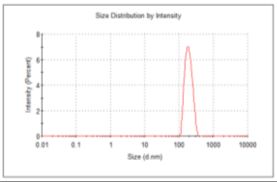
**Size Distribution (Number)**	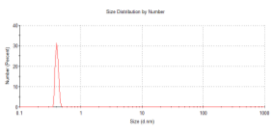	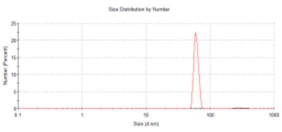	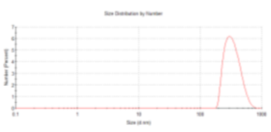	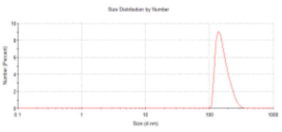
**ζ-Potential Distribution**	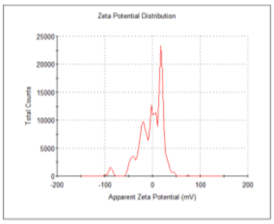	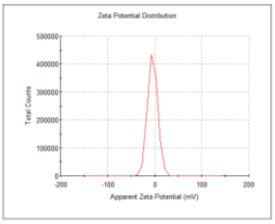	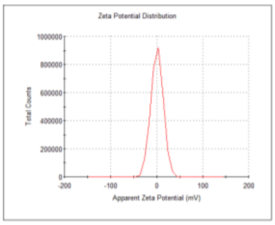	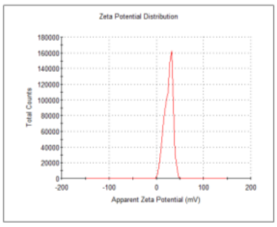

Table 3. DLS and ζ Potential studies of 4-components C12-DCPs at N/P 20

% C_{12}	25	50	75	100
**Correlogram**	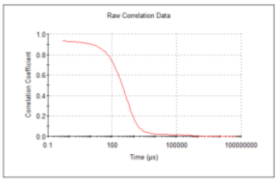	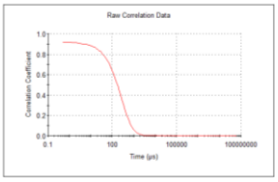	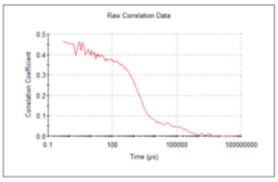	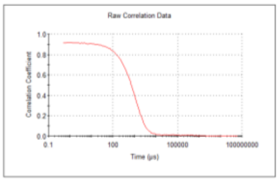
**Size Distribution (Intensity)**	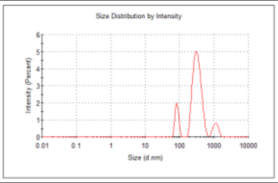	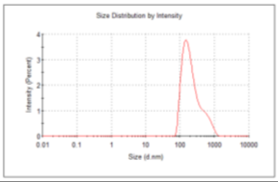	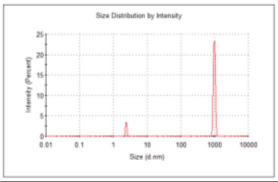	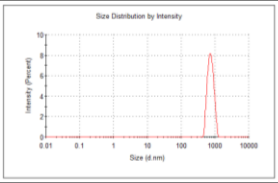
**ζ-Potential Distribution**				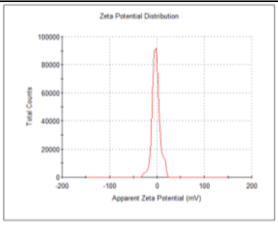

Table 4. DLS and ζ Potential studies of 4-components C12-DCPs at N/P 10.

% C_{12}	25	50	75	100
**Correlogram**	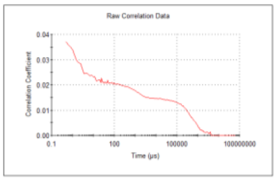	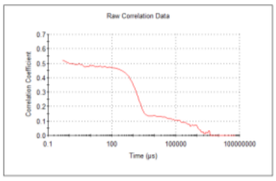	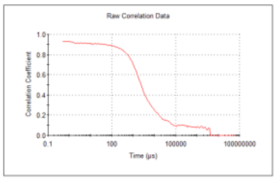	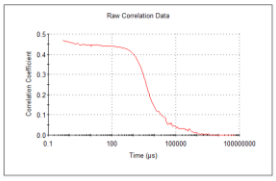
**Size Distribution (Intensity)**	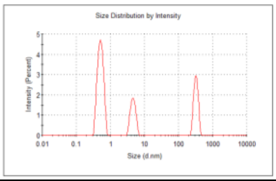	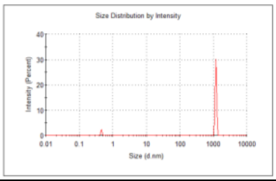	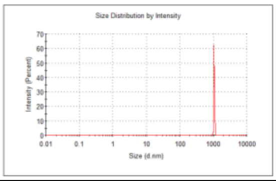	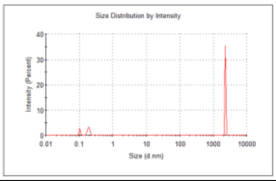
**ζ-Potential Distribution**				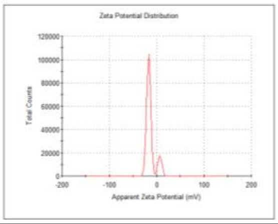

Table 5. DLS and ζ Potential studies of 4-components C12-DCPs at N/P 5.

Sample (w/siRNA)	% C_x	N/P	Size (nm)	PDI	ζ Potential (mV)
**A-C6 + H-C6**	100	20	719 ± 73	0.18	-6 ±12
**A-C18**	100	10	198 ± 132	0.39	+4 ± 12
**H-C18**	100	10	565 ± 115	0.44	+11 ± 9
**(100-x)% (A + H) + x% (A-C18 + H-C18)**	10	20	368 ± 112	0.36	+19 ± 10
	20	20	525 ± 79	0.11	+14 ± 23
	30	20	235 ± 76	0.20	+19 ± 14
	40	20	202 ± 117	0.22	+22 ± 13
	50	20	199 ± 95	0.19	+20 ± 9
	100	20	474 ± 67	0.42	+56 ± 5

Table 6. DLS analysis and ζ potential measurements of C18 and C6 polyplexes.

% C_6	Correlogram	Size Distribution (Intensity)	ζ-Potential Distribution
100	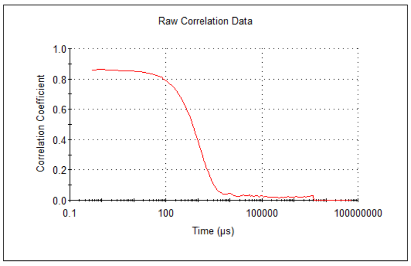	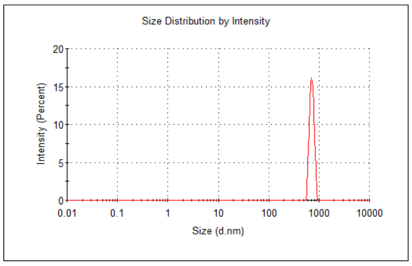	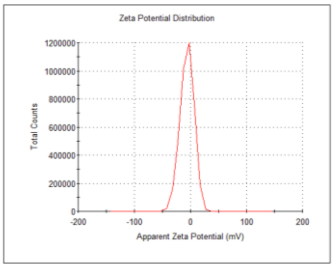

Table 7. DLS and ζ Potential studies of 100% C6 polyplex at N/P 20.

	Correlogram	Size Distribution (Intensity)	ζ-Potential Distribution
A-C18	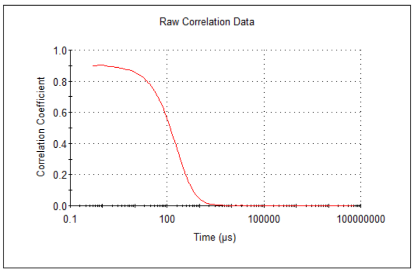	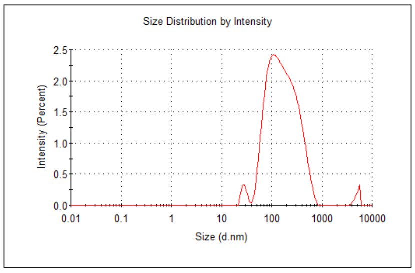	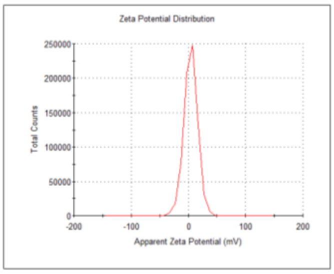
H-C18	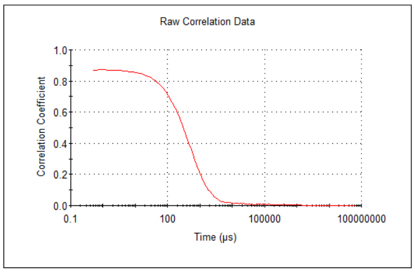	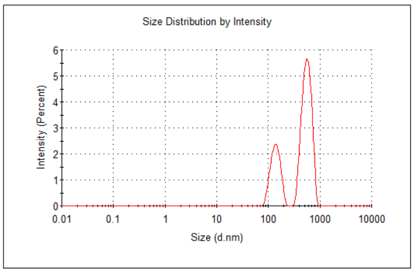	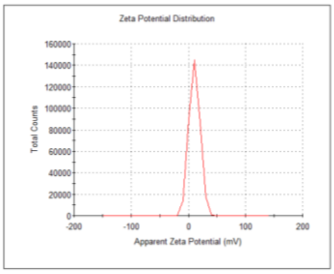

Table 8. DLS and ζ Potential studies of C18 Monomer polyplexes at N/P 10.

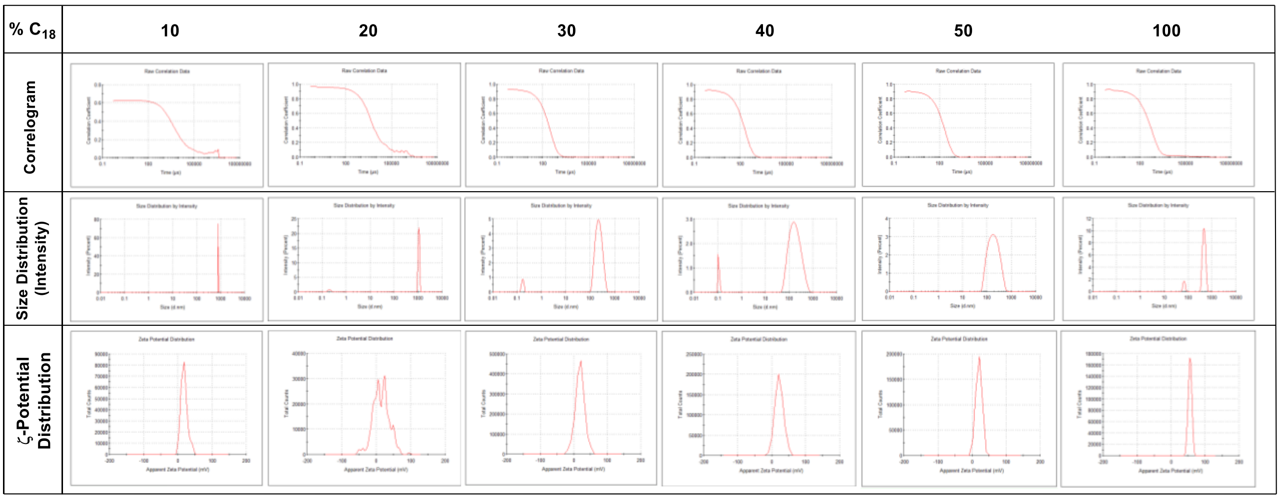

Table 9. DLS and ζ Potential studies of 4-components C18-DCPs at N/P 20.

8. TEM

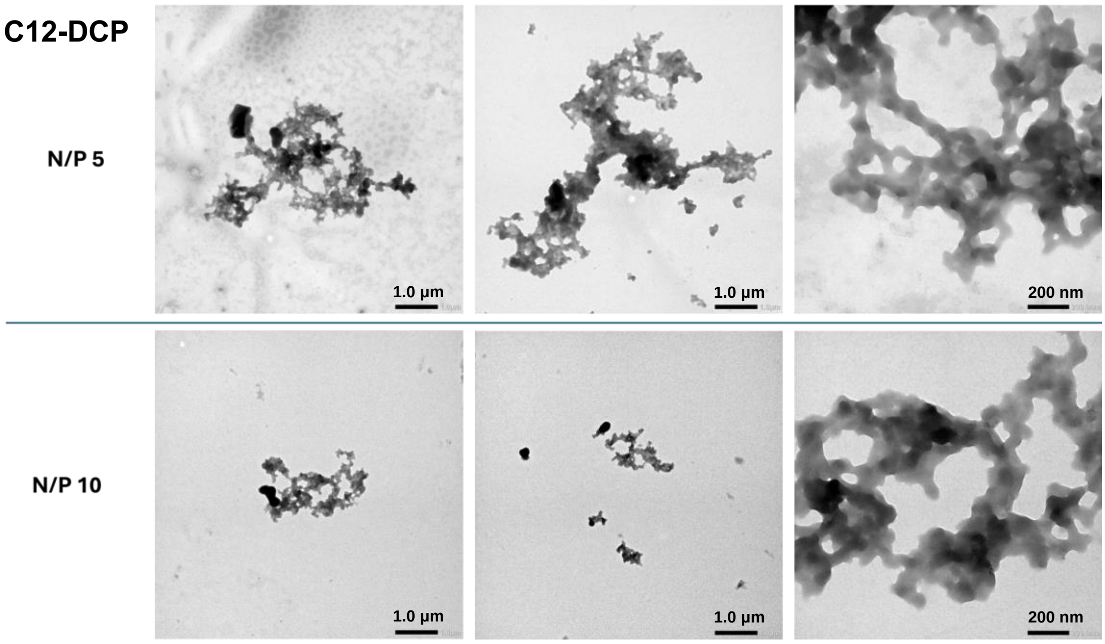

Figure 38. TEM images of **DCP A-C12–H-C12** formed in presence of siRNA at N/P 5 (top), and N/P 10 (bottom). Polyplex formation was performed overnight at a monomer concentration of 1 mM in sodium acetate buffer pH 5.5.

9. References

1 Y. C. Huang, C. C. Chen, S. J. Li, S. Gao, J. Shi and Y. M. Li, Tetrahedron, 2014, **70**, 2951–2955.

2 E. Bartolami, Y. Bessin, N. Bettache, M. Gary-Bobo, M. Garcia, P. Dumy and S. Ulrich, Org. Biomol. Chem., 2015, **13**, 9427–9438.

3 D.-D. Su, L. M. A. Ali, M. Coste, N. Laroui, Y. Bessin, M. Barboiu, N. Bettache and S. Ulrich, Chem. Eur. J., 2023, **29**, e202202921.

4 N. G. Barnes, K. Nyandoro, H. Jin and D. Macmillan, Chem. Commun., 2021, **57**, 1006–1009.

5 P. Kumar, A. Nagarajan, P. D. Uchil, Cold Spring Harb Protoc. 2018, 6, doi: 10.1101/pdb.prot095505.
